# Supplementary material for: Unexpected contribution of fibroblasts to muscle lineage as a mechanism for limb muscle patterning
Source: Nat Commun. 2021 Jun 22;12:3851. doi: 10.1038/s41467-021-24157-x (PMC8219714; doi:10.1038/s41467-021-24157-x)
Supplement: Supplementary file 1 — Supplementary Information [file 41467_2021_24157_MOESM1_ESM.pdf]

SUPPLEMENTARY Figures

Esteves de Lima et al.

Unexpected contribution of fibroblasts to muscle lineage as a mechanism for limb muscle patterning

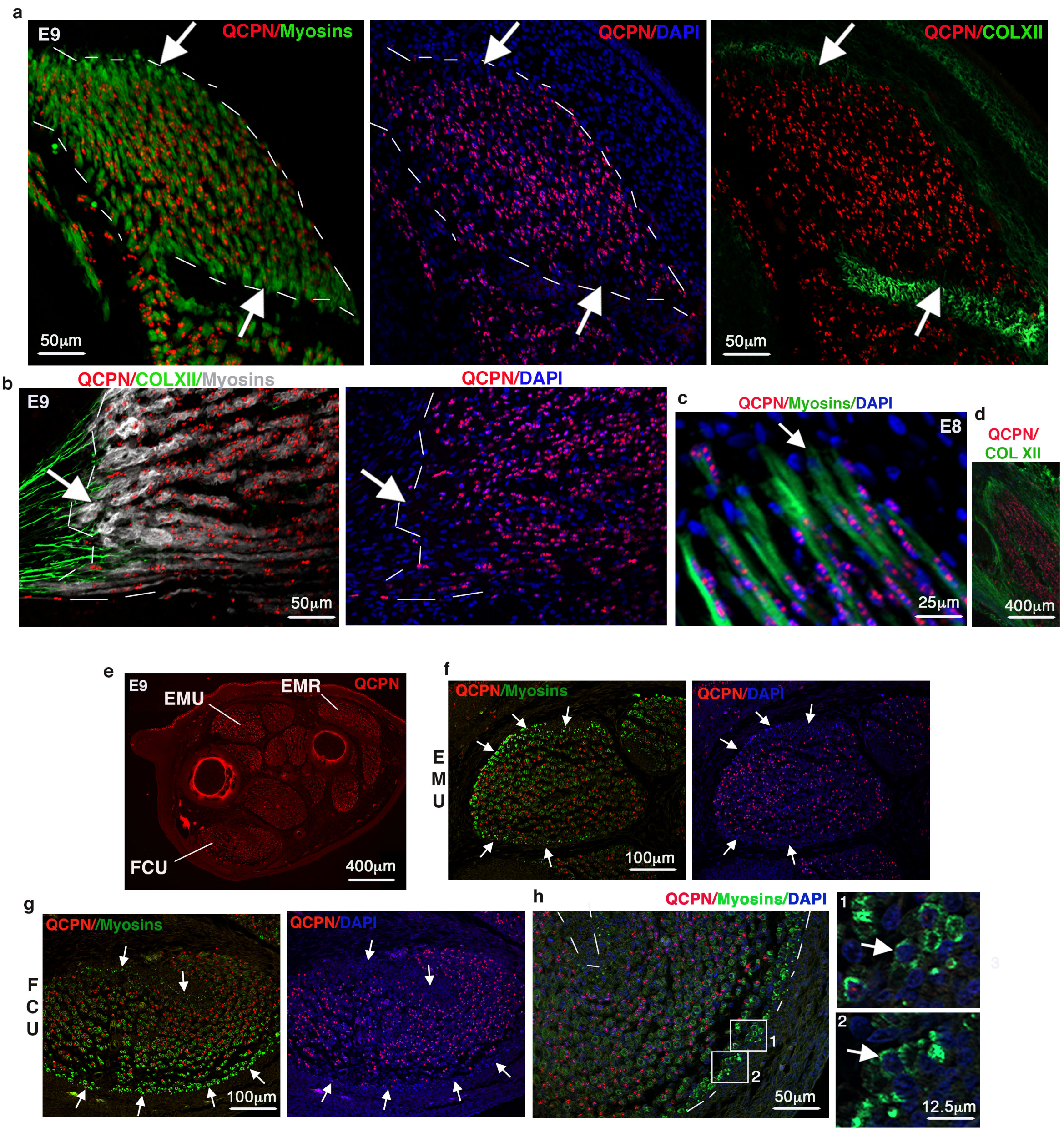

Supplementary Fig. 1

Supplementary Figure 1

A subpopulation of myonuclei in skeletal muscles is not derived from presomitic mesoderm in chicken limbs

(**a,b**) Forelimbs of presomitic grafts of E9 quail-into-chicken embryos were co-immunostained with QCPN (quail nuclei, red), MF20 (myosins, green) and ColXII (tendons, green) antibodies, combined with DAPI (blue) staining. Focus on (a) the EMR (anterior muscle) and (b) a longitudinal view of muscle. Arrows point to zones of nuclei (DAPI) that are not of quail origin at the muscle tips close to tendons. (**c,d**) Focus on muscle tips from forelimb sections of presomitic grafts quail-into-chicken fixed at E8, immunostained with QCPN (quail nuclei, red), MF20 (myosins, green) and ColXII (tendons, green) antibodies. Arrow points to a myonucleus that is QCPN negative. (**e-h**) Presomitic graft (quail-into-chicken embryos) fixed at E9. (**e**) Transverse forelimb sections immunostained with QCPN (quail nuclei, red). (**f**) Focus on the EMU muscle immunostained with QCPN (quail nuclei, red) and MF20 (myosins, green) antibodies. (**g,h**) Focus on the FCU muscle immunostained with QCPN (quail nuclei, red) and MF20 (myosins, green) antibodies. Arrows in (f,g) point to muscle tips. (h) Zoom to muscle tip regions with high magnifications showing quail-negative myonuclei (*i.e.* not presomitic-derived, arrows).

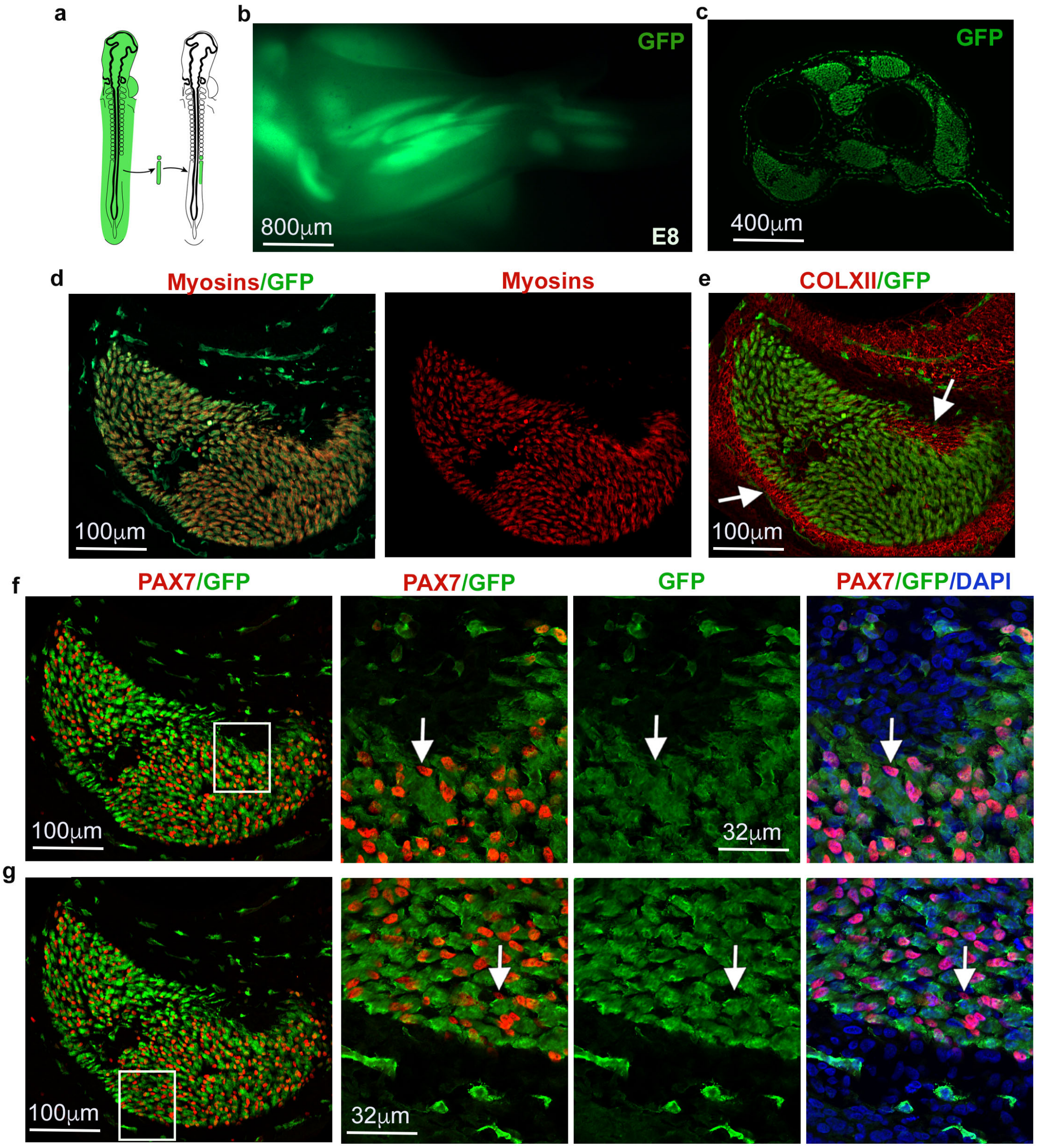

Supplementary Fig.2

Supplementary Figure 2

A subpopulation of PAX7+ cells is not derived from presomitic mesoderm in chicken limbs

(**a**) Schematic of presomitic grafts of GFPchicken into chicken embryos. **(b**) Forelimb whole-mount of presomitic graft fixed at E8 showing GFP fluorescence. **(c)** Transverse limb section showing cytoplasmic GFP expressed in presomitic-derived cells in muscles. (**d,e**) Focus on FCU muscle labelled with GFP (green), MF20 (myosins, red) (d) and ColXII (tendons, red) (e) antibodies. Arrows in (e) point to tendons. (**f,g**) Transverse section of the FCU muscle labelled with PAX7 (red), GFP (green) and DAPI (blue). High magnifications of FCU muscle tips show PAX7+ cells that are GFP negative (*i.e.* not presomitic-derived, arrows).

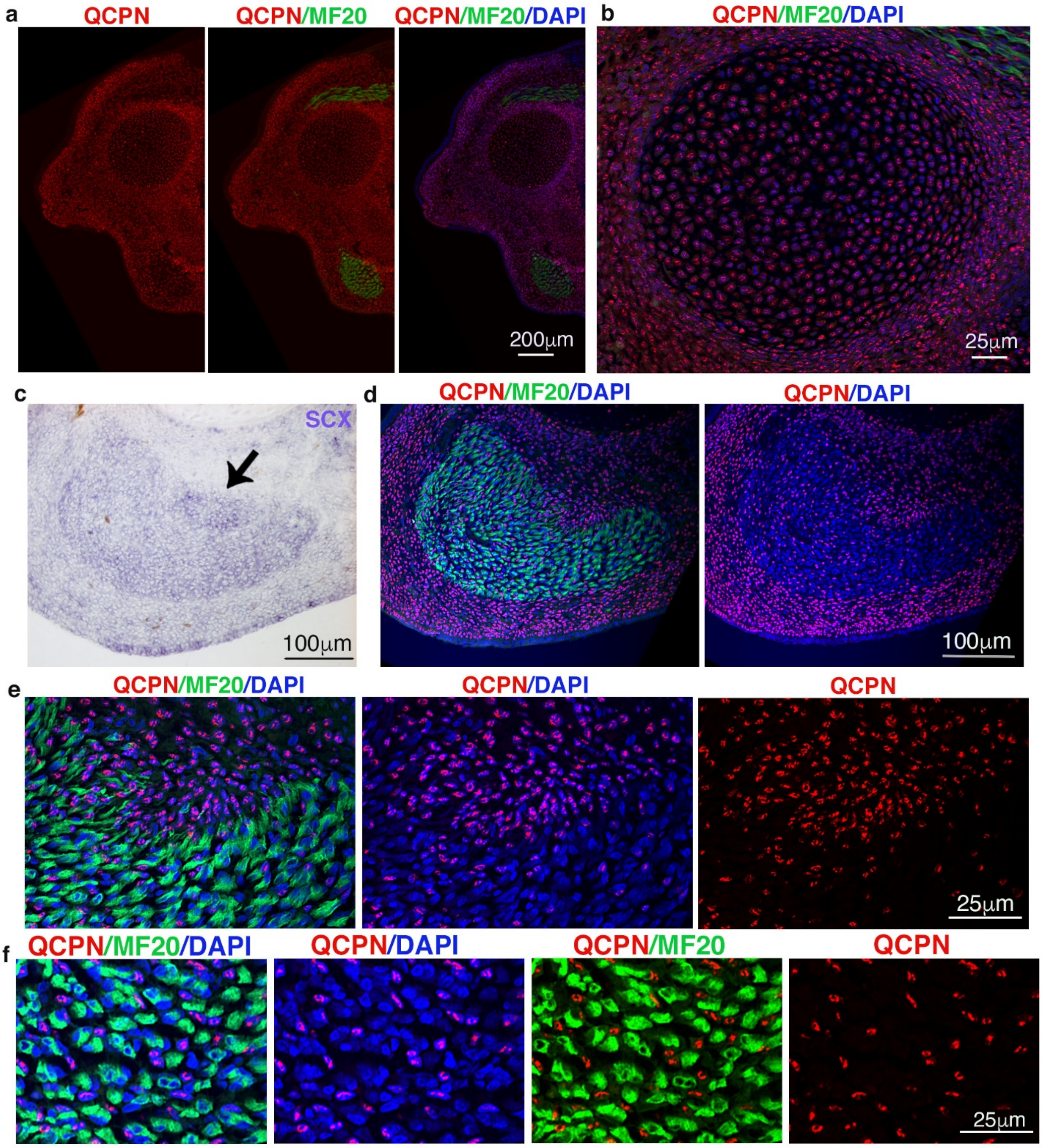

Supplementary Fig. 3

Supplementary Figure 3

Quail lateral-plate mesoderm grafts into chicken embryos label the expected derivatives in limbs

(**a**) Limb transverse sections of quail-into-chicken lateral-plate mesoderm grafts fixed at E9 and immunostained with QCPN (quail nuclei, red) and MF20 (myosins, green) antibodies, combined with DAPI (blue). On higher magnifications, the quail+ nuclei (red), which are lateral plate-derived, are observed in cartilage (**b**), tendon (**d,e**), visualised with *SCX* expression by in situ hybridization on adjacent section (**c,** arrow) and in irregular CT in between myosin+ cells inside muscle (**f**). (e) is a zoom of the tendon regions of the FCU muscle in (d).

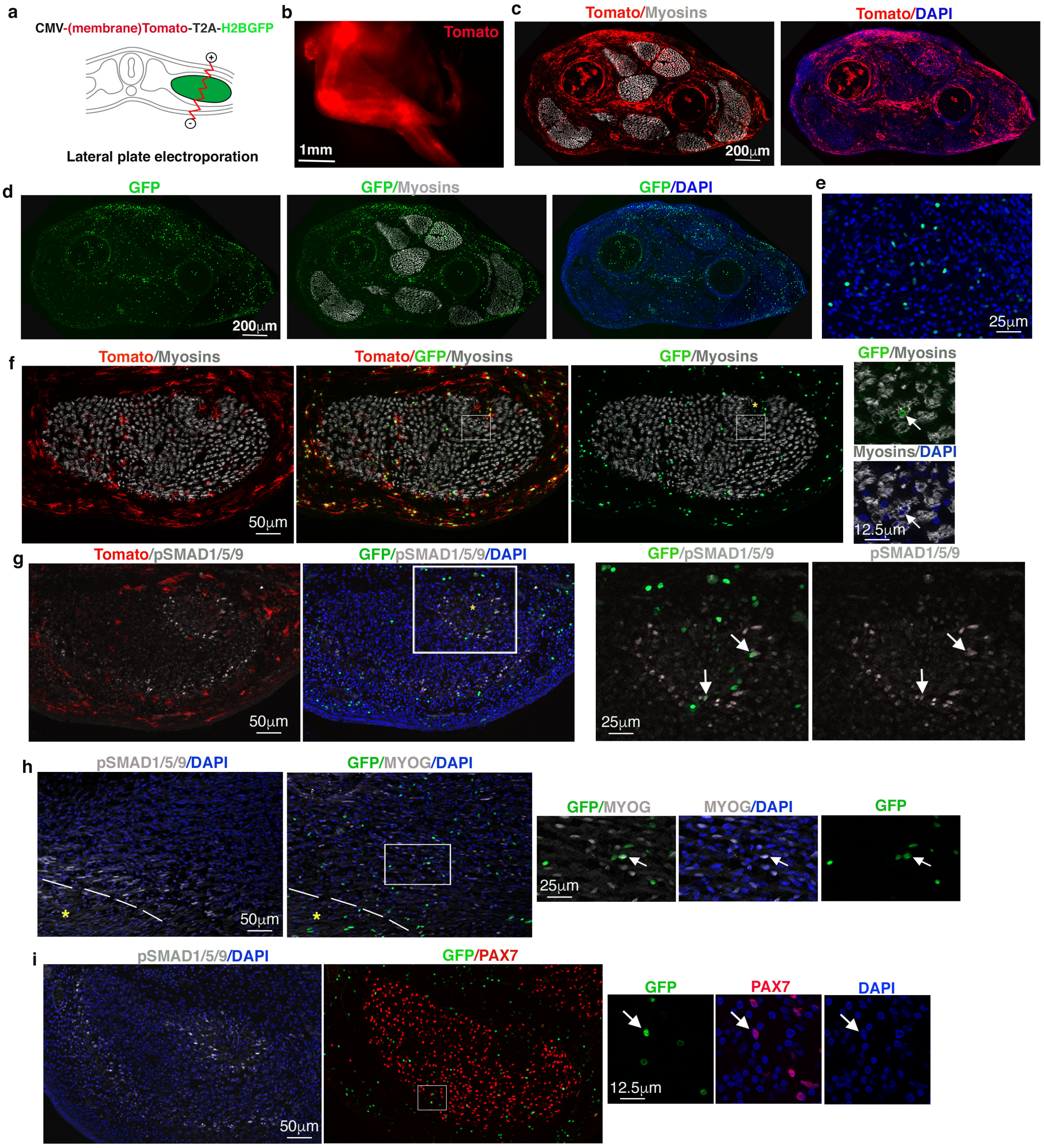

Supplementary Fig. 4

Supplementary Figure 4

Lateral-plate electroporation experiments in chicken show that a subpopulation of myogenic cells are derived from lateral plate

(**a**) Schematic of lateral-plate electroporation in E2 chicken embryos. **(b)** Whole-mount of E9 forelimb showing Tomato fluorescence after electroporation with pT2AL-CMV/βactin-membrane/Tomato-T2A-H2B/GFP and CMV/βactin-Transposase at E2. **(c,d)** Low magnifications of transverse limb sections showing Myosin (grey) labeling along with Tomato expression (red) (c) or GFP expression (green) (d). (**e**) Zoom on GFP+ cells in limb CT connective tissue (a non-muscle area). (**f,g**) Adjacent transverse sections of the FCU muscle showing GFP (green) and Tomato (red) fluorescence along with MF20 (myosins, grey) labeling (f) or along with pSMAD1/5/9 (grey) labeling (g), showing the presence of GFP+ myonuclei (f, arrows) close to muscle tips labelled with pSMAD1/5/9 (g). GFP+pSMAD+ cells are also observed (g, arrows). **(h)** Adjacent longitudinal sections of a ventral forelimb muscle showing GFP (green) fluorescence and MYOG (grey) or pSMAD1/5/9 (grey) labelings. High magnification of the boxed area in (h) shows a GFP+MYOG+ cell (arrows) close to muscle tips labelled with pSMAD1/5/9. **(i)** Adjacent transverse sections of the FCU muscle exhibiting GFP (green) fluorescence combined with PAX7 (red) or pSMAD1/5/9 (grey) labelings show the presence of a GFP+PAX7+ cell (arrows), close to the muscle borders labelled with pSMAD1/5/9. Nuclei are labeled with DAPI (blue).

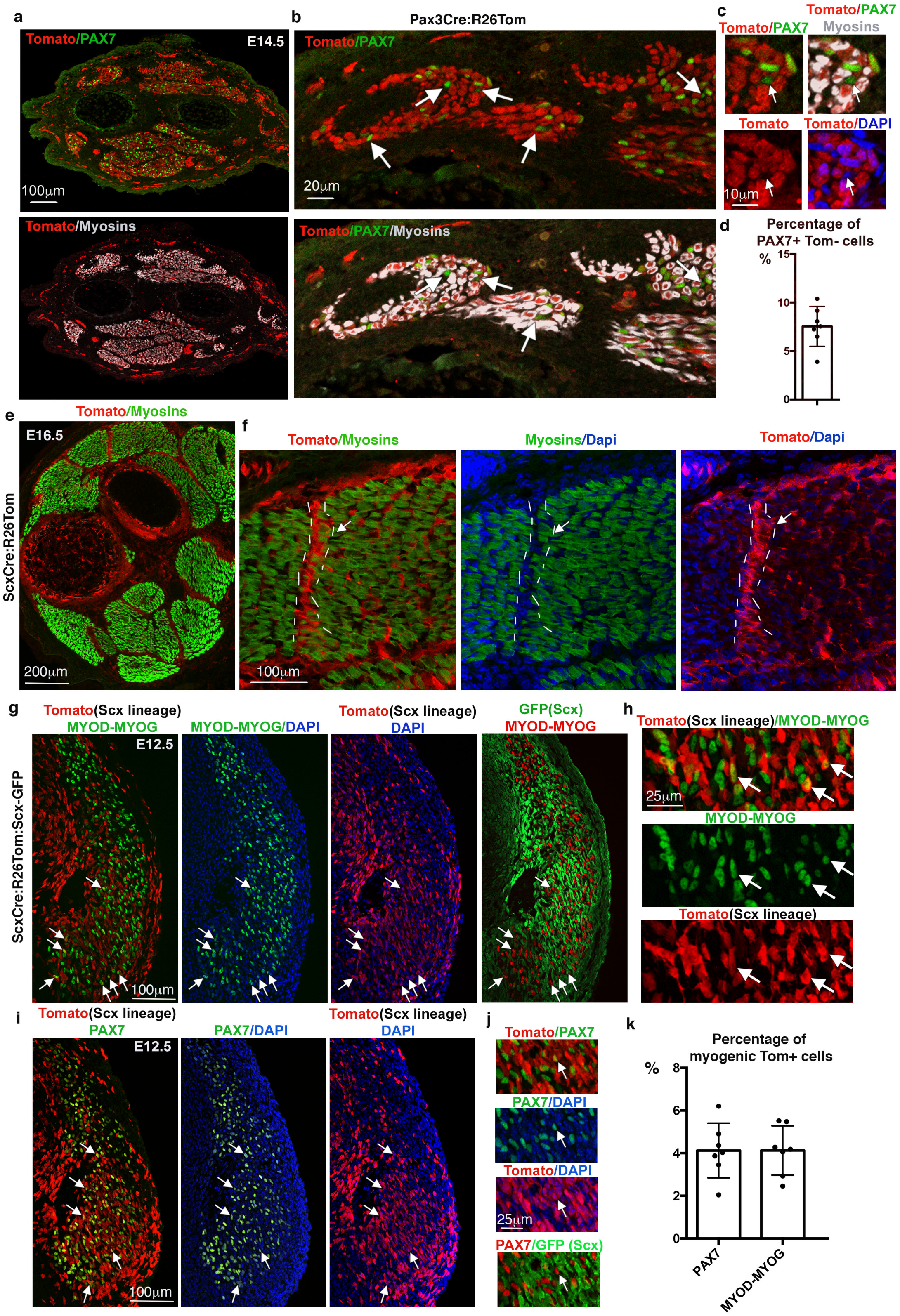

Supplementary Fig. 5

Supplementary Figure 5

A fraction of limb myogenic cells is not derived from *Pax3* lineage but from *Scx* lineage in mouse

(**a-c**) Transverse forelimb sections of E14.5 $Pax3^{Cre}$:$R26^{Tom}$ mice immunostained with Tomato (red), PAX7 (green) and MF20 (myosins, grey) antibodies, combined with DAPI staining. (b) Focus on dorsal muscles. (c) High magnifications on PAX7+/Tomato- cells. (b,c) Arrows point to PAX7+ cells that are Tomato-negative (*i.e* not Pax3 lineage-derived). (**d**) Percentage of PAX7+ cells that are not expressing Tomato versus total PAX7+ cells in limb muscles of E14.5 embryos. Data are presented as mean values +/- SD from 3 limbs.

(e,f) Transverse forelimb sections of E16.5 Scx^{Cre}:$R26^{Tom}$ mice immunostained with Tomato (red) and MF20 (myosins, green) antibodies, combined with DAPI (blue). (f) High magnifications of a Tomato+ junctional region between two dorsal limb muscles (encircled with dashed lines), showing the overlap (arrow) between myosins and Tomato labelling (Scx-lineage-derived cells). **(g-j)** Forelimb sections of E12.5 Scx^{Cre}:$R26^{Stop/Tom}$:*GFP(Scx)* mice immunostained with Tomato (Scx-lineage-derived cells, red) and MYOD/MYOG (green) (g,h) or PAX7 (green) (i,j) antibodies. (g,i) Arrows point to Tomato+ cells (*Scx*-derived) that are MYOD/MYOG+ (g) or PAX7+ (i), which are preferentially found at the periphery of muscle masses. (h,j) High magnifications showing MYOD/MYOG+ cells that are Tomato+ (*Scx* lineage) (h) or PAX7+ cells that are Tomato+ (*Scx* lineage) (j). (**k**) Percentage of Tomato+ (*Scx* lineage) cells within the PAX7+ or MYOD/MYOG+ populations in limb muscles of E16.5 embryos. Data are presented as mean values +/- SD from 3 limbs.

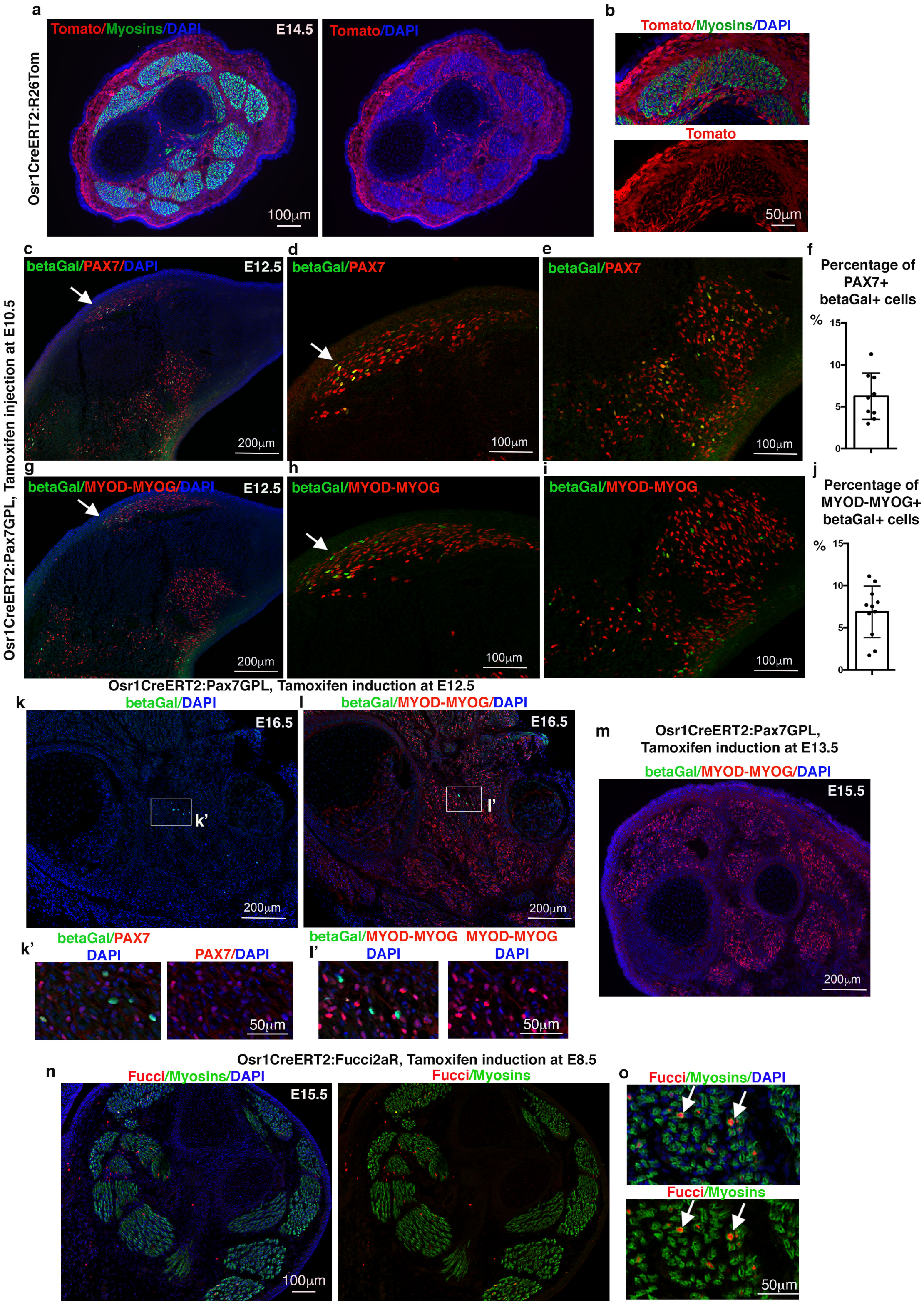

Supplementary Fig. 6

Supplementary Figure 6

A subpopulation of limb myogenic cells is derived from *Osr1* lineage in mouse

(a) Transverse forelimb sections of E14.5 *Osr1*^{CreERT2}*:R26Tom* mice immunostained with Tomato (red) and MF20 (myosins, green) antibodies, combined with DAPI staining (blue). (**b**) High magnification of dorsal limb muscles from (a). (**c-e, g-i)** Hindlimb sections of E12.5 *Osr1*^{CreERT2}*:Pax7*^{GPL} mice (tamoxifen injection at E10.5) immunostained with betaGal (*Osr1*-lineage-derived cells, green) and PAX7 (red) antibodies, combined with DAPI (blue) (c-e) or with betaGal (*Osr1*-lineage-derived cells, green) and MYOD/MYOG (red) antibodies, combined with DAPI (blue) (g-i). (c,d,g,h) Arrows point to betaGal+ cells at a border of the dorsal muscle mass. (d,e,h,i) are high magnifications of dorsal (d,h) and ventral (e,i) muscle masses of limbs shown in (c,g). (**f,j**) Percentage of betaGal+ cells (*Pax7* lineage in *Osr1* lineage) within the PAX7+ population (f) and percentage of betaGal+ cells (*Pax7* lineage in *Osr1* lineage) within the MYOD/MYOG+ population (j). (f,j) Data are presented as mean values +/- SD from 3 limbs. (**k,l,k',l'**) Hindlimb sections of E16.5 *Osr1*^{CreERT2}*:Pax7*^{GPL} mice (tamoxifen injection at E12.5) immunostained with betaGal (*Osr1*-lineage-derived cells, green) and PAX7 (red) antibodies, combined with DAPI (blue) (k,k') or immunostained with betaGal (*Osr1*-lineage-derived cells, green) and MYOD/MYOG (red) antibodies, combined with DAPI (blue) (l,l'). (**,k',l')** are high magnifications of limb regions squared in (k) and (l), respectively. (**m**) Hindlimb sections of E15.5 *Osr1*^{CreERT2}*:Pax7*^{GPL} mice (tamoxifen injection at E13.5) immunostained with betaGal (*Osr1*-lineage-derived cells, green) and MYOD/MYOG (red) antibodies, combined with DAPI (blue). (**n**) Transverse forelimb sections of E15.5 *Osr1*^{CreERT2}*:Fucci2aR* mice (tamoxifen injection at E8.5) immunostained with Fucci red (*Osr1*-lineage-derived cells, red) and MF20 (myosins, green) antibodies, combined with DAPI. (**o**) High magnification of limb muscle. Arrows point to Fucci+nuclei in myosin+.

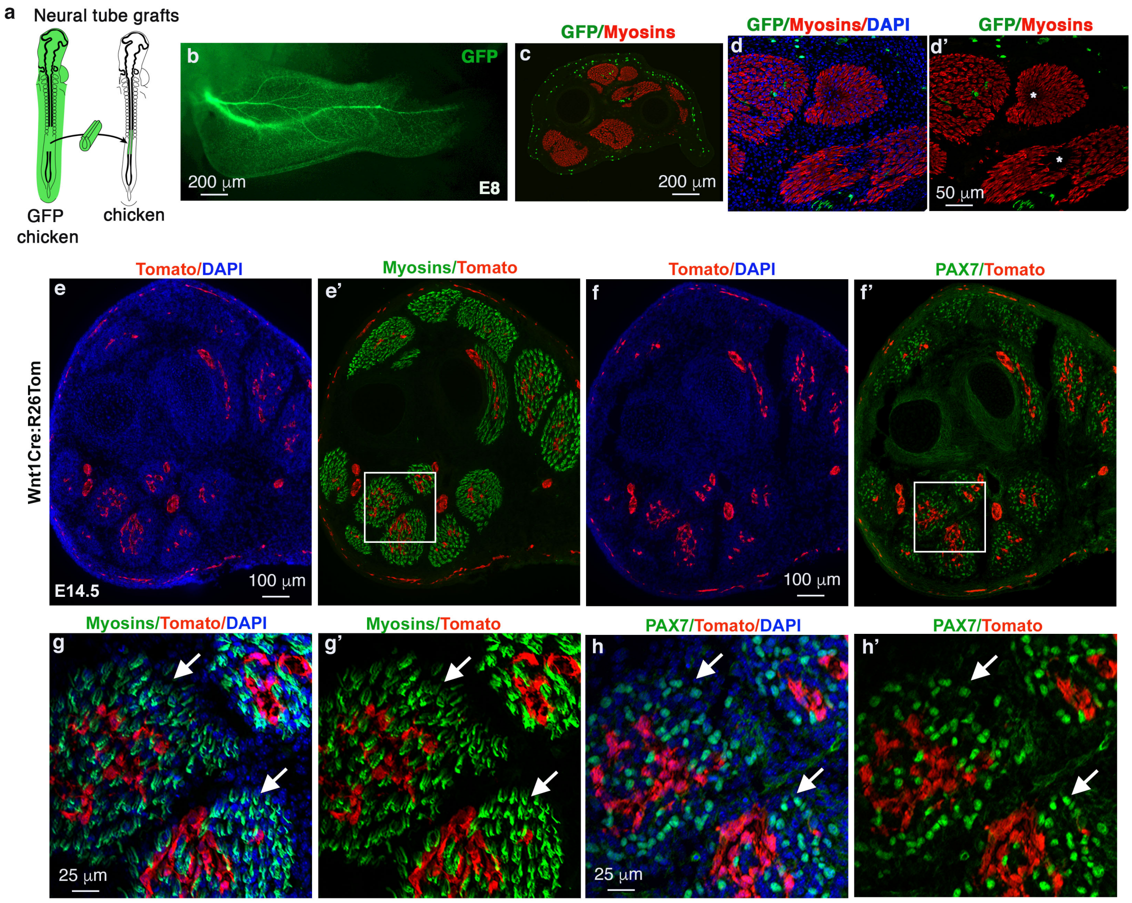

Supplementary Fig. 7

Supplementary Figure 7

Neural crest cells do not contribute to the myogenic lineage at muscle tips of limb skeletal muscles

(**a**) Diagram of isotopic/isochronic neural tube grafts from GFP chicken to chicken embryos. (**b**) Whole-mount of E8 grafted-forelimbs (6 days after grafting) showing GFP fluorescence in the neural tube cell derivatives. (**c**) Transverse forelimb sections from neural tube grafts showing GFP (neural tube-derived cells, green) fluorescence and MF20 (myosins, red) immunostaining. (**d**,**d'**) Focus on dorsal limb muscles with GFP fluorescence (green) and MF20 (myosins, red) antibodies, combined with DAPI (blue). White asterisks indicate the position of tendons. (**e,e'**) Transverse forelimb sections of E14.5 $Wnt1^{Cre}$:$R26^{Tom}$ mice immunostained with Tomato (Wnt1-lineage-derived cells, red) and MF20 (myosins, green) antibodies, combined with DAPI (blue). (**f,f'**) Transverse forelimb sections of E14.5 $Wnt1^{Cre}$:$R26^{Tom}$ mice immunostained with Tomato (Wnt1-lineage-derived cells, red) and PAX7 (green) antibodies, combined with DAPI (blue). (**g,g'**) and (**h,h'**) are high magnifications of ventral limb regions boxed in (e') and (f'), respectively. Arrows point to muscle cells, i.e myosin+ cells (green, g, g') or PAX7+ cells (green, h, h') at muscle tips displaying no Tomato labelling (Wnt1-lineage-derived cells, red).

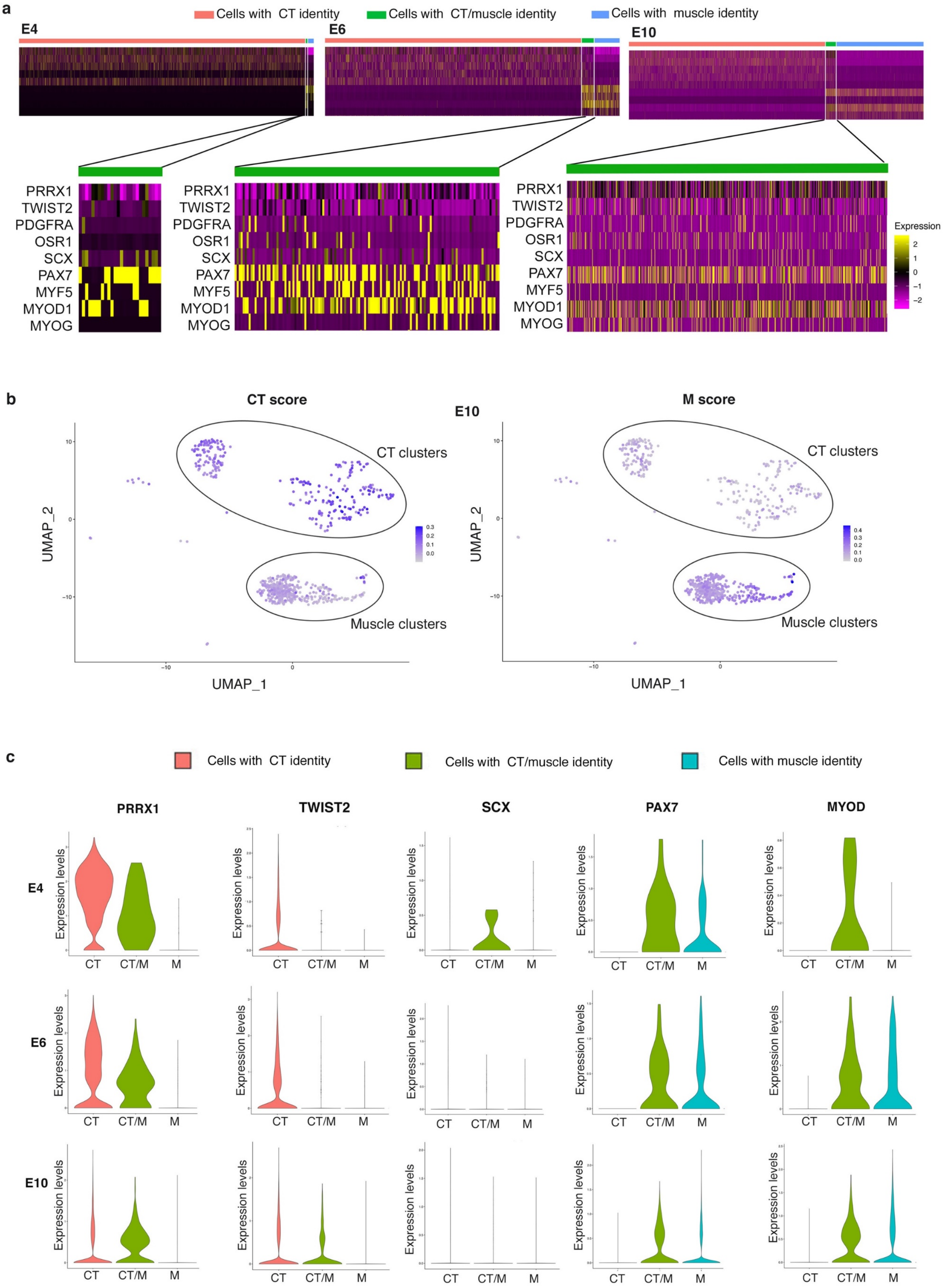

Supplementary Fig. 8

Supplementary Figure 8

Characterisation of the cells with a connective tissue/muscle (CT/M) identity

(**a**) Heatmaps showing the relative expression of five recognized CT markers (*PRRX1*, *TWIST2*, *PDFGRA*, *OSR1*, *SCX*) and four muscle markers (*PAX7*, *MYF5*, *MYOD*, *MYOG*) in cells grouped by their CT (red), CT/M (green) or M (blue) identity at E4, E6 and E10. Upregulated genes in yellow, downregulated genes in purple. High magnification of the heatmaps showing the relative expression of the same CT and muscle markers but only in CT/M cells at E4, E6 and E10. (**b**) Feature plots showing the distribution of CT and M scores (blue, high score; grey, low score) in cells with a CT/M identity at E10. **(c)** Violin plots showing Log-normalized expression levels of selected CT and muscle markers in cells grouped by their CT (red), CT/M (green) or M (blue) identity at E4, E6 and E10.

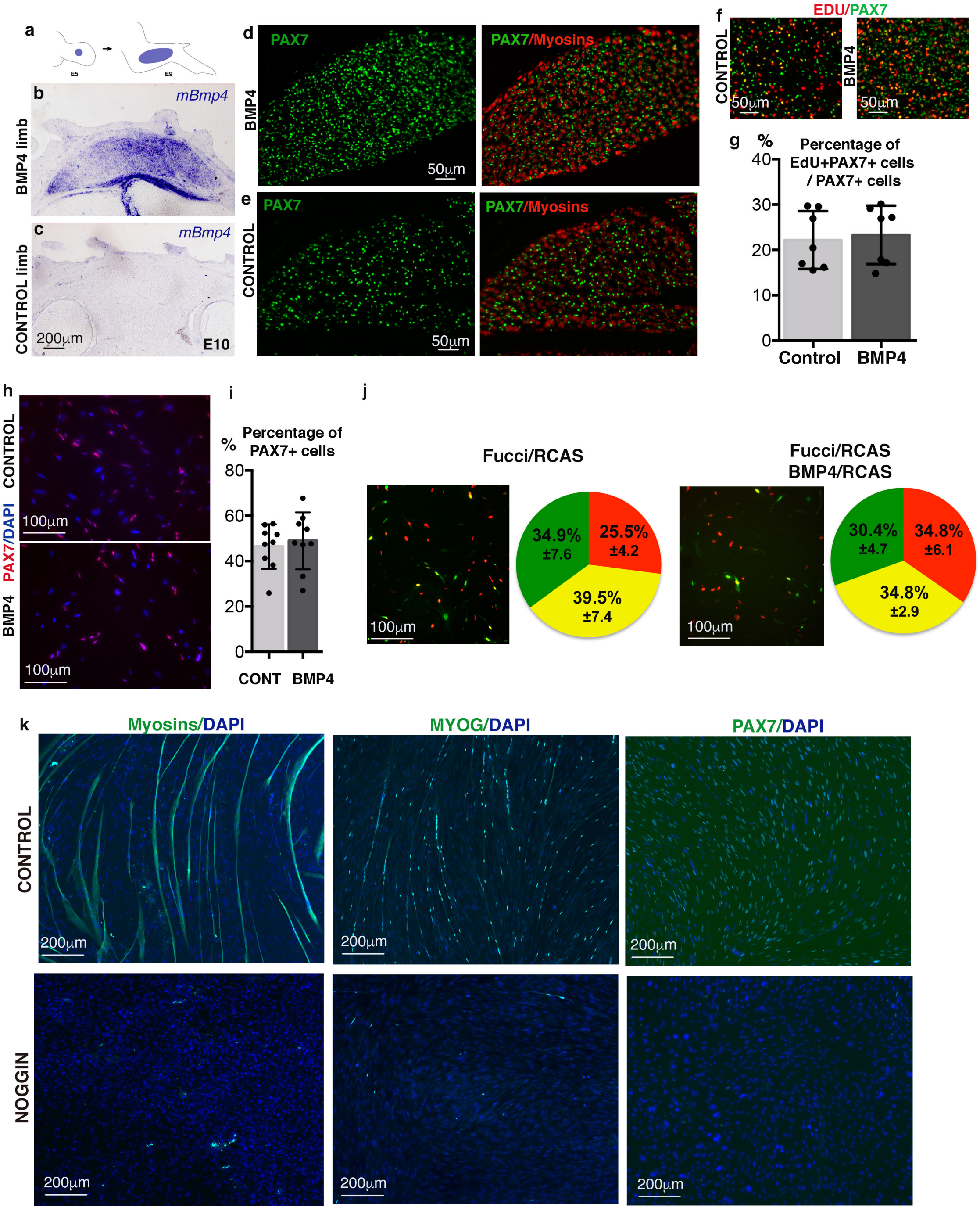

Supplementary Fig. 9

Supplementary Figure 9

BMP signalling promotes fibroblast/myoblast conversion

(**a**) Schematics of the grafting procedure of BMP4/RCAS-producing cells. (**b,c**) In situ hybridization with mBMP4 probe to transverse limb sections indicate the extent of ectopic *BMP4* in E10 grafted-embryos, 5 days after grafting. BMP4-grafted (b) and control (c) limbs are from the same embryo. (**d,e**) Immunostaining with PAX7 (green) and MF20 (myosins, red) antibodies to BMP4-grafted (d) and control (e) limb sections. (**f**) Representative views of PAX7+/EdU+ cells on sections of control and BMP4-grated limb muscles. (**g**) Percentage of PAX7+/EdU+ cells within the PAX7+ populations in control and BMP4-grafted limb muscles. Data are presented as mean values +/- SD from 3 limbs. **(h)** Immunostaining with PAX7 (red) antibody, counterstained with DAPI, on empty/RCAS- (control) or BMP4/RCAS-infected chicken primary myoblast cultures in proliferation conditions. **(i)** Percentage of PAX7+ cells within the culture in control and BMP4/RCAS-infected myoblast cultures. (i) Data are presented as mean values +/- SD from 3 limbs. **(j)** Cell cycle phase-indicative fluorescent signal (G1 phase, red; G1/S transition, yellow; S phase, green) from FUCCI/RCAS-infected cells in control and BMP4/RCAS-infected myoblast cultures. Quantitative representation of the proportion of cells in the different phases of the cell cycle (G1 phase, red; G1/S transition, yellow; S phase, green) for FUCCI/RCAS-infected cells in control and BMP4/RCAS-infected myoblast cultures. (**k**) Immunostaining with MF20 (myosins, green), MYOG and PAX7 antibodies (green), combined with DAPI (blue) on empty/RCAS- (control) and NOGGIN/RCAS-infected chicken primary myoblast cultures in differentiation conditions.

SUPPLEMENTARY Tables

Esteves de Lima et al.

Unexpected contribution of fibroblasts to muscle lineage as a mechanism for limb muscle patterning

Supplementary Table 1

Markers for the CT and muscle clusters at E4

List of CT and muscle markers associated to the CT and muscle clusters at E4.

In the “Cluster type” column, “CT” or “M” means that the cluster respectively has a CT or a M identity.

	ensembl_gene_id	external_gene_name	description	p_val	avg_logFC	pct.1	pct.2	p_val_adj	cluster	cluster type
41	ENSGALG00000003193	CRABP-I	cellular retinoic acid binding protein 1 [Source:NCBI gene;Acc:374211]	1.30079255230917e-205	0,582809107	0.993	0.877	3,17E-201	0	CT
206	ENSGALG00000015271	ENSGALG00000015271		2.04852463291731e-193	0,570657531	0.403	0.061	4,99E-189	0	CT
324	ENSGALG00000034661	ID1	inhibitor of DNA binding 1, HLH protein [Source:NCBI gene;Acc:395282]	1.16095263473479e-66	0,467402534	0.682	0.503	2,83E-62	0	CT
346	ENSGALG00000037164	ZFHX4	zinc finger homeobox 4 [Source:NCBI gene;Acc:395904]	9.36204334986323e-76	0,361193097	0.778	0.615	2,28E-71	0	CT
326	ENSGALG00000035016	ID2	inhibitor of DNA binding 2, HLH protein [Source:NCBI gene;Acc:395852]	2.23203005512518e-36	0,313438275	0.647	0.511	5,44E-32	0	CT
148	ENSGALG00000010461	EBF3	early B cell factor 3 [Source:NCBI gene;Acc:395175]	5.10099157603453e-36	0,305371884	0.594	0.438	1,24E-31	0	CT
208	ENSGALG00000015624	VCAN	versican [Source:NCBI gene;Acc:395565]	4.77637047918203e-46	0,277631602	0.762	0.648	1,16E-41	0	CT
9	ENSGALG00000000972	PBX3	PBX homeobox 3 [Source:NCBI gene;Acc:417093]	1.42909306752311e-63	0,275245835	0.463	0.257	3,48E-59	0	CT
59	ENSGALG00000005263	SOX8	SRY-box 8 [Source:NCBI gene;Acc:395483]	2.5904288320627e-81	0,252752347	0.311	0.097	6,31E-77	0	CT
126	ENSGALG00000009415	SMOC1	SPARC related modular calcium binding 1 [Source:NCBI gene;Acc:768688]	8.30211830144143e-198	0,689145919	0.552	0.154	2,02E-193	1	CT
219	ENSGALG00000016558	VEGFD	vascular endothelial growth factor D [Source:NCBI gene;Acc:395255]	1.05018842046666e-265	0,662015133	0.803	0.303	2,56E-261	1	CT
120	ENSGALG00000009274	HOXD12	homeobox D12 [Source:NCBI gene;Acc:396178]	5.78889658103247e-219	0,526305959	0.411	0.052	1,41E-214	1	CT
83	ENSGALG00000007000	NR2F2	nuclear receptor subfamily 2 group F member 2 [Source:NCBI gene;Acc:386585]	1.61068944913826e-139	0,51394566	0.863	0.547	3,92E-135	1	CT
266	ENSGALG00000028983	HOXA9	homeobox A9 [Source:NCBI gene;Acc:396096]	8.64012495365616e-129	0,406652671	0.739	0.358	2,10E-124	1	CT
371	ENSGALG00000039629	HOXD11	homeobox D11 [Source:NCBI gene;Acc:395328]	1.9275542356211e-198	0,389499621	0.417	0.064	4,69E-194	1	CT
321	ENSGALG00000034346	RDH10	retinol dehydrogenase 10 (all-trans) [Source:NCBI gene;Acc:420183]	4.47352953383661e-76	0,377977213	0.303	0.098	1,09E-71	1	CT
110	ENSGALG00000008677	VIM	vimentin [Source:NCBI gene;Acc:420519]	4.9854635237629e-08	0,372313198	0.961	0.952	0,001214259	1	CT
123	ENSGALG00000009302	EMX2	empty spiracles homeobox 2 [Source:NCBI gene;Acc:423923]	2.79129509437426e-104	0,34123489	0.38	0.111	6,80E-100	1	CT
136	ENSGALG00000009654	mylk1	myeloid leukemia factor 1 [Source:NCBI gene;Acc:425019]	5.56461050876704e-93	0,322095807	0.554	0.263	1,36E-88	1	CT
245	ENSGALG00000026313	RND3	Rho family GTPase 3 [Source:NCBI gene;Acc:424312]	1.08996072787699e-70	0,297792104	0.562	0.298	2,65E-66	1	CT
215	ENSGALG00000016348	SAT1	spermidine/spermine N1-acetyltransferase 1 [Source:NCBI gene;Acc:374006]	5.6242248610896e-39	0,279795021	0.475	0.294	1,37E-34	1	CT
75	ENSGALG00000006609	EPHB1	EPH receptor B1 [Source:NCBI gene;Acc:396177]	1.76347109195774e-79	0,271929547	0.542	0.26	4,30E-75	1	CT
159	ENSGALG00000011469	IGFBP2	insulin like growth factor binding protein 2 [Source:NCBI gene;Acc:396315]	4.14980204053981e-60	0,267040968	0.831	0.588	1,01E-55	1	CT
209	ENSGALG00000015627	HAPLN1	hyaluronan and proteoglycan link protein 1 [Source:NCBI gene;Acc:396475]	1.00553332451017e-34	0,26196464	0.521	0.346	2,45E-30	1	CT
128	ENSGALG00000009476	CDK6	cyclin dependent kinase 6 [Source:NCBI gene;Acc:420558]	2.36637304310344e-56	0,260502755	0.814	0.579	5,76E-52	1	CT
191	ENSGALG00000014261	UCHL1	ubiquitin C-terminal hydrolase L1 [Source:NCBI gene;Acc:770302]	8.38181012957136e-83	0,252829481	0.392	0.146	2,04E-78	1	CT
286	ENSGALG00000031487	FSTL4	follistatin like 4 [Source:NCBI gene;Acc:395163]	5.67198685072042e-105	0,250369902	0.283	0.058	1,38E-100	1	CT
154	ENSGALG00000011271	LUM	lumican [Source:NCBI gene;Acc:417891]	4.31061958230172e-233	0,82500982	0.825	0.36	1,05E-228	2	CT
155	ENSGALG00000011274	DCN	decorin [Source:NCBI gene;Acc:417892]	1.98016947997077e-205	0,773975482	0.761	0.327	4,82E-201	2	CT
377	ENSGALG00000040993	BAMBI	BMP and activin membrane bound inhibitor [Source:NCBI gene;Acc:428413]	7.03961007603618e-186	0,729780169	0.573	0.174	1,71E-181	2	CT
197	ENSGALG00000014873	GJA1	gap junction protein alpha 1 [Source:NCBI gene;Acc:395278]	1.17735681147058e-175	0,723521356	0.967	0.806	2,87E-171	2	CT
376	ENSGALG00000040493	PTN	pleiotrophin [Source:NCBI gene;Acc:418125]	2.43975864503084e-126	0,660126323	0.959	0.67	5,94E-122	2	CT
177	ENSGALG00000012834	AKR1D1	aldo-keto reductase family 1 member D1 [Source:NCBI gene;Acc:418107]	4.6433287717753e-114	0,654772617	0.729	0.368	1,13E-109	2	CT
207	ENSGALG00000015348	ALCAM	activated leukocyte cell adhesion molecule [Source:NCBI gene;Acc:396092]	3.50736999958206e-190	0,647155565	0.487	0.102	8,54E-186	2	CT
84	ENSGALG00000007114	APOA1	apolipoprotein A1 [Source:NCBI gene;Acc:396536]	1.4696865061209e-215	0,599518651	0.28	0.008	3,58E-211	2	CT
385	ENSGALG00000042492	PITX2	paired like homeodomain 2 [Source:NCBI gene;Acc:395862]	4.11321684804829e-211	0,577561957	0.584	0.13	1,00E-206	2	CT
52	ENSGALG00000004274	TWIST2	twist family bHLH transcription factor 2 [Source:NCBI gene;Acc:395405]	2.03330334497046e-132	0,570687592	0.578	0.226	4,95E-128	2	CT
169	ENSGALG00000011962	NDNF	neuron-derived neurotrophic factor [Source:NCBI gene;Acc:422672]	5.71719284228785e-159	0,565871123	0.46	0.114	1,39E-154	2	CT
61	ENSGALG00000005352	GATA5	GATA binding protein 5 [Source:NCBI gene;Acc:396391]	1.14900484603673e-234	0,548013585	0.369	0.026	2,80E-230	2	CT
147	ENSGALG00000010461	EBF3	early B cell factor 3 [Source:NCBI gene;Acc:395175]	1.74392765722576e-100	0,469020907	0.807	0.431	4,25E-96	2	CT
134	ENSGALG00000009612	TGFB2	transforming growth factor beta 2 [Source:NCBI gene;Acc:421352]	4.89431498763213e-134	0,457019465	0.355	0.07	1,19E-129	2	CT
380	ENSGALG00000041555	COL1A1	collagen type I alpha 1 chain [Source:NCBI gene;Acc:395532]	7.09554777506815e-90	0,440857636	0.978	0.934	1,73E-85	2	CT
361	ENSGALG00000038848	MSX2	msh homeobox 2 [Source:NCBI gene;Acc:395245]	4.38081352099685e-77	0,427864759	0.552	0.256	1,07E-72	2	CT
353	ENSGALG00000037811	NRSN1	neurensin 1 [Source:NCBI gene;Acc:420822]	1.05543806032551e-95	0,410579444	0.467	0.176	2,57E-91	2	CT
263	ENSGALG00000028685	SEMA5A	semaphorin 5A [Source:NCBI gene;Acc:420931]	2.14172510364276e-56	0,358480071	0.374	0.166	5,22E-52	2	CT

73	ENSGALG00000006560	SEMA3A	semaphorin 3A [Source:NCBI gene;Acc:395825]	2.42223241413583e-117	0,348126816	0.377	0.092	5,90E-113	2	CT
104	ENSGALG00000008253	TBX5	T-box 5 [Source:NCBI gene;Acc:373988]	1.80702520860721e-58	0,337985847	0.645	0.427	4,40E-54	2	CT
223	ENSGALG00000017169	ELMOD1	ELMO domain containing 1 [Source:NCBI gene;Acc:418971]	2.80658747903579e-106	0,328024804	0.349	0.086	6,84E-102	2	CT
383	ENSGALG00000042389	EN1	engrailed homeobox 1 [Source:NCBI gene;Acc:771008]	3.75170838507685e-190	0,322242632	0.246	0.006	9,14E-186	2	CT
239	ENSGALG00000025898	SMAD6	SMAD family member 6 [Source:NCBI gene;Acc:374096]	3.03981731554059e-55	0,318082327	0.433	0.216	7,40E-51	2	CT
127	ENSGALG00000009476	CDK6	cyclin dependent kinase 6 [Source:NCBI gene;Acc:420558]	4.2744968051164e-41	0,301705712	0.764	0.622	1,04E-36	2	CT
131	ENSGALG00000009532	GXYLT1	glucoside xylosyltransferase 1 [Source:NCBI gene;Acc:417789]	5.88025368435876e-41	0,297884442	0.644	0.477	1,43E-36	2	CT
329	ENSGALG00000035584	COL3A1	collagen type III alpha 1 chain [Source:NCBI gene;Acc:396340]	2.2912608576763e-79	0,29788353	0.362	0.117	5,58E-75	2	CT
165	ENSGALG00000011687	AHNAK2	AHNAK nucleoprotein 2 [Source:NCBI gene;Acc:100859120]	5.47837807848535e-35	0,293602137	0.18	0.059	1,33E-30	2	CT
32	ENSGALG00000002515	OLFM1	olfactomedin 1 [Source:NCBI gene;Acc:395535]	7.24198152536787e-42	0,29223455	0.45	0.263	1,76E-37	2	CT
106	ENSGALG00000008367	MDK	midkine (neurite growth-promoting factor 2) [Source:NCBI gene;Acc:423196]	6.67768504990406e-74	0,288813954	1	0.99	1,63E-69	2	CT
238	ENSGALG00000025738	RHOU	ras homolog family member U [Source:NCBI gene;Acc:428589]	3.46708626235803e-38	0,28818728	0.438	0.266	8,44E-34	2	CT
339	ENSGALG00000036240	AKAP12	A-kinase anchoring protein 12 [Source:NCBI gene;Acc:421634]	1.4185331676162e-41	0,286257707	0.901	0.843	3,45E-37	2	CT
17	ENSGALG00000001768	TENM2	teneurin transmembrane protein 2 [Source:NCBI gene;Acc:373854]	8.8163774874267e-110	0,28492922	0.299	0.056	2,15E-105	2	CT
315	ENSGALG00000034048	BCL11A	B-cell CLL/lymphoma 11A [Source:NCBI gene;Acc:421199]	3.06341064574651e-33	0,281692545	0.711	0.568	7,46E-29	2	CT
273	ENSGALG00000029429	TGFBI	transforming growth factor beta induced [Source:NCBI gene;Acc:395897]	1.28088144908631e-27	0,275494124	0.474	0.321	3,12E-23	2	CT
260	ENSGALG00000027891	NREP	neuronal regeneration related protein [Source:NCBI gene;Acc:396353]	7.15693291880901e-28	0,274272419	0.606	0.48	1,74E-23	2	CT
162	ENSGALG00000011541	BIN1	bridging integrator 1 [Source:NCBI gene;Acc:424228]	2.17195461602067e-37	0,265112273	0.386	0.215	5,29E-33	2	CT
202	ENSGALG00000015013	MSX1	msh homeobox 1 [Source:NCBI gene;Acc:396484]	1.38285408925657e-141	1,07138207	0.792	0.147	3,37E-137	3	CT
170	ENSGALG00000012017	DAAM1	dishevelled associated activator of morphogenesis 1 [Source:NCBI gene;Acc:423532]	1.17004426017942e-64	0,724451444	0.853	0.373	2,85E-60	3	CT
366	ENSGALG00000039238	SALL1	spalt like transcription factor 1 [Source:NCBI gene;Acc:395446]	0	0,701674108	0.756	0.039	0	3	CT
374	ENSGALG00000040021	HOXA11	homeobox A11 [Source:NCBI gene;Acc:395327]	1.17410885745214e-268	0,674842568	0.772	0.058	2,86E-264	3	CT
175	ENSGALG00000012775	TFAP2A	transcription factor AP-2 alpha [Source:NCBI gene;Acc:395982]	0	0,621738976	0.695	0.014	0	3	CT
300	ENSGALG00000032687	PHLDA2	pleckstrin homology like domain family A member 2 [Source:NCBI gene;Acc:423088]	5.69214383435849e-107	0,553409622	0.685	0.124	1,39E-102	3	CT
198	ENSGALG00000014873	GJA1	gap junction protein alpha 1 [Source:NCBI gene;Acc:395278]	5.15884996634253e-45	0,534090686	0.985	0.831	1,26E-40	3	CT
310	ENSGALG00000033212	HSP90AA1	heat shock protein 90 alpha family class A member 1 [Source:NCBI gene;Acc:423463]	1.65677757698847e-48	0,53071838	1	0.93	4,04E-44	3	CT
267	ENSGALG00000028983	HOXA9	homeobox A9 [Source:NCBI gene;Acc:396096]	5.10082513957496e-48	0,529840439	0.934	0.453	1,24E-43	3	CT
317	ENSGALG00000034168	WNT5A	Wnt family member 5A [Source:NCBI gene;Acc:395703]	5.80695380547444e-67	0,527849095	0.766	0.244	1,41E-62	3	CT
279	ENSGALG00000030550	ATP2B1	ATPase plasma membrane Ca2+ transporting 1 [Source:NCBI gene;Acc:374244]	2.275425907231e-60	0,525506659	0.797	0.287	5,54E-56	3	CT
28	ENSGALG00000002203	FGF18	fibroblast growth factor 18 [Source:NCBI gene;Acc:395453]	0	0,49432029	0.563	0.014	0	3	CT
295	ENSGALG00000032329	NPM3	nucleophosmin/nucleoplasmin 3 [Source:NCBI gene;Acc:770430]	5.41793479614672e-86	0,493417586	0.761	0.185	1,32E-81	3	CT
72	ENSGALG00000006512	HSPA8	heat shock 70kDa protein 8 [Source:NCBI gene;Acc:395853]	1.0752849658488e-45	0,492717623	1	0.921	2,62E-41	3	CT
173	ENSGALG00000012732	PHACTR1	phosphatase and actin regulator 1 [Source:NCBI gene;Acc:395093]	9.39646160192811e-201	0,48271862	0.558	0.039	2,29E-196	3	CT
96	ENSGALG00000007936	PGK2	phosphoglycerate kinase 2 [Source:NCBI gene;Acc:395833]	1.82935760601512e-40	0,47998493	0.97	0.704	4,46E-36	3	CT
63	ENSGALG00000005499	IL17RD	interleukin 17 receptor D [Source:NCBI gene;Acc:395186]	1.08513159379338e-198	0,478823179	0.528	0.034	2,64E-194	3	CT
116	ENSGALG00000009070	HSPE1	heat shock protein family E (Hsp10) member 1 [Source:NCBI gene;Acc:395948]	5.56769893228706e-37	0,44313088	0.995	0.864	1,36E-32	3	CT
27	ENSGALG00000002197	NPM1	nucleophosmin [Source:NCBI gene;Acc:396203]	1.41264042619514e-68	0,438698476	1	0.999	3,44E-64	3	CT
152	ENSGALG00000011207	DUSP6	dual specificity phosphatase 6 [Source:NCBI gene;Acc:374272]	2.50291679511484e-128	0,430275464	0.655	0.092	6,10E-124	3	CT
38	ENSGALG00000002988	PHGDH	phosphoglycerate dehydrogenase [Source:NCBI gene;Acc:424381]	1.04220596824931e-90	0,4297425	0.635	0.124	2,54E-86	3	CT
241	ENSGALG00000026077	ALDOC	aldolase, fructose-bisphosphate C [Source:NCBI gene;Acc:395492]	3.12298343394947e-29	0,426193653	0.919	0.647	7,61E-25	3	CT
304	ENSGALG00000032930	NCL	nucleolin [Source:NCBI gene;Acc:396201]	1.41354245307213e-56	0,421168609	1	0.991	3,44E-52	3	CT
217	ENSGALG00000016462	MYCN	v-myc avian myelocytomatosis viral oncogene neuroblastoma derived homolog [Source:NCBI gene;	1.74348257358474e-76	0,40113957	0.599	0.126	4,25E-72	3	CT
163	ENSGALG00000011581	KIF26A	kinesin family member 26A [Source:NCBI gene;Acc:423489]	1.07156897634272e-76	0,389631834	0.538	0.1	2,61E-72	3	CT
24	ENSGALG00000002107	RANBP1	RAN binding protein 1 [Source:NCBI gene;Acc:416787]	3.52108412611971e-26	0,380978613	0.954	0.733	8,58E-22	3	CT
184	ENSGALG00000013342	GBX2	gastrulation brain homeobox 2 [Source:NCBI gene;Acc:395950]	7.47926212550901e-178	0,370189999	0.447	0.026	1,82E-173	3	CT
101	ENSGALG00000008094	HSPD1	heat shock protein family D (Hsp60) member 1 [Source:NCBI gene;Acc:424059]	9.34752384645065e-29	0,365359472	0.995	0.878	2,28E-24	3	CT
124	ENSGALG00000009309	EIF5A2	eukaryotic translation initiation factor 5A [Source:NCBI gene;Acc:396545]	9.93968359903727e-37	0,36484471	1	0.931	2,42E-32	3	CT
60	ENSGALG00000005309	TKT	transketolase like 1 [Source:NCBI gene;Acc:415991]	2.82282077397277e-25	0,358635846	0.924	0.595	6,88E-21	3	CT
122	ENSGALG00000009286	ATP5G3	ATP synthase, H+ transporting, mitochondrial Fo complex subunit C3 (subunit 9) [Source:NCBI gene	1.4782793111098e-32	0,356971646	1	0.974	3,60E-28	3	CT
168	ENSGALG00000011931	SNU13	small nuclear ribonucleoprotein 13 [Source:HGNC Symbol;Acc:HGNC:7819]	1.17439387802392e-28	0,354140094	0.99	0.794	2,86E-24	3	CT
20	ENSGALG00000001992	PKLR	pyruvate kinase, liver and RBC [Source:NCBI gene;Acc:396456]	4.8913903781804e-25	0,350613254	0.959	0.675	1,19E-20	3	CT
34	ENSGALG00000002569	RAN	RAN, member RAS oncogene family [Source:NCBI gene;Acc:396193]	1.21436787761206e-24	0,345082854	0.995	0.884	2,96E-20	3	CT
16	ENSGALG00000001717	SLC16A1	solute carrier family 16 member 1 [Source:NCBI gene;Acc:419875]	2.7560387051056e-32	0,341787249	0.797	0.366	6,71E-28	3	CT

141	ENSGALG00000009975	CCT2	chaperonin containing TCP1 subunit 2 [Source:NCBI gene;Acc:417846]	1.18654845450235e-22	0,340337595	0.97	0.811	2,89E-18	3	CT
179	ENSGALG00000013041	CCT5	chaperonin containing TCP1 subunit 5 [Source:NCBI gene;Acc:420930]	1.28636400658903e-25	0,338253439	0.98	0.802	3,13E-21	3	CT
137	ENSGALG00000009677	SSB	Sjogren syndrome antigen B [Source:NCBI gene;Acc:395268]	4.05147716417622e-24	0,333973384	0.909	0.582	9,87E-20	3	CT
319	ENSGALG00000034218	PA2G4	proliferation-associated 2G4, 38kDa [Source:NCBI gene;Acc:425279]	1.24912363766249e-21	0,32114676	0.929	0.667	3,04E-17	3	CT
121	ENSGALG00000009274	HOXD12	homeobox D12 [Source:NCBI gene;Acc:396178]	5.07809213656388e-49	0,31710987	0.558	0.143	1,24E-44	3	CT
196	ENSGALG00000014872	FGF10	fibroblast growth factor 10 [Source:NCBI gene;Acc:395432]	1.20859983330049e-65	0,314011331	0.528	0.108	2,94E-61	3	CT
80	ENSGALG00000006974	DDX27	DEAD-box helicase 27 [Source:NCBI gene;Acc:419308]	1.86719576602313e-20	0,312987133	0.802	0.47	4,55E-16	3	CT
292	ENSGALG00000032316	CDV3	CDV3 homolog [Source:NCBI gene;Acc:420693]	7.84487717090786e-23	0,312100221	0.97	0.689	1,91E-18	3	CT
336	ENSGALG00000035836	LDHB	lactate dehydrogenase B [Source:NCBI gene;Acc:373997]	3.14898763300383e-28	0,310217665	0.995	0.958	7,67E-24	3	CT
210	ENSGALG00000015821	CCT8	chaperonin containing TCP1 subunit 8 [Source:NCBI gene;Acc:418486]	7.89266578383438e-20	0,307394879	0.904	0.699	1,92E-15	3	CT
370	ENSGALG00000039629	HOXD11	homeobox D11 [Source:NCBI gene;Acc:395328]	6.85426404828959e-48	0,304282925	0.574	0.154	1,67E-43	3	CT
363	ENSGALG00000038933	FABP7	fatty acid binding protein 7 [Source:NCBI gene;Acc:396246]	5.59488380735364e-22	0,295101575	0.614	0.295	1,36E-17	3	CT
301	ENSGALG00000032730	NOP56	NOP56 ribonucleoprotein [Source:NCBI gene;Acc:426574]	3.39308431780339e-19	0,294019368	0.964	0.759	8,26E-15	3	CT
203	ENSGALG00000015015	CYTL1	cytokine like 1 [Source:NCBI gene;Acc:422849]	1.66757715662218e-87	0,288419903	0.447	0.059	4,06E-83	3	CT
309	ENSGALG00000033196	EIF2S2	eukaryotic translation initiation factor 2 subunit beta [Source:NCBI gene;Acc:395295]	1.15240294636569e-17	0,286295505	0.949	0.735	2,81E-13	3	CT
40	ENSGALG00000003011	TOM1L1	target of myb1 like 1 membrane trafficking protein [Source:NCBI gene;Acc:417391]	3.65209924219328e-83	0,285781279	0.391	0.049	8,90E-79	3	CT
99	ENSGALG00000008072	CHRDL1	chordin like 1 [Source:NCBI gene;Acc:373985]	2.21330533516047e-109	0,285222145	0.365	0.03	5,39E-105	3	CT
253	ENSGALG00000026989	HOXA7	homeobox A7 [Source:NCBI gene;Acc:395290]	7.62135723714191e-23	0,283933087	0.706	0.342	1,86E-18	3	CT
91	ENSGALG00000007606	PGAM1	phosphoglycerate mutase 1 [Source:NCBI gene;Acc:428969]	1.70299406150062e-18	0,279865723	0.949	0.724	4,15E-14	3	CT
29	ENSGALG00000002242	GALNT9	polypeptide N-acetylgalactosaminyltransferase 9 [Source:NCBI gene;Acc:416796]	2.80718530050651e-137	0,277591678	0.289	0.013	6,84E-133	3	CT
79	ENSGALG00000006939	PFKP	phosphofructokinase, platelet [Source:NCBI gene;Acc:428411]	5.89894395323896e-30	0,274113116	0.66	0.253	1,44E-25	3	CT
352	ENSGALG00000037807	NOP2	NOP2 nucleolar protein [Source:NCBI gene;Acc:418277]	5.57388083864274e-21	0,273070837	0.761	0.392	1,36E-16	3	CT
248	ENSGALG00000026631	HOXA10	homeobox A10 [Source:NCBI gene;Acc:776143]	1.09626630934652e-70	0,26860329	0.447	0.073	2,67E-66	3	CT
109	ENSGALG00000008517	MCTS1	MCTS1, re-initiation and release factor [Source:NCBI gene;Acc:422363]	6.4728288256481e-16	0,2677979	0.838	0.536	1,58E-11	3	CT
13	ENSGALG00000001613	GNL3	G protein nucleolar 3 [Source:NCBI gene;Acc:415897]	1.67352849234191e-16	0,267761326	0.817	0.539	4,08E-12	3	CT
291	ENSGALG00000032039	HOXD13	homeobox D13 [Source:NCBI gene;Acc:396415]	5.65218414403509e-56	0,265703346	0.33	0.052	1,38E-51	3	CT
39	ENSGALG00000002990	PRELID1	PRELI domain containing 1 [Source:NCBI gene;Acc:395819]	1.99736508518078e-14	0,260073003	0.919	0.685	4,86E-10	3	CT
410	ENSGALG00000053063	MRPL40	mitochondrial ribosomal protein L40 [Source:NCBI gene;Acc:100858795]	7.95311832569174e-17	0,257267052	0.883	0.602	1,94E-12	3	CT
8	ENSGALG00000000884	CXXC5	CXXC finger protein 5 [Source:NCBI gene;Acc:416138]	2.10027533836196e-19	0,256497675	0.736	0.384	5,12E-15	3	CT
270	ENSGALG00000029304	SMARCD2	SWI/SNF related, matrix associated, actin dependent regulator of chromatin, subfamily d, member	4.11409283924524e-20	0,255944675	0.619	0.292	1,00E-15	3	CT
333	ENSGALG00000035679	LMO1	LIM domain only 1 (rhombotin 1) [Source:NCBI gene;Acc:100858792]	3.39994869615553e-165	0,254560141	0.365	0.017	8,28E-161	3	CT
57	ENSGALG00000004956	GPI	glucose-6-phosphate isomerase [Source:NCBI gene;Acc:415783]	9.10829075435376e-15	0,254546115	0.756	0.458	2,22E-10	3	CT
199	ENSGALG00000015000	LYAR	Ly1 antibody reactive [Source:NCBI gene;Acc:422846]	1.7200706072894e-21	0,25262541	0.695	0.334	4,19E-17	3	CT
388	ENSGALG00000042871	SLIT1	slit guidance ligand 1 [Source:NCBI gene;Acc:395293]	2.41866327285345e-124	1,352443626	0.933	0.151	5,89E-120	4	M
272	ENSGALG00000029401	SIX1	SIX homeobox 1 [Source:NCBI gene;Acc:693262]	1.64206584615612e-84	1,055063183	0.885	0.197	4,00E-80	4	M
243	ENSGALG00000026276	TCF15	transcription factor 15 (basic helix-loop-helix) [Source:NCBI gene;Acc:395788]	2.54447238723269e-219	0,961888361	0.769	0.045	6,20E-215	4	M
98	ENSGALG00000008072	CHRDL1	chordin like 1 [Source:NCBI gene;Acc:373985]	6.9088158520878e-233	0,93757273	0.683	0.03	1,68E-228	4	M
161	ENSGALG00000011511	CKB	creatine kinase B [Source:NCBI gene;Acc:396248]	9.18940371754323e-58	0,914861831	0.587	0.107	2,24E-53	4	M
373	ENSGALG00000040021	HOXA11	homeobox A11 [Source:NCBI gene;Acc:395327]	6.64168243139158e-119	0,894013589	0.692	0.073	1,62E-114	4	M
46	ENSGALG00000003642	PDGFA	platelet derived growth factor subunit A [Source:NCBI gene;Acc:374196]	3.61849986606133e-281	0,821241845	0.712	0.024	8,81E-277	4	M
296	ENSGALG00000032329	NPM3	nucleophosmin/nucleoplasmin 3 [Source:NCBI gene;Acc:770430]	2.35930668382762e-54	0,814946786	0.731	0.197	5,75E-50	4	M
359	ENSGALG00000038515	LFNG	LFNG O-fucosylpeptide 3-beta-N-acetylglucosaminyltransferase [Source:NCBI gene;Acc:395790]	4.79993448865209e-169	0,691232629	0.615	0.035	1,17E-164	4	M
67	ENSGALG00000006252	LMO4	LIM domain only 4 [Source:NCBI gene;Acc:373901]	5.59700927175259e-15	0,625441257	0.827	0.549	1,36E-10	4	M
381	ENSGALG00000041826	S100A6	S100 calcium binding protein A6 [Source:NCBI gene;Acc:373951]	2.6435277217609e-74	0,593593209	0.615	0.088	6,44E-70	4	M
133	ENSGALG00000009612	TGFB2	transforming growth factor beta 2 [Source:NCBI gene;Acc:421352]	3.70712814761865e-35	0,574514739	0.519	0.117	9,03E-31	4	M
204	ENSGALG00000015132	CDH2	cadherin 2 [Source:NCBI gene;Acc:414745]	1.56147449144146e-32	0,533493771	0.74	0.24	3,80E-28	4	M
311	ENSGALG00000033631	MYC	v-myc avian myelocytomatosis viral oncogene homolog [Source:NCBI gene;Acc:420332]	2.43864043444633e-120	0,526760409	0.529	0.038	5,94E-116	4	M
51	ENSGALG00000003999	NAV2	neuron navigator 2 [Source:NCBI gene;Acc:422977]	9.69233913364295e-62	0,494653834	0.519	0.074	2,36E-57	4	M
416	ENSGALG00000054783	NDRG1	N-myc downstream regulated 1 [Source:NCBI gene;Acc:420321]	6.03499821245724e-11	0,465008889	0.202	0.055	1,47E-06	4	M
372	ENSGALG00000039990	TNC	tenascin C [Source:NCBI gene;Acc:396440]	5.13378871531828e-182	0,454262042	0.24	0.002	1,25E-177	4	M
249	ENSGALG00000026631	HOXA10	homeobox A10 [Source:NCBI gene;Acc:776143]	1.02323240058298e-57	0,444821077	0.519	0.079	2,49E-53	4	M
182	ENSGALG00000013239	ENSGALG00000013239		9.02036383118796e-18	0,427201089	0.587	0.228	2,20E-13	4	M
293	ENSGALG00000032316	CDV3	CDV3 homolog [Source:NCBI gene;Acc:420693]	8.82089461544056e-18	0,424255422	0.923	0.696	2,15E-13	4	M

224	ENSGALG00000017179	PDGFD	platelet derived growth factor D [Source:NCBI gene;Acc:418978]	7.75730127824134e-32	0,423399452	0.298	0.046	1,89E-27	4	M
261	ENSGALG00000028543	FGFR4	fibroblast growth factor receptor 4 [Source:NCBI gene;Acc:395603]	4.70288218416841e-57	0,423187377	0.49	0.072	1,15E-52	4	M
394	ENSGALG00000043204	PAX7	paired box 7 [Source:NCBI gene;Acc:395942]	0	0,411239996	0.375	0.001	0	4	M
289	ENSGALG00000031593	TMSB15B	thymosin beta 15B [Source:NCBI gene;Acc:100502566]	4.33669062583602e-19	0,399591501	0.99	0.909	1,06E-14	4	M
411	ENSGALG00000053144	U3	Small nucleolar RNA U3 [Source:RFAM;Acc:RF00012]	1.54285380713905e-13	0,396018777	0.442	0.179	3,76E-09	4	M
242	ENSGALG00000026077	ALDOC	aldolase, fructose-bisphosphate C [Source:NCBI gene;Acc:395492]	4.68778900019581e-13	0,394232467	0.885	0.653	1,14E-08	4	M
240	ENSGALG00000025958	U3	Small nucleolar RNA U3 [Source:RFAM;Acc:RF00012]	1.09051552961124e-14	0,387789762	0.538	0.243	2,66E-10	4	M
305	ENSGALG00000032930	NCL	nucleolin [Source:NCBI gene;Acc:396201]	1.39950232156141e-22	0,380420745	1	0.991	3,41E-18	4	M
19	ENSGALG00000001992	PKLR	pyruvate kinase, liver and RBC [Source:NCBI gene;Acc:396456]	6.03432611595991e-13	0,37523326	0.894	0.682	1,47E-08	4	M
389	ENSGALG00000043033	EYA1	EYA transcriptional coactivator and phosphatase 1 [Source:NCBI gene;Acc:395718]	4.29235486451793e-107	0,362654919	0.442	0.028	1,05E-102	4	M
386	ENSGALG00000042492	PITX2	paired like homeodomain 2 [Source:NCBI gene;Acc:395862]	1.95150600855264e-19	0,350121896	0.606	0.21	4,75E-15	4	M
362	ENSGALG00000038850	WNK2	WNK lysine deficient protein kinase 2 [Source:NCBI gene;Acc:415984]	1.88279906187115e-68	0,349439791	0.433	0.045	4,59E-64	4	M
144	ENSGALG00000010175	HSP90AB1	heat shock protein 90 alpha family class B member 1 [Source:NCBI gene;Acc:396188]	8.32074648779665e-17	0,346524106	1	0.966	2,03E-12	4	M
265	ENSGALG00000028821	GLIPR2	GLI pathogenesis related 2 [Source:NCBI gene;Acc:420999]	2.88146122962794e-23	0,344611562	0.587	0.187	7,02E-19	4	M
44	ENSGALG00000003578	FN1	fibronectin 1 [Source:NCBI gene;Acc:396133]	1.83972006651905e-08	0,340824606	0.635	0.38	0,000448082	4	M
302	ENSGALG00000032730	NOP56	NOP56 ribonucleoprotein [Source:NCBI gene;Acc:426574]	6.38172646228399e-13	0,333708488	0.99	0.762	1,55E-08	4	M
118	ENSGALG00000009179	TNFSF10	tumor necrosis factor superfamily member 10 [Source:NCBI gene;Acc:378894]	1.63759237573393e-22	0,330933888	0.529	0.169	3,99E-18	4	M
282	ENSGALG00000031211	LBX2	ladybird homeobox 2 [Source:NCBI gene;Acc:431414]	1.39619648280039e-147	0,319864561	0.423	0.017	3,40E-143	4	M
342	ENSGALG00000036798	COL4A1	collagen type IV alpha 1 chain [Source:NCBI gene;Acc:395530]	5.2801551579232e-52	0,304268019	0.375	0.044	1,29E-47	4	M
323	ENSGALG00000034590	CELF2	CUGBP Elav-like family member 2 [Source:NCBI gene;Acc:374111]	9.013304881978e-103	0,303926087	0.356	0.018	2,20E-98	4	M
186	ENSGALG00000013726	PAICS	phosphoribosylaminoimidazole carboxylase; phosphoribosylaminoimidazolesuccinocarboxamide sy	5.41827443625983e-08	0,299966927	0.817	0.62	0,001319675	4	M
379	ENSGALG00000041121	SLIT2	slit guidance ligand 2 [Source:NCBI gene;Acc:373967]	3.53389377568611e-34	0,295987001	0.385	0.068	8,61E-30	4	M
268	ENSGALG00000029260	H-RAS	HRas proto-oncogene, GTPase [Source:NCBI gene;Acc:396229]	1.11545028169667e-12	0,290583916	0.548	0.244	2,72E-08	4	M
192	ENSGALG00000014442	GAPDH	glyceraldehyde-3-phosphate dehydrogenase [Source:NCBI gene;Acc:374193]	1.87781895526694e-12	0,289727078	1	1	4,57E-08	4	M
55	ENSGALG00000004508	EYA2	EYA transcriptional coactivator and phosphatase 2 [Source:NCBI gene;Acc:395745]	1.51546348598412e-64	0,28759714	0.365	0.033	3,69E-60	4	M
149	ENSGALG00000010490	DPYSL4	dihydropyrimidinase like 4 [Source:NCBI gene;Acc:374022]	8.69332869832863e-17	0,28318511	0.462	0.159	2,12E-12	4	M
31	ENSGALG00000002448	CCT6A	chaperonin containing TCP1 subunit 6A [Source:NCBI gene;Acc:417541]	2.35776859436911e-08	0,283113342	0.913	0.773	0,000574258	4	M
185	ENSGALG00000013598	SCGN	secretagogin, EF-hand calcium binding protein [Source:NCBI gene;Acc:421001]	1.37139410405192e-130	0,282265779	0.163	0.001	3,34E-126	4	M
92	ENSGALG00000007606	PGAM1	phosphoglycerate mutase 1 [Source:NCBI gene;Acc:428969]	2.14742489568811e-09	0,28215953	0.933	0.729	5,23E-05	4	M
322	ENSGALG00000034458	S100A1	S100 calcium binding protein A1 [Source:NCBI gene;Acc:100858258]	2.32094555200258e-57	0,278620932	0.269	0.02	5,65E-53	4	M
103	ENSGALG00000008187	EIF3J	eukaryotic translation initiation factor 3 subunit J [Source:NCBI gene;Acc:415573]	3.12076811520625e-07	0,274358552	0.885	0.724	0,007600943	4	M
201	ENSGALG00000015010	NSG1	neuron specific gene family member 1 [Source:NCBI gene;Acc:395251]	8.35937475950803e-24	0,271448069	0.385	0.09	2,04E-19	4	M
125	ENSGALG00000009309	EIF5A2	eukaryotic translation initiation factor 5A [Source:NCBI gene;Acc:396545]	1.57846769373006e-09	0,26862739	0.981	0.933	3,84E-05	4	M
26	ENSGALG00000002197	NPM1	nucleophosmin [Source:NCBI gene;Acc:396203]	2.42537299411155e-17	0,264534506	1	0.999	5,91E-13	4	M
166	ENSGALG00000011708	MYLK	myosin light chain kinase [Source:NCBI gene;Acc:396445]	1.32618689107283e-15	0,260281835	0.269	0.067	3,23E-11	4	M
345	ENSGALG00000036883	MET	MET proto-oncogene, receptor tyrosine kinase [Source:NCBI gene;Acc:396134]	8.0185344163966e-83	0,259256979	0.231	0.009	1,95E-78	4	M
15	ENSGALG00000001717	SLC16A1	solute carrier family 16 member 1 [Source:NCBI gene;Acc:419875]	4.22864899765598e-09	0,258226554	0.635	0.378	0,000102993	4	M
320	ENSGALG00000034346	RDH10	retinol dehydrogenase 10 (all-trans) [Source:NCBI gene;Acc:420183]	1.30854038750463e-11	0,257679738	0.404	0.155	3,19E-07	4	M
100	ENSGALG00000008094	HSPD1	heat shock protein family D (Hsp60) member 1 [Source:NCBI gene;Acc:424059]	1.54589241830541e-07	0,255930264	0.981	0.88	0,003765176	4	M
390	ENSGALG00000043035	SHISA2	shisa family member 2 [Source:NCBI gene;Acc:395162]	4.24965804773602e-12	0,254454557	0.337	0.115	1,04E-07	4	M
138	ENSGALG00000009739	AMIGO2	adhesion molecule with Ig like domain 2 [Source:NCBI gene;Acc:417810]	4.84200180862528e-85	0,252530497	0.288	0.014	1,18E-80	4	M
364	ENSGALG00000039145	CA8	carbonic anhydrase 8 [Source:NCBI gene;Acc:421139]	2.67911068839042e-185	0,252215914	0.308	0.005	6,53E-181	4	M

Supplementary Table 1

Markers for the CT and muscle clusters at E6

List of CT and muscle markers associated to the CT and muscle clusters at E6.

In the “Cluster type” column, “CT” or “M” means that the cluster respectively has a CT or a M identity.

	ensembl_gene_id	external_gene_name	description	p_val	avg_logFC	pct.1	pct.2	p_val_adj	cluster	cluster type
361	ENSGALG00000032039	HOXD13	homeobox D13 [Source:NCBI gene;Acc:396415]	3.56092014500129e-249	0,803036013	0.828	0.168	8,67E-245	0	CT
139	ENSGALG00000009274	HOXD12	homeobox D12 [Source:NCBI gene;Acc:396178]	1.48200075412607e-159	0,745717891	0.872	0.321	3,61E-155	0	CT
8	ENSGALG00000000713	ZFHX3	zinc finger homeobox 3 [Source:NCBI gene;Acc:395682]	9.88155387186301e-60	0,592413733	0.735	0.441	2,41E-55	0	CT
387	ENSGALG00000034346	RDH10	retinol dehydrogenase 10 (all-trans) [Source:NCBI gene;Acc:420183]	6.32207595364248e-28	0,568005329	0.207	0.069	1,54E-23	0	CT
28	ENSGALG00000003324	PRRX1	paired related homeobox 1 [Source:NCBI gene;Acc:373941]	4.90368534169849e-88	0,549562666	0.914	0.538	1,19E-83	0	CT
239	ENSGALG00000015015	CYTL1	cytokine like 1 [Source:NCBI gene;Acc:422849]	7.43376476696213e-64	0,395946621	0.406	0.123	1,81E-59	0	CT
83	ENSGALG00000006300	LDHA	lactate dehydrogenase A [Source:NCBI gene;Acc:396221]	9.79664863496393e-43	0,37967635	0.446	0.193	2,39E-38	0	CT
220	ENSGALG00000014233	FBLN1	fibulin 1 [Source:NCBI gene;Acc:373979]	2.85644252017031e-56	0,373006897	0.685	0.357	6,96E-52	0	CT
385	ENSGALG00000034168	WNT5A	Wnt family member 5A [Source:NCBI gene;Acc:395703]	1.60396860627033e-52	0,34792381	0.743	0.384	3,91E-48	0	CT
168	ENSGALG00000010652	SCP2	sterol carrier protein 2 [Source:NCBI gene;Acc:396550]	3.16261645492014e-47	0,345567584	0.61	0.32	7,70E-43	0	CT
140	ENSGALG00000009286	ATP5G3	ATP synthase, H+ transporting, mitochondrial Fo complex subunit C3 (subunit 9) [Source:NCBI gene	1.09027095466508e-50	0,340977955	0.97	0.875	2,66E-46	0	CT
450	ENSGALG00000040297	NBL1	neuroblastoma 1, DAN family BMP antagonist [Source:NCBI gene;Acc:373952]	1.82372657848103e-28	0,321348388	0.745	0.525	4,44E-24	0	CT
98	ENSGALG00000007131	HMGN5	high mobility group nucleosome binding domain 5 [Source:NCBI gene;Acc:422278]	1.02428330146271e-42	0,318760666	0.949	0.796	2,49E-38	0	CT
484	ENSGALG00000043329	PTMA	prothymosin, alpha [Source:NCBI gene;Acc:424931]	1.04681402656022e-69	0,302296897	0.998	0.997	2,55E-65	0	CT
263	ENSGALG00000016397	SOX11	SRY-box 11 [Source:NCBI gene;Acc:396104]	9.29797617034496e-34	0,28745156	0.663	0.416	2,26E-29	0	CT
292	ENSGALG00000021047	KIFAP3	kinesin associated protein 3 [Source:NCBI gene;Acc:424395]	4.23338556337449e-41	0,26952886	0.476	0.221	1,03E-36	0	CT
47	ENSGALG00000003803	DAB2	DAB2, clathrin adaptor protein [Source:NCBI gene;Acc:427449]	1.05746420112598e-54	0,251020406	0.381	0.119	2,58E-50	0	CT
399	ENSGALG00000035998	HINTW	histidine triad nucleotide binding protein W [Source:NCBI gene;Acc:395423]	2.55823781903937e-298	1,592445912	0.626	0.04	6,23E-294	1	CT
126	ENSGALG00000008677	VIM	vimentin [Source:NCBI gene;Acc:420519]	3.4951012161069e-45	1,102468538	0.849	0.846	8,51E-41	1	CT
206	ENSGALG00000012834	AKR1D1	aldo-keto reductase family 1 member D1 [Source:NCBI gene;Acc:418107]	5.91491233333393e-68	0,782301237	0.809	0.58	1,44E-63	1	CT
55	ENSGALG00000004274	TWIST2	twist family bHLH transcription factor 2 [Source:NCBI gene;Acc:395405]	6.28800178524285e-52	0,717952469	0.593	0.336	1,53E-47	1	CT
436	ENSGALG00000039118	MEIS2	Meis homeobox 2 [Source:NCBI gene;Acc:395588]	3.94838179674076e-220	0,680357352	0.572	0.061	9,62E-216	1	CT
96	ENSGALG00000007000	NR2F2	nuclear receptor subfamily 2 group F member 2 [Source:NCBI gene;Acc:386585]	8.25848529981507e-81	0,671858582	0.751	0.398	2,01E-76	1	CT
185	ENSGALG00000011469	IGFBP2	insulin like growth factor binding protein 2 [Source:NCBI gene;Acc:396315]	1.6876167005794e-93	0,584757544	0.632	0.243	4,11E-89	1	CT
36	ENSGALG00000003578	FN1	fibronectin 1 [Source:NCBI gene;Acc:396133]	1.72160683843456e-80	0,576030684	0.781	0.403	4,19E-76	1	CT
254	ENSGALG00000015720	CHODL	chondrolectin [Source:NCBI gene;Acc:418474]	2.84657215305602e-79	0,517516636	0.353	0.073	6,93E-75	1	CT
277	ENSGALG00000017046	POSTN	periostin [Source:NCBI gene;Acc:395429]	7.1826352915898e-47	0,495313913	0.208	0.04	1,75E-42	1	CT
229	ENSGALG00000014873	GJA1	gap junction protein alpha 1 [Source:NCBI gene;Acc:395278]	2.8712099097426e-22	0,484721853	0.652	0.556	6,99E-18	1	CT
324	ENSGALG00000028041	DKK2	dickkopf WNT signaling pathway inhibitor 2 [Source:NCBI gene;Acc:422531]	2.20476540750696e-61	0,484642278	0.409	0.126	5,37E-57	1	CT
452	ENSGALG00000040316	GPC1	glypican 1 [Source:NCBI gene;Acc:424770]	6.11986239183663e-45	0,458784937	0.717	0.491	1,49E-40	1	CT
306	ENSGALG00000026383	TMSB4X	thymosin beta 4, X-linked [Source:NCBI gene;Acc:408047]	3.74876743811722e-66	0,422548411	0.998	0.975	9,13E-62	1	CT
3	ENSGALG00000000242	EBF2	early B-cell factor 2 [Source:NCBI gene;Acc:395176]	4.54346429189829e-49	0,414851408	0.325	0.098	1,11E-44	1	CT
372	ENSGALG00000032687	PHLDA2	pleckstrin homology like domain family A member 2 [Source:NCBI gene;Acc:423088]	5.24658311798522e-29	0,386695819	0.474	0.266	1,28E-24	1	CT
392	ENSGALG00000035016	ID2	inhibitor of DNA binding 2, HLH protein [Source:NCBI gene;Acc:395852]	6.18670771780948e-23	0,37914055	0.636	0.487	1,51E-18	1	CT
59	ENSGALG00000004376	KCNJ2	Potassium inwardly rectifying channel subfamily J member 2 [Source:NCBI gene;Acc:396328]	2.41294257099769e-56	0,342568226	0.29	0.067	5,88E-52	1	CT
198	ENSGALG00000012702	JARID2	jumonji and AT-rich interaction domain containing 2 [Source:NCBI gene;Acc:420839]	1.05913617056088e-32	0,319608382	0.426	0.219	2,58E-28	1	CT
70	ENSGALG00000005503	WFDC1	WAP four-disulfide core domain 1 [Source:NCBI gene;Acc:395238]	1.83553952511293e-47	0,306011308	0.288	0.082	4,47E-43	1	CT
252	ENSGALG00000015708	FGFR3	fibroblast growth factor receptor 3 [Source:NCBI gene;Acc:396515]	3.7692371189361e-33	0,303854266	0.258	0.083	9,18E-29	1	CT
177	ENSGALG00000011271	LUM	lumican [Source:NCBI gene;Acc:417891]	3.95633265614713e-19	0,300681594	0.452	0.277	9,64E-15	1	CT
51	ENSGALG00000003923	COL6A3	collagen type VI alpha 3 chain [Source:NCBI gene;Acc:396548]	1.11306269320045e-39	0,298924896	0.327	0.114	2,71E-35	1	CT
57	ENSGALG00000004290	ANGPTL1	angiopoietin like 1 [Source:NCBI gene;Acc:770850]	1.07841543787073e-40	0,288639049	0.353	0.129	2,63E-36	1	CT
488	ENSGALG00000043690	ECRG4	chromosome 1 open reading frame, human C2orf40 [Source:NCBI gene;Acc:771055]	1.15426474784464e-22	0,28155113	0.563	0.374	2,81E-18	1	CT
190	ENSGALG00000011687	AHNAK2	AHNAK nucleoprotein 2 [Source:NCBI gene;Acc:100859120]	7.07413446002737e-20	0,275471799	0.266	0.127	1,72E-15	1	CT
154	ENSGALG00000009654	mylk1	myeloid leukemia factor 1 [Source:NCBI gene;Acc:425019]	1.25410675403487e-24	0,274034251	0.351	0.179	3,05E-20	1	CT
464	ENSGALG00000041346	CXCL12	C-X-C motif chemokine ligand 12 [Source:NCBI gene;Acc:395180]	1.18920552790942e-09	0,266048124	0.19	0.105	2,90E-05	1	CT
439	ENSGALG00000039216	COL6A2	collagen type VI alpha 2 chain [Source:NCBI gene;Acc:396292]	1.12321291132979e-41	0,259968594	0.284	0.087	2,74E-37	1	CT
232	ENSGALG00000014910	COLEC12	collectin subfamily member 12 [Source:NCBI gene;Acc:421061]	2.05190420900431e-17	0,25608783	0.379	0.233	5,00E-13	1	CT
12	ENSGALG00000000839	WNT11	Wnt family member 11 [Source:NCBI gene;Acc:395562]	1.5127327741057e-33	0,254613349	0.299	0.109	3,68E-29	1	CT

326	ENSGALG00000028354	METRNL	meteorin, glial cell differentiation regulator-like [Source:NCBI gene;Acc:417332]	8.64588716378244e-22	0,253300612	0.353	0.188	2,11E-17	1	CT
238	ENSGALG00000015013	MSX1	msh homeobox 1 [Source:NCBI gene;Acc:396484]	3.2885615451014e-244	1,033890646	0.795	0.15	8,01E-240	2	CT
433	ENSGALG00000038848	MSX2	msh homeobox 2 [Source:NCBI gene;Acc:395245]	1.23013022697864e-302	0,948324935	0.804	0.108	3,00E-298	2	CT
384	ENSGALG00000034168	WNT5A	Wnt family member 5A [Source:NCBI gene;Acc:395703]	3.93805500941348e-119	0,643565702	0.848	0.374	9,59E-115	2	CT
30	ENSGALG00000003324	PRRX1	paired related homeobox 1 [Source:NCBI gene;Acc:373941]	2.96604030185312e-87	0,616595532	0.93	0.545	7,22E-83	2	CT
408	ENSGALG00000037015	DACH1	dachshund family transcription factor 1 [Source:NCBI gene;Acc:373935]	5.13748743456523e-155	0,582237512	0.534	0.091	1,25E-150	2	CT
136	ENSGALG00000009129	DLX5	distal-less homeobox 5 [Source:NCBI gene;Acc:373969]	7.99369453852708e-241	0,564909516	0.557	0.042	1,95E-236	2	CT
316	ENSGALG00000027234	HOXA13	homeobox A13 [Source:NCBI gene;Acc:373934]	6.15683632897285e-108	0,503737012	0.633	0.195	1,50E-103	2	CT
459	ENSGALG00000040993	BAMBI	BMP and activin membrane bound inhibitor [Source:NCBI gene;Acc:428413]	4.89674456882042e-120	0,402624506	0.447	0.076	1,19E-115	2	CT
178	ENSGALG00000011271	LUM	lumican [Source:NCBI gene;Acc:417891]	2.72123179512402e-52	0,385507461	0.586	0.253	6,63E-48	2	CT
261	ENSGALG00000016348	SAT1	spermidine/spermine N1-acetyltransferase 1 [Source:NCBI gene;Acc:374006]	4.21398799175118e-44	0,376361715	0.46	0.194	1,03E-39	2	CT
200	ENSGALG00000012775	TFAP2A	transcription factor AP-2 alpha [Source:NCBI gene;Acc:395982]	2.75181812204017e-100	0,369256884	0.414	0.079	6,70E-96	2	CT
48	ENSGALG00000003891	ZNF423	zinc finger protein 423 [Source:NCBI gene;Acc:415738]	1.21591426482228e-51	0,366998137	0.709	0.39	2,96E-47	2	CT
248	ENSGALG00000015624	VCAN	versican [Source:NCBI gene;Acc:395565]	9.1981916301451e-44	0,366875874	0.747	0.417	2,24E-39	2	CT
393	ENSGALG00000035027	ENSGALG00000035027		5.61031329342484e-165	0,365416911	0.302	0.008	1,37E-160	2	CT
494	ENSGALG00000046616	CHGB	chromogranin B [Source:NCBI gene;Acc:421312]	3.61752140730257e-08	0,35748314	0.137	0.069	0,000881084	2	CT
42	ENSGALG00000003670	MAFB	MAF bZIP transcription factor B [Source:NCBI gene;Acc:419173]	1.17971120012986e-18	0,345233441	0.264	0.12	2,87E-14	2	CT
221	ENSGALG00000014485	LDB2	LIM domain binding 2 [Source:NCBI gene;Acc:395631]	5.47034769724627e-34	0,325857329	0.492	0.244	1,33E-29	2	CT
332	ENSGALG00000028685	SEMA5A	semaphorin 5A [Source:NCBI gene;Acc:420931]	2.42205978108715e-45	0,296844738	0.394	0.139	5,90E-41	2	CT
79	ENSGALG00000006087	GPC3	glypican 3 [Source:NCBI gene;Acc:770863]	2.71434057440196e-99	0,294876088	0.291	0.032	6,61E-95	2	CT
235	ENSGALG00000014970	FSTL1	follistatin like 1 [Source:NCBI gene;Acc:395349]	9.49606690455919e-28	0,276504144	0.857	0.663	2,31E-23	2	CT
463	ENSGALG00000041263	DCLK1	doublecortin like kinase 1 [Source:NCBI gene;Acc:418903]	1.00692410865887e-49	0,263581013	0.283	0.07	2,45E-45	2	CT
26	ENSGALG00000003193	CRABP-I	cellular retinoic acid binding protein 1 [Source:NCBI gene;Acc:374211]	1.72760436254358e-74	1,127586662	0.847	0.566	4,21E-70	3	CT
455	ENSGALG00000040493	PTN	pleiotrophin [Source:NCBI gene;Acc:418125]	2.49611790990837e-87	1,127261829	0.707	0.272	6,08E-83	3	CT
354	ENSGALG00000031425	PMP22	peripheral myelin protein 22 [Source:NCBI gene;Acc:417327]	4.58774319974883e-61	0,799532683	0.456	0.15	1,12E-56	3	CT
132	ENSGALG00000008883	TCF7L2	transcription factor 7-like 2 (T-cell specific, HMG-box) [Source:NCBI gene;Acc:395508]	3.65204862966015e-62	0,764454401	0.835	0.56	8,89E-58	3	CT
35	ENSGALG00000003578	FN1	fibronectin 1 [Source:NCBI gene;Acc:396133]	1.52864251398659e-60	0,732046811	0.769	0.422	3,72E-56	3	CT
466	ENSGALG00000041555	COL1A1	collagen type I alpha 1 chain [Source:NCBI gene;Acc:395532]	6.02936633370293e-56	0,706623637	0.972	0.908	1,47E-51	3	CT
474	ENSGALG00000042134	PIEZO2	piezo type mechanosensitive ion channel component 2 [Source:NCBI gene;Acc:421052]	1.34884791023034e-47	0,686649288	0.398	0.136	3,29E-43	3	CT
17	ENSGALG00000002546	COL5A1	collagen type V alpha 1 chain [Source:NCBI gene;Acc:395568]	3.25912781951119e-69	0,630010954	0.917	0.702	7,94E-65	3	CT
122	ENSGALG00000008367	MDK	midkine (neurite growth-promoting factor 2) [Source:NCBI gene;Acc:423196]	4.09710542258577e-36	0,567679586	0.835	0.66	9,98E-32	3	CT
41	ENSGALG00000003655	GAS2	growth arrest specific 2 [Source:NCBI gene;Acc:422970]	6.05811677779682e-64	0,559753451	0.689	0.339	1,48E-59	3	CT
382	ENSGALG00000034048	BCL11A	B-cell CLL/lymphoma 11A [Source:NCBI gene;Acc:421199]	1.25994529744968e-31	0,550199478	0.697	0.503	3,07E-27	3	CT
166	ENSGALG00000010461	EBF3	early B cell factor 3 [Source:NCBI gene;Acc:395175]	2.49545930292801e-30	0,545229457	0.614	0.384	6,08E-26	3	CT
445	ENSGALG00000039985	CDH11	cadherin 11 [Source:NCBI gene;Acc:415797]	2.46177109886377e-51	0,516036805	0.89	0.673	6,00E-47	3	CT
242	ENSGALG00000015193	CCDC80	coiled-coil domain containing 80 [Source:NCBI gene;Acc:395074]	2.69273669039396e-36	0,500574105	0.742	0.494	6,56E-32	3	CT
262	ENSGALG00000016364	ALKAL2	family with sequence similarity 150 member B [Source:NCBI gene;Acc:421910]	1.04276614761947e-98	0,459594569	0.248	0.017	2,54E-94	3	CT
138	ENSGALG00000009241	SFRP2	secreted frizzled related protein 2 [Source:NCBI gene;Acc:395546]	1.08186004282019e-35	0,452594938	0.459	0.206	2,63E-31	3	CT
337	ENSGALG00000029401	SIX1	SIX homeobox 1 [Source:NCBI gene;Acc:693262]	9.46394939072556e-35	0,448607478	0.469	0.209	2,31E-30	3	CT
148	ENSGALG00000009568	SPOCK3	SPARC/osteonectin, cwcv and kazal like domains proteoglycan 3 [Source:NCBI gene;Acc:422425]	5.68439823535539e-20	0,432624548	0.396	0.225	1,38E-15	3	CT
331	ENSGALG00000028627	TIMP3	TIMP metallopeptidase inhibitor 3 [Source:NCBI gene;Acc:396483]	6.5856982476162e-30	0,428877941	0.476	0.25	1,60E-25	3	CT
295	ENSGALG00000023904	FIBIN	fin bud initiation factor homolog (zebrafish) [Source:NCBI gene;Acc:426933]	2.61619264607745e-44	0,411428778	0.441	0.162	6,37E-40	3	CT
320	ENSGALG00000027891	NREP	neuronal regeneration related protein [Source:NCBI gene;Acc:396353]	2.76057576641592e-33	0,404932664	0.794	0.62	6,72E-29	3	CT
180	ENSGALG00000011274	DCN	decorin [Source:NCBI gene;Acc:417892]	3.04699639590926e-21	0,404498354	0.764	0.602	7,42E-17	3	CT
473	ENSGALG00000042077	HIC1	HIC ZBTB transcriptional repressor 1 [Source:NCBI gene;Acc:396164]	1.95046194039036e-48	0,396660867	0.459	0.158	4,75E-44	3	CT
491	ENSGALG00000044235	MAB21L2	mab-21 like 2 [Source:NCBI gene;Acc:374011]	1.11736533654893e-52	0,383181631	0.386	0.111	2,72E-48	3	CT
339	ENSGALG00000029429	TGFBI	transforming growth factor beta induced [Source:NCBI gene;Acc:395897]	4.59803874832132e-14	0,346845688	0.414	0.268	1,12E-09	3	CT
322	ENSGALG00000027905	CSRNP1	cysteine and serine rich nuclear protein 1 [Source:NCBI gene;Acc:420425]	3.70126300630329e-30	0,31771673	0.406	0.182	9,01E-26	3	CT
91	ENSGALG00000006609	EPHB1	EPH receptor B1 [Source:NCBI gene;Acc:396177]	4.79511783326666e-17	0,316014603	0.414	0.247	1,17E-12	3	CT
115	ENSGALG00000008253	TBX5	T-box 5 [Source:NCBI gene;Acc:373988]	1.44942854334883e-23	0,304163864	0.744	0.532	3,53E-19	3	CT
213	ENSGALG00000013672	TSHZ1	teashirt zinc finger homeobox 1 [Source:NCBI gene;Acc:428513]	4.07634143123398e-36	0,30062399	0.316	0.101	9,93E-32	3	CT
485	ENSGALG00000043433	SCX	scleraxis bHLH transcription factor [Source:NCBI gene;Acc:374101]	6.74439800601384e-30	0,297939042	0.336	0.129	1,64E-25	3	CT
78	ENSGALG00000006080	GPC4	glypican 4 [Source:NCBI gene;Acc:422234]	1.692091808948e-19	0,290214209	0.419	0.238	4,12E-15	3	CT
20	ENSGALG00000002763	FRZB	frizzled related protein [Source:NCBI gene;Acc:395545]	9.99375306329956e-14	0,289010301	0.393	0.25	2,43E-09	3	CT
33	ENSGALG00000003446	PRLR	prolactin receptor [Source:NCBI gene;Acc:395660]	4.71129967214149e-42	0,274057801	0.208	0.039	1,15E-37	3	CT

253	ENSGALG00000015720	CHODL	chondrolectin [Source:NCBI gene;Acc:418474]	9.79358807787629e-08	0,271971908	0.195	0.108	0,002385326	3	CT
151	ENSGALG00000009612	TGFB2	transforming growth factor beta 2 [Source:NCBI gene;Acc:421352]	1.80743472484985e-06	0,261633257	0.383	0.289	0,04402188	3	CT
63	ENSGALG00000004812	FAM129A	family with sequence similarity 129 member A [Source:NCBI gene;Acc:424451]	1.44701354817739e-31	0,258899461	0.221	0.058	3,52E-27	3	CT
246	ENSGALG00000015419	PENK	proenkephalin [Source:NCBI gene;Acc:421131]	3.00815212456535e-176	1,634016733	0.424	0.028	7,33E-172	4	M
477	ENSGALG00000042492	PITX2	paired like homeodomain 2 [Source:NCBI gene;Acc:395862]	0	1,220459832	0.833	0.018	0	4	M
199	ENSGALG00000012712	RBM24	RNA binding motif protein 24 [Source:NCBI gene;Acc:420846]	8.95626021189759e-279	1,178001587	0.535	0.019	2,18E-274	4	M
159	ENSGALG00000009844	ACTC1	actin alpha cardiac muscle 1 [Source:NCBI gene;Acc:423298]	2.59424803655692e-50	1,091170085	0.17	0.019	6,32E-46	4	M
188	ENSGALG00000011511	CKB	creatine kinase B [Source:NCBI gene;Acc:396248]	1.15693385126828e-146	1,066677435	0.55	0.088	2,82E-142	4	M
328	ENSGALG00000028543	FGFR4	fibroblast growth factor receptor 4 [Source:NCBI gene;Acc:395603]	0	0,882972499	0.776	0.018	0	4	M
319	ENSGALG00000027887	C1QTNF3	C1q and tumor necrosis factor related protein 3 [Source:NCBI gene;Acc:427430]	0	0,839444548	0.506	0.006	0	4	M
80	ENSGALG00000006216	MYOD1	myogenic differentiation 1 [Source:NCBI gene;Acc:374048]	6.8499746370292e-279	0,836279526	0.458	0.006	1,67E-274	4	M
39	ENSGALG00000003642	PDGFA	platelet derived growth factor subunit A [Source:NCBI gene;Acc:374196]	0	0,80568137	0.684	0.037	0	4	M
94	ENSGALG00000006835	TNNC2	troponin C2, fast skeletal type [Source:NCBI gene;Acc:396434]	1.64908828659747e-272	0,735874187	0.55	0.023	4,02E-268	4	M
338	ENSGALG00000029401	SIX1	SIX homeobox 1 [Source:NCBI gene;Acc:693262]	1.41136493320897e-172	0,705185535	0.81	0.165	3,44E-168	4	M
359	ENSGALG00000031593	TMSB15B	thymosin beta 15B [Source:NCBI gene;Acc:100502566]	1.07180293147468e-44	0,679336878	0.941	0.793	2,61E-40	4	M
309	ENSGALG00000026383	TMSB4X	thymosin beta 4, X-linked [Source:NCBI gene;Acc:408047]	3.21747972098097e-57	0,526371091	0.995	0.976	7,84E-53	4	M
150	ENSGALG00000009612	TGFB2	transforming growth factor beta 2 [Source:NCBI gene;Acc:421352]	8.23528465560153e-81	0,503954009	0.735	0.243	2,01E-76	4	M
4	ENSGALG00000000313	TNNI1	troponin I type 1 (skeletal, slow) [Source:NCBI gene;Acc:421161]	6.03551855151616e-227	0,481625781	0.406	0.009	1,47E-222	4	M
286	ENSGALG00000017644	COTL1	coactosin like F-actin binding protein 1 [Source:NCBI gene;Acc:768420]	3.32042355120156e-57	0,473880592	0.728	0.351	8,09E-53	4	M
366	ENSGALG00000032329	NPM3	nucleophosmin/nucleoplasmin 3 [Source:NCBI gene;Acc:770430]	2.03025588209457e-103	0,455482933	0.499	0.098	4,94E-99	4	M
454	ENSGALG00000040465	ZEB2	zinc finger E-box binding homeobox 2 [Source:NCBI gene;Acc:424306]	2.49452900697028e-184	0,440922198	0.491	0.039	6,08E-180	4	M
483	ENSGALG00000043204	PAX7	paired box 7 [Source:NCBI gene;Acc:395942]	8.153906543966e-259	0,407309569	0.424	0.005	1,99E-254	4	M
211	ENSGALG00000013239	ENSGALG00000013239		1.69008580940647e-62	0,405667151	0.581	0.199	4,12E-58	4	M
61	ENSGALG00000004518	BHMT2	betaine--homocysteine S-methyltransferase 2 [Source:NCBI gene;Acc:416371]	7.91972504956054e-169	0,403682925	0.321	0.009	1,93E-164	4	M
365	ENSGALG00000032316	CDV3	CDV3 homolog [Source:NCBI gene;Acc:420693]	3.57387825052831e-39	0,393834303	0.81	0.517	8,70E-35	4	M
161	ENSGALG00000010175	HSP90AB1	heat shock protein 90 alpha family class B member 1 [Source:NCBI gene;Acc:396188]	9.69347294777178e-41	0,37341635	0.961	0.801	2,36E-36	4	M
5	ENSGALG00000000585	MYL4	myosin, light chain 4, alkali; atrial, embryonic [Source:NCBI gene;Acc:396472]	3.69021879273538e-122	0,372600271	0.38	0.04	8,99E-118	4	M
380	ENSGALG00000033677	AHCY	adenosylhomocysteinase [Source:NCBI gene;Acc:419146]	4.53547944551066e-31	0,36505742	0.674	0.397	1,10E-26	4	M
351	ENSGALG00000030587	SPTBB4L	spectrin beta chain, brain 4-like [Source:NCBI gene;Acc:421441]	3.49374672489165e-76	0,361694586	0.226	0.021	8,51E-72	4	M
256	ENSGALG00000016281	DMD	dystrophin [Source:NCBI gene;Acc:396236]	6.6234385880944e-82	0,36118963	0.47	0.104	1,61E-77	4	M
304	ENSGALG00000026276	TCF15	transcription factor 15 (basic helix-loop-helix) [Source:NCBI gene;Acc:395788]	1.87020703342399e-207	0,353915329	0.391	0.011	4,56E-203	4	M
113	ENSGALG00000008094	HSPD1	heat shock protein family D (Hsp60) member 1 [Source:NCBI gene;Acc:424059]	4.5003859242094e-30	0,348921929	0.82	0.612	1,10E-25	4	M
299	ENSGALG00000025958	U3	Small nucleolar RNA U3 [Source:RFAM;Acc:RF00012]	8.34931985460867e-52	0,34754274	0.514	0.182	2,03E-47	4	M
375	ENSGALG00000032930	NCL	nucleolin [Source:NCBI gene;Acc:396201]	1.60574270077489e-41	0,346315278	0.967	0.917	3,91E-37	4	M
131	ENSGALG00000008883	TCF7L2	transcription factor 7-like 2 (T-cell specific, HMG-box) [Source:NCBI gene;Acc:395508]	5.78026276183523e-28	0,338258896	0.828	0.562	1,41E-23	4	M
145	ENSGALG00000009309	EIF5A2	eukaryotic translation initiation factor 5A [Source:NCBI gene;Acc:396545]	9.04504070921002e-32	0,331962777	0.853	0.658	2,20E-27	4	M
348	ENSGALG00000030040	CDH15	acyl-CoA synthetase family member 3 [Source:NCBI gene;Acc:427561]	8.36798078135706e-182	0,326895484	0.383	0.017	2,04E-177	4	M
279	ENSGALG00000017179	PDGFD	platelet derived growth factor D [Source:NCBI gene;Acc:418978]	3.08371389216166e-82	0,326273364	0.46	0.097	7,51E-78	4	M
461	ENSGALG00000041080	CHD7	chromodomain helicase DNA binding protein 7 [Source:NCBI gene;Acc:421140]	1.66629118876678e-66	0,311720232	0.573	0.177	4,06E-62	4	M
68	ENSGALG00000005263	SOX8	SRY-box 8 [Source:NCBI gene;Acc:395483]	6.03714699966747e-49	0,309902988	0.437	0.138	1,47E-44	4	M
170	ENSGALG00000010745	HMGB2	high mobility group box 2 [Source:NCBI gene;Acc:396482]	6.2790193783411e-20	0,30744782	0.774	0.529	1,53E-15	4	M
495	ENSGALG00000046927	ENSGALG00000046927		1.32958575448022e-160	0,30050437	0.362	0.019	3,24E-156	4	M
437	ENSGALG00000039145	CA8	carbonic anhydrase 8 [Source:NCBI gene;Acc:421139]	3.32763405991281e-148	0,292300196	0.28	0.008	8,10E-144	4	M
512	ENSGALG00000053371	ENSGALG00000053371		2.08788172320846e-38	0,290007839	0.481	0.187	5,09E-34	4	M
373	ENSGALG00000032730	NOP56	NOP56 ribonucleoprotein [Source:NCBI gene;Acc:426574]	2.28582153666462e-24	0,2868117	0.781	0.536	5,57E-20	4	M
402	ENSGALG00000036441	WIPF1	WAS/WASL interacting protein family member 1 [Source:NCBI gene;Acc:424143]	7.34265747420829e-62	0,281677752	0.285	0.049	1,79E-57	4	M
219	ENSGALG00000014023	H2AFZ	H2A histone family, member Z [Source:NCBI gene;Acc:426361]	6.88974207643521e-16	0,267798872	0.846	0.718	1,68E-11	4	M
509	ENSGALG00000053144	U3	Small nucleolar RNA U3 [Source:RFAM;Acc:RF00012]	3.04926836869006e-48	0,267527973	0.429	0.133	7,43E-44	4	M
389	ENSGALG00000034590	CELF2	CUGBP Elav-like family member 2 [Source:NCBI gene;Acc:374111]	9.50713736888914e-108	0,262569654	0.368	0.041	2,32E-103	4	M
344	ENSGALG00000029921	CSRP2	cysteine and glycine rich protein 2 [Source:NCBI gene;Acc:396128]	5.835644620093e-43	0,250378253	0.419	0.135	1,42E-38	4	M
400	ENSGALG00000036240	AKAP12	A-kinase anchoring protein 12 [Source:NCBI gene;Acc:421634]	6.13593393574994e-49	0,8410708	0.764	0.495	1,49E-44	5	CT
167	ENSGALG00000010461	EBF3	early B cell factor 3 [Source:NCBI gene;Acc:395175]	7.26569565418474e-59	0,787950651	0.751	0.377	1,77E-54	5	CT
370	ENSGALG00000032687	PHLDA2	pleckstrin homology like domain family A member 2 [Source:NCBI gene;Acc:423088]	9.32662488858179e-49	0,737599416	0.617	0.266	2,27E-44	5	CT
296	ENSGALG00000024488	CD99	CD99 molecule (Xg blood group) [Source:NCBI gene;Acc:418662]	1.61732908477814e-56	0,610675506	0.537	0.193	3,94E-52	5	CT
18	ENSGALG00000002546	COL5A1	collagen type V alpha 1 chain [Source:NCBI gene;Acc:395568]	6.95989728078632e-45	0,546121549	0.911	0.709	1,70E-40	5	CT

90	ENSGALG00000006583	LSP1P1	lymphocyte-specific protein 1 pseudogene 1 [Source:NCBI gene;Acc:374254]	8.93830281576886e-44	0,545683987	0.553	0.246	2,18E-39	5	CT
472	ENSGALG00000042077	HIC1	HIC ZBTB transcriptional repressor 1 [Source:NCBI gene;Acc:396164]	3.4143860479804e-71	0,544761778	0.559	0.156	8,32E-67	5	CT
181	ENSGALG00000011274	DCN	decorin [Source:NCBI gene;Acc:417892]	2.56795295669565e-39	0,51246345	0.866	0.596	6,25E-35	5	CT
346	ENSGALG00000030008	HHEX	hematopoietically expressed homeobox [Source:NCBI gene;Acc:396182]	5.67528157667905e-41	0,470906265	0.201	0.033	1,38E-36	5	CT
54	ENSGALG00000004270	ALDH1A2	aldehyde dehydrogenase 1 family member A2 [Source:NCBI gene;Acc:395844]	3.48887766023878e-59	0,436852161	0.406	0.095	8,50E-55	5	CT
357	ENSGALG00000031487	FSTL4	follistatin like 4 [Source:NCBI gene;Acc:395163]	2.54290597657585e-43	0,429537227	0.339	0.091	6,19E-39	5	CT
117	ENSGALG00000008298	LRRC17	leucine rich repeat containing 17 [Source:NCBI gene;Acc:395440]	1.03697798014714e-24	0,423597189	0.565	0.322	2,53E-20	5	CT
449	ENSGALG00000040297	NBL1	neuroblastoma 1, DAN family BMP antagonist [Source:NCBI gene;Acc:373952]	2.49361063944066e-27	0,414313428	0.796	0.54	6,07E-23	5	CT
205	ENSGALG00000012834	AKR1D1	aldo-keto reductase family 1 member D1 [Source:NCBI gene;Acc:418107]	9.28073813513502e-22	0,404890664	0.802	0.597	2,26E-17	5	CT
440	ENSGALG00000039238	SALL1	spalt like transcription factor 1 [Source:NCBI gene;Acc:395446]	7.27630079609514e-49	0,394484569	0.39	0.109	1,77E-44	5	CT
245	ENSGALG00000015403	EPHA3	EPH receptor A3 [Source:NCBI gene;Acc:396402]	3.40503626990089e-39	0,386576539	0.358	0.105	8,29E-35	5	CT
305	ENSGALG00000026313	RND3	Rho family GTPase 3 [Source:NCBI gene;Acc:424312]	2.20752727009077e-23	0,382448342	0.486	0.259	5,38E-19	5	CT
471	ENSGALG00000041750	ADAMTS9	ADAM metallopeptidase with thrombospondin type 1 motif 9 [Source:NCBI gene;Acc:416080]	2.65280248364389e-50	0,374965798	0.355	0.083	6,46E-46	5	CT
77	ENSGALG00000005982	KLHL9	kelch like family member 9 [Source:NCBI gene;Acc:422226]	4.67553120086045e-26	0,36758374	0.371	0.158	1,14E-21	5	CT
192	ENSGALG00000011855	RHOJ	ras homolog family member J [Source:NCBI gene;Acc:423513]	5.7530384438464e-72	0,365373631	0.358	0.06	1,40E-67	5	CT
193	ENSGALG00000011962	NDNF	neuron-derived neurotrophic factor [Source:NCBI gene;Acc:422672]	1.1829983407361e-62	0,359818129	0.214	0.022	2,88E-58	5	CT
470	ENSGALG00000041650	DUSP7	dual specificity phosphatase 7 [Source:NCBI gene;Acc:415891]	2.4316017998164e-23	0,350163595	0.492	0.261	5,92E-19	5	CT
152	ENSGALG00000009612	TGFB2	transforming growth factor beta 2 [Source:NCBI gene;Acc:421352]	2.04438421637693e-17	0,345377313	0.498	0.28	4,98E-13	5	CT
95	ENSGALG00000007000	NR2F2	nuclear receptor subfamily 2 group F member 2 [Source:NCBI gene;Acc:386585]	2.18843745041646e-13	0,337688697	0.629	0.436	5,33E-09	5	CT
103	ENSGALG00000007331	SVIL	supervillin [Source:NCBI gene;Acc:420475]	5.95264696864279e-25	0,337048749	0.502	0.26	1,45E-20	5	CT
135	ENSGALG00000009052	LBH	limb bud and heart development [Source:NCBI gene;Acc:421301]	9.72447376942627e-23	0,323701538	0.265	0.093	2,37E-18	5	CT
247	ENSGALG00000015624	VCAN	versican [Source:NCBI gene;Acc:395565]	1.44770944982504e-17	0,320985065	0.661	0.449	3,53E-13	5	CT
174	ENSGALG00000010818	PLPP3	phospholipid phosphatase 3 [Source:NCBI gene;Acc:424666]	6.2511943768882e-25	0,314240215	0.556	0.292	1,52E-20	5	CT
38	ENSGALG00000003580	MMP2	matrix metallopeptidase 2 [Source:NCBI gene;Acc:386583]	5.63268293845225e-20	0,313394259	0.687	0.455	1,37E-15	5	CT
355	ENSGALG00000031425	PMP22	peripheral myelin protein 22 [Source:NCBI gene;Acc:417327]	1.43551115030395e-13	0,306293321	0.329	0.172	3,50E-09	5	CT
353	ENSGALG00000031424	SDC2	syndecan 2 [Source:NCBI gene;Acc:374102]	8.07875886699784e-10	0,301446034	0.339	0.214	1,97E-05	5	CT
412	ENSGALG00000037164	ZFHX4	zinc finger homeobox 4 [Source:NCBI gene;Acc:395904]	7.6273121349801e-17	0,300314728	0.789	0.618	1,86E-12	5	CT
446	ENSGALG00000039985	CDH11	cadherin 11 [Source:NCBI gene;Acc:415797]	2.98699116639028e-18	0,288940771	0.847	0.684	7,28E-14	5	CT
479	ENSGALG00000043035	SHISA2	shisa family member 2 [Source:NCBI gene;Acc:395162]	4.9995494101983e-10	0,281152209	0.371	0.233	1,22E-05	5	CT
330	ENSGALG00000028627	TIMP3	TIMP metallopeptidase inhibitor 3 [Source:NCBI gene;Acc:396483]	4.75276950485152e-17	0,277681076	0.454	0.259	1,16E-12	5	CT
303	ENSGALG00000026146	CRLF1	cytokine receptor like factor 1 [Source:NCBI gene;Acc:100858730]	2.95995507313406e-27	0,273721582	0.342	0.124	7,21E-23	5	CT
416	ENSGALG00000037506	SIPA1L2	signal induced proliferation associated 1 like 2 [Source:NCBI gene;Acc:421523]	2.78789949834995e-30	0,26107879	0.275	0.079	6,79E-26	5	CT
271	ENSGALG00000016584	EGFL6	EGF like domain multiple 6 [Source:HGNC Symbol;Acc:HGNC:3235]	3.03517707391953e-39	0,257911823	0.233	0.046	7,39E-35	5	CT
92	ENSGALG00000006609	EPHB1	EPH receptor B1 [Source:NCBI gene;Acc:396177]	4.33227555391516e-12	0,254854118	0.409	0.252	1,06E-07	5	CT
423	ENSGALG00000037901	EFEMP1	EGF containing fibulin like extracellular matrix protein 1 [Source:NCBI gene;Acc:428543]	3.06617845489507e-18	0,254516192	0.246	0.093	7,47E-14	5	CT
396	ENSGALG00000035599	CD24	CD24 molecule [Source:NCBI gene;Acc:100859742]	6.40747209894282e-19	0,252040526	0.329	0.144	1,56E-14	5	CT
215	ENSGALG00000013771	CRACD	capping protein inhibiting regulator of actin dynamics [Source:HGNC Symbol;Acc:HGNC:29219]	2.83823955566911e-67	0,251024896	0.217	0.02	6,91E-63	5	CT
312	ENSGALG00000026736	OGN	osteoglycin [Source:NCBI gene;Acc:374039]	7.61568985306701e-15	0,250238552	0.374	0.192	1,85E-10	5	CT
27	ENSGALG00000003193	CRABP-I	cellular retinoic acid binding protein 1 [Source:NCBI gene;Acc:374211]	1.35252912506967e-70	1,037521314	0.942	0.573	3,29E-66	6	CT
67	ENSGALG00000005209	AQP1	aquaporin 1 [Source:NCBI gene;Acc:420384]	2.67460672104272e-121	0,764569784	0.425	0.039	6,51E-117	6	CT
183	ENSGALG00000011274	DCN	decorin [Source:NCBI gene;Acc:417892]	1.21631491291725e-36	0,669610893	0.858	0.603	2,96E-32	6	CT
56	ENSGALG00000004274	TWIST2	twist family bHLH transcription factor 2 [Source:NCBI gene;Acc:395405]	3.28111452747454e-48	0,63076464	0.754	0.348	7,99E-44	6	CT
266	ENSGALG00000016457	TRIB2	tribbles pseudokinase 2 [Source:NCBI gene;Acc:378919]	1.46076825818699e-48	0,580464887	0.696	0.323	3,56E-44	6	CT
323	ENSGALG00000028041	DKK2	dickkopf WNT signaling pathway inhibitor 2 [Source:NCBI gene;Acc:422531]	6.33502192310861e-52	0,549299521	0.517	0.145	1,54E-47	6	CT
489	ENSGALG00000043690	ECRG4	chromosome 1 open reading frame, human C2orf40 [Source:NCBI gene;Acc:771055]	2.59126935828091e-33	0,516044134	0.721	0.38	6,31E-29	6	CT
413	ENSGALG00000037164	ZFHX4	zinc finger homeobox 4 [Source:NCBI gene;Acc:395904]	5.57852283703177e-34	0,505308209	0.879	0.615	1,36E-29	6	CT
391	ENSGALG00000035016	ID2	inhibitor of DNA binding 2, HLH protein [Source:NCBI gene;Acc:395852]	3.09735059422848e-19	0,492753684	0.717	0.495	7,54E-15	6	CT
451	ENSGALG00000040316	GPC1	glypican 1 [Source:NCBI gene;Acc:424770]	2.15055571706599e-27	0,492670798	0.767	0.509	5,24E-23	6	CT
405	ENSGALG00000036789	ADGRL2	adhesion G protein-coupled receptor L2 [Source:NCBI gene;Acc:424545]	4.43510636771504e-28	0,485033442	0.621	0.341	1,08E-23	6	CT
37	ENSGALG00000003580	MMP2	matrix metallopeptidase 2 [Source:NCBI gene;Acc:386583]	1.28531313229521e-23	0,467002416	0.712	0.458	3,13E-19	6	CT
465	ENSGALG00000041555	COL1A1	collagen type I alpha 1 chain [Source:NCBI gene;Acc:395532]	5.66304298060735e-31	0,449305236	0.988	0.91	1,38E-26	6	CT
419	ENSGALG00000037675	COL14A1	collagen type XIV alpha 1 chain [Source:NCBI gene;Acc:396276]	1.47221579789618e-55	0,429817909	0.262	0.035	3,59E-51	6	CT
233	ENSGALG00000014910	COLEC12	collectin subfamily member 12 [Source:NCBI gene;Acc:421061]	4.87525146981725e-36	0,415290465	0.567	0.233	1,19E-31	6	CT
230	ENSGALG00000014873	GJA1	gap junction protein alpha 1 [Source:NCBI gene;Acc:395278]	1.94765908040887e-06	0,395273681	0.642	0.566	0,047437185	6	CT
444	ENSGALG00000039489	5_8S_rRNA	5.8S ribosomal RNA [Source:RFAM;Acc:RF00002]	3.20435872157931e-14	0,393516543	0.521	0.326	7,80E-10	6	CT

447	ENSGALG00000039985	CDH11	cadherin 11 [Source:NCBI gene;Acc:415797]	1.28754330094399e-16	0,364076304	0.879	0.685	3,14E-12	6	CT
11	ENSGALG00000000839	WNT11	Wnt family member 11 [Source:NCBI gene;Acc:395562]	7.47156243899467e-28	0,353823577	0.367	0.122	1,82E-23	6	CT
325	ENSGALG00000028284	PTX3	pentraxin 3 [Source:NCBI gene;Acc:548626]	1.27560895776013e-26	0,336096079	0.354	0.118	3,11E-22	6	CT
143	ENSGALG00000009302	EMX2	empty spiracles homeobox 2 [Source:NCBI gene;Acc:423923]	1.85906070772315e-35	0,321547189	0.329	0.082	4,53E-31	6	CT
109	ENSGALG00000007804	GXYLT2	glucoside xylosyltransferase 2 [Source:NCBI gene;Acc:416097]	6.9751580410273e-18	0,320925214	0.45	0.229	1,70E-13	6	CT
173	ENSGALG00000010794	MEOX2	mesenchyme homeobox 2 [Source:NCBI gene;Acc:374137]	2.71705948111822e-26	0,319765076	0.383	0.137	6,62E-22	6	CT
179	ENSGALG00000011271	LUM	lumican [Source:NCBI gene;Acc:417891]	2.38465952241863e-13	0,313905678	0.492	0.291	5,81E-09	6	CT
118	ENSGALG00000008298	LRRC17	leucine rich repeat containing 17 [Source:NCBI gene;Acc:395440]	1.37990312658021e-12	0,29830839	0.529	0.331	3,36E-08	6	CT
390	ENSGALG00000034649	TOX3	TOX high mobility group box family member 3 [Source:NCBI gene;Acc:415724]	1.28478960975808e-23	0,295466016	0.338	0.12	3,13E-19	6	CT
273	ENSGALG00000016945	LECT1	leukocyte cell derived chemotaxin 1 [Source:NCBI gene;Acc:395600]	4.72927121208556e-16	0,295458637	0.421	0.215	1,15E-11	6	CT
284	ENSGALG00000017405	NPR3	natriuretic peptide receptor 3 [Source:NCBI gene;Acc:431663]	1.10517217422336e-27	0,293154113	0.212	0.046	2,69E-23	6	CT
395	ENSGALG00000035584	COL3A1	collagen type III alpha 1 chain [Source:NCBI gene;Acc:396340]	1.0142129864419e-22	0,273587262	0.283	0.088	2,47E-18	6	CT
58	ENSGALG00000004304	TCF12	transcription factor 12 [Source:NCBI gene;Acc:396334]	8.25462631013346e-14	0,272957153	0.946	0.895	2,01E-09	6	CT
116	ENSGALG00000008253	TBX5	T-box 5 [Source:NCBI gene;Acc:373988]	5.25226327637687e-10	0,263991763	0.675	0.549	1,28E-05	6	CT
50	ENSGALG00000003923	COL6A3	collagen type VI alpha 3 chain [Source:NCBI gene;Acc:396548]	8.89114128645391e-15	0,257892824	0.312	0.136	2,17E-10	6	CT
268	ENSGALG00000016558	VEGFD	vascular endothelial growth factor D [Source:NCBI gene;Acc:395255]	2.19114706762045e-19	0,256765368	0.288	0.102	5,34E-15	6	CT
329	ENSGALG00000028600	PPIA	peptidylprolyl isomerase A [Source:NCBI gene;Acc:776282]	2.41363945822246e-33	0,430304292	1	0.995	5,88E-29	7	CT
513	ENSGALG00000053871	ENSGALG00000053871		2.11270436873388e-10	0,377699743	0.596	0.427	5,15E-06	7	CT
368	ENSGALG00000032456	COII	Cytochrome c oxidase subunit 2 [Source:UniProtKB/Swiss-Prot;Acc:P18944]	2.22451631356283e-07	0,315158266	0.944	1	0,005418032	7	CT
142	ENSGALG00000009286	ATP5G3	ATP synthase, H+ transporting, mitochondrial Fo complex subunit C3 (subunit 9) [Source:NCBI gene	2.01316104201783e-10	0,308823305	0.932	0.891	4,90E-06	7	CT
218	ENSGALG00000014023	H2AFZ	H2A histone family, member Z [Source:NCBI gene;Acc:426361]	1.30517602545822e-07	0,304429924	0.801	0.73	0,003178887	7	CT
414	ENSGALG00000037167	HIST1H103	histone cluster 1, H1.03 (similar to human histone cluster 1, class H1 genes) [Source:NCBI gene;Acc	2.44961870717891e-112	1,538976076	0.97	0.17	5,97E-108	8	CT
191	ENSGALG00000011747	ENSGALG00000011747		3.90778036176664e-140	1,242596128	0.919	0.106	9,52E-136	8	CT
250	ENSGALG00000015691	SMC2	structural maintenance of chromosomes 2 [Source:NCBI gene;Acc:396156]	2.78415102718536e-53	1,03011509	0.99	0.372	6,78E-49	8	CT
49	ENSGALG00000003922	TOP2A	topoisomerase (DNA) II alpha [Source:NCBI gene;Acc:395570]	1.59871799073007e-45	0,833865368	0.929	0.329	3,89E-41	8	CT
301	ENSGALG00000025996	ki-67	marker of proliferation Ki-67 [Source:NCBI gene;Acc:423963]	3.60665693541915e-82	0,758517953	0.899	0.163	8,78E-78	8	CT
502	ENSGALG00000050309	HIST1H2BO	histone cluster 1, H2bo [Source:NCBI gene;Acc:100858607]	9.99449555968318e-162	0,755052166	0.747	0.05	2,43E-157	8	CT
507	ENSGALG00000052192	TUBA1B	tubulin alpha-1A chain-like [Source:NCBI gene;Acc:100859737]	2.90011397800522e-32	0,670162453	1	0.819	7,06E-28	8	CT
336	ENSGALG00000029292	PCNA	proliferating cell nuclear antigen [Source:NCBI gene;Acc:373984]	2.17089691677275e-31	0,65588629	0.96	0.491	5,29E-27	8	CT
264	ENSGALG00000016442	RRM2	ribonucleotide reductase regulatory subunit M2 [Source:NCBI gene;Acc:421936]	2.01938255761334e-47	0,577514733	0.778	0.187	4,92E-43	8	CT
377	ENSGALG00000033212	HSP90AA1	heat shock protein 90 alpha family class A member 1 [Source:NCBI gene;Acc:423463]	1.69406495655021e-24	0,514370211	1	0.843	4,13E-20	8	CT
76	ENSGALG00000005961	DGUOK	deoxyguanosine kinase [Source:NCBI gene;Acc:770922]	1.31107774049715e-51	0,500941827	0.788	0.18	3,19E-47	8	CT
29	ENSGALG00000003324	PRRX1	paired related homeobox 1 [Source:NCBI gene;Acc:373941]	1.27992750268781e-18	0,495269029	0.96	0.595	3,12E-14	8	CT
360	ENSGALG00000032039	HOXD13	homeobox D13 [Source:NCBI gene;Acc:396415]	5.21507110700105e-29	0,472938489	0.798	0.272	1,27E-24	8	CT
164	ENSGALG00000010255	NASP	nuclear autoantigenic sperm protein [Source:NCBI gene;Acc:424600]	1.03712983440332e-20	0,467685706	0.929	0.587	2,53E-16	8	CT
383	ENSGALG00000034168	WNT5A	Wnt family member 5A [Source:NCBI gene;Acc:395703]	2.29313089179447e-21	0,446471659	0.909	0.435	5,59E-17	8	CT
270	ENSGALG00000016582	PBK	PDZ binding kinase [Source:NCBI gene;Acc:422003]	5.36426531217028e-34	0,441987212	0.697	0.194	1,31E-29	8	CT
469	ENSGALG00000041640	TWIST1	twist family bHLH transcription factor 1 [Source:NCBI gene;Acc:395491]	1.8167291782415e-21	0,424805808	0.97	0.548	4,42E-17	8	CT
147	ENSGALG00000009553	SMC4	structural maintenance of chromosomes 4 [Source:NCBI gene;Acc:395189]	2.29214626254828e-45	0,395303874	0.758	0.174	5,58E-41	8	CT
403	ENSGALG00000036645	KIF15	kinesin family member 15 [Source:NCBI gene;Acc:420708]	7.06461494656276e-44	0,390474882	0.677	0.144	1,72E-39	8	CT
106	ENSGALG00000007537	INCENP	inner centromere protein [Source:NCBI gene;Acc:396270]	2.27161042254148e-37	0,386583268	0.646	0.147	5,53E-33	8	CT
217	ENSGALG00000014023	H2AFZ	H2A histone family, member Z [Source:NCBI gene;Acc:426361]	1.02329821875207e-15	0,382167251	0.98	0.726	2,49E-11	8	CT
87	ENSGALG00000006512	HSPA8	heat shock 70kDa protein 8 [Source:NCBI gene;Acc:395853]	4.40664348890506e-13	0,371026763	0.99	0.81	1,07E-08	8	CT
457	ENSGALG00000040586	TUBA3E	tubulin, alpha 3e [Source:NCBI gene;Acc:421169]	3.5945456411681e-23	0,364618231	0.727	0.258	8,75E-19	8	CT
386	ENSGALG00000034271	UHRF1	ubiquitin like with PHD and ring finger domains 1 [Source:NCBI gene;Acc:420154]	1.91975078420519e-21	0,360507089	0.727	0.284	4,68E-17	8	CT
237	ENSGALG00000015013	MSX1	msh homeobox 1 [Source:NCBI gene;Acc:396484]	2.27810413474901e-23	0,36039911	0.717	0.238	5,55E-19	8	CT
276	ENSGALG00000017025	CKAP2	cytoskeleton associated protein 2 [Source:NCBI gene;Acc:418882]	8.13917136421424e-24	0,354663706	0.758	0.259	1,98E-19	8	CT
124	ENSGALG00000008584	NUSAP1	nucleolar and spindle associated protein 1 [Source:NCBI gene;Acc:423213]	1.58293110448118e-49	0,352725614	0.657	0.118	3,86E-45	8	CT
501	ENSGALG00000049408	HIST1H2B8	histone cluster 1, H2B-VIII [Source:NCBI gene;Acc:427886]	3.94140574790261e-86	0,345015822	0.505	0.042	9,60E-82	8	CT
434	ENSGALG00000038848	MSX2	msh homeobox 2 [Source:NCBI gene;Acc:395245]	9.22025407135505e-20	0,32517038	0.626	0.205	2,25E-15	8	CT
210	ENSGALG00000013208	CENPE	centromere protein E [Source:NCBI gene;Acc:422716]	3.33455388192321e-35	0,322192421	0.667	0.154	8,12E-31	8	CT
155	ENSGALG00000009690	CENPF	centromere protein F [Source:NCBI gene;Acc:395357]	1.74983642972275e-26	0,318413988	0.616	0.167	4,26E-22	8	CT
21	ENSGALG00000002781	BRCA1	BRCA1, DNA repair associated [Source:NCBI gene;Acc:373983]	4.01840731792959e-39	0,317176117	0.535	0.104	9,79E-35	8	CT
298	ENSGALG00000025759	TFDP2	transcription factor Dp-2 [Source:NCBI gene;Acc:424780]	2.73040503758857e-16	0,31530457	0.778	0.349	6,65E-12	8	CT
478	ENSGALG00000042875	HMGB1	high mobility group box 1 [Source:NCBI gene;Acc:395724]	1.98091810259582e-13	0,308378908	1	0.941	4,82E-09	8	CT

197	ENSGALG00000012684	DEK	DEK proto-oncogene [Source:NCBI gene;Acc:420828]	1.75012075363387e-12	0,307934867	0.798	0.458	4,26E-08	8	CT
172	ENSGALG00000010745	HMGB2	high mobility group box 2 [Source:NCBI gene;Acc:396482]	3.08504319661584e-15	0,304284024	0.96	0.545	7,51E-11	8	CT
514	ENSGALG00000053871	ENSGALG00000053871		1.83129830527408e-17	0,302504539	0.909	0.42	4,46E-13	8	CT
176	ENSGALG00000010900	SPC25	SPC25, NDC80 kinetochore complex component [Source:NCBI gene;Acc:424172]	1.79464309055209e-26	0,296672308	0.596	0.164	4,37E-22	8	CT
425	ENSGALG00000037944	LNPK	lunapark, ER junction formation factor [Source:NCBI gene;Acc:424141]	2.09086584902337e-15	0,296576669	0.626	0.258	5,09E-11	8	CT
212	ENSGALG00000013607	FBXO5	F-box protein 5 [Source:NCBI gene;Acc:421642]	1.26602236479338e-38	0,296502786	0.545	0.103	3,08E-34	8	CT
367	ENSGALG00000032398	ASPM	abnormal spindle microtubule assembly [Source:NCBI gene;Acc:424354]	1.21066384826278e-26	0,295203002	0.677	0.192	2,95E-22	8	CT
228	ENSGALG00000014801	NDC80	NDC80, kinetochore complex component [Source:NCBI gene;Acc:395134]	9.14776912814554e-36	0,293646985	0.586	0.125	2,23E-31	8	CT
141	ENSGALG00000009286	ATP5G3	ATP synthase, H+ transporting, mitochondrial Fo complex subunit C3 (subunit 9) [Source:NCBI gene	2.93201177889899e-11	0,287708844	0.99	0.89	7,14E-07	8	CT
281	ENSGALG00000017350	RRM1	ribonucleotide reductase catalytic subunit M1 [Source:NCBI gene;Acc:419081]	1.78545334323782e-21	0,284539547	0.626	0.207	4,35E-17	8	CT
186	ENSGALG00000011504	TMPO	thymopoietin [Source:NCBI gene;Acc:417923]	1.58361289913093e-17	0,283498111	0.758	0.3	3,86E-13	8	CT
53	ENSGALG00000004195	KIF4B	kinesin family member 4B [Source:NCBI gene;Acc:395823]	4.02644242893159e-22	0,282705725	0.697	0.233	9,81E-18	8	CT
82	ENSGALG00000006267	TPX2	TPX2, microtubule nucleation factor [Source:NCBI gene;Acc:395081]	2.86432166360483e-18	0,272961872	0.687	0.248	6,98E-14	8	CT
227	ENSGALG00000014753	CENPK	centromere protein K [Source:NCBI gene;Acc:427162]	2.86423677825627e-30	0,26897343	0.606	0.15	6,98E-26	8	CT
222	ENSGALG00000014513	CDCA3	cell division cycle associated 3 [Source:NCBI gene;Acc:770517]	5.43203882872694e-24	0,267824289	0.545	0.148	1,32E-19	8	CT
32	ENSGALG00000003438	NUF2	NUF2, NDC80 kinetochore complex component [Source:NCBI gene;Acc:395135]	2.17302739051152e-27	0,263559041	0.566	0.145	5,29E-23	8	CT
144	ENSGALG00000009305	LBR	lamin B receptor [Source:NCBI gene;Acc:396285]	3.54923355943842e-14	0,26118611	0.758	0.359	8,64E-10	8	CT
363	ENSGALG00000032207	HMGN4	high mobility group nucleosomal binding domain 4 [Source:NCBI gene;Acc:768675]	2.75511601918131e-09	0,25902529	1	0.887	6,71E-05	8	CT
99	ENSGALG00000007191	TK1	thymidine kinase 1 [Source:NCBI gene;Acc:395719]	9.60559627431482e-27	0,257088569	0.444	0.1	2,34E-22	8	CT
133	ENSGALG00000008957	MSH6	mutS homolog 6 [Source:NCBI gene;Acc:421291]	1.36432794812436e-17	0,254275206	0.576	0.202	3,32E-13	8	CT
84	ENSGALG00000006300	LDHA	lactate dehydrogenase A [Source:NCBI gene;Acc:396221]	9.34863027316635e-12	0,253624884	0.556	0.229	2,28E-07	8	CT
19	ENSGALG00000002569	RAN	RAN, member RAS oncogene family [Source:NCBI gene;Acc:396193]	6.13033843705971e-08	0,251198075	0.949	0.675	0,001493105	8	CT

Supplementary Table 1

Markers for the CT and muscle clusters at E10

List of CT and muscle markers associated to the CT and muscle clusters at E10.

In the “Cluster type” column, “CT” or “M” means that the cluster respectively has a CT or a M identity.

	ensembl_gene_id	external_gene_name	description	p_val	avg_logFC	pct.1	pct.2	p_val_adj	cluster	cluster type
454	ENSGALG00000027887	C1QTNF3	C1q and tumor necrosis factor related protein 3 [Source:NCBI gene;Acc:427430]	0	1,186584821	0.878	0.065	0	0	M
584	ENSGALG00000036798	COL4A1	collagen type IV alpha 1 chain [Source:NCBI gene;Acc:395530]	0	0,590801326	0.754	0.196	0	0	M
403	ENSGALG00000017644	COTL1	coactosin like F-actin binding protein 1 [Source:NCBI gene;Acc:768420]	0	0,551157738	0.767	0.287	0	0	M
78	ENSGALG00000003642	PDGFA	platelet derived growth factor subunit A [Source:NCBI gene;Acc:374196]	0	0,543580599	0.594	0.091	0	0	M
357	ENSGALG00000015624	VCAN	versican [Source:NCBI gene;Acc:395565]	0	0,439751146	0.844	0.556	0	0	M
491	ENSGALG00000029921	CSRP2	cysteine and glycine rich protein 2 [Source:NCBI gene;Acc:396128]	0	0,425891399	0.545	0.139	0	0	M
470	ENSGALG00000028543	FGFR4	fibroblast growth factor receptor 4 [Source:NCBI gene;Acc:395603]	0	0,406863439	0.496	0.075	0	0	M
721	ENSGALG00000052192	TUBA1B	tubulin alpha-1A chain-like [Source:NCBI gene;Acc:100859737]	0	0,399268216	0.87	0.649	0	0	M
683	ENSGALG00000043204	PAX7	paired box 7 [Source:NCBI gene;Acc:395942]	0	0,376913299	0.405	0.012	0	0	M
246	ENSGALG00000010745	HMGB2	high mobility group box 2 [Source:NCBI gene;Acc:396482]	2.63767616225805e-272	0,322993335	0.585	0.301	6,42E-268	0	M
439	ENSGALG00000026383	TMSB4X	thymosin beta 4, X-linked [Source:NCBI gene;Acc:408047]	1.52415553274396e-243	0,302090125	0.974	0.881	3,71E-239	0	M
670	ENSGALG00000042492	PITX2	paired like homeodomain 2 [Source:NCBI gene;Acc:395862]	0	0,292121867	0.319	0.039	0	0	M
371	ENSGALG00000016281	DMD	dystrophin [Source:NCBI gene;Acc:396236]	0	0,291681443	0.401	0.093	0	0	M
481	ENSGALG00000029401	SIX1	SIX homeobox 1 [Source:NCBI gene;Acc:693262]	0	0,280881621	0.442	0.127	0	0	M
500	ENSGALG00000030878	RPLP1	ribosomal protein lateral stalk subunit P1 [Source:NCBI gene;Acc:396262]	0	0,262673409	1	0.998	0	0	M
282	ENSGALG00000011738	ARHGDIB	Rho GDP dissociation inhibitor beta [Source:NCBI gene;Acc:417941]	0	0,26132338	0.371	0.099	0	0	M
562	ENSGALG00000035584	COL3A1	collagen type III alpha 1 chain [Source:NCBI gene;Acc:396340]	0	0,775868627	0.916	0.47	0	1	CT
56	ENSGALG00000003193	CRABP-I	cellular retinoic acid binding protein 1 [Source:NCBI gene;Acc:374211]	2.37043312507191e-197	0,678389798	0.494	0.262	5,77E-193	1	CT
586	ENSGALG00000036847	PLAC9	placenta specific 9 [Source:NCBI gene;Acc:423636]	0	0,675178658	0.528	0.075	0	1	CT
212	ENSGALG00000009241	SFRP2	secreted frizzled related protein 2 [Source:NCBI gene;Acc:395546]	1.69590252518006e-270	0,663279209	0.489	0.228	4,13E-266	1	CT
198	ENSGALG00000008677	VIM	vimentin [Source:NCBI gene;Acc:420519]	0	0,658860987	0.86	0.698	0	1	CT
40	ENSGALG00000002546	COL5A1	collagen type V alpha 1 chain [Source:NCBI gene;Acc:395568]	0	0,631430995	0.969	0.68	0	1	CT
345	ENSGALG00000015193	CCDC80	coiled-coil domain containing 80 [Source:NCBI gene;Acc:395074]	0	0,606500063	0.68	0.322	0	1	CT
317	ENSGALG00000014233	FBLN1	fibulin 1 [Source:NCBI gene;Acc:373979]	0	0,572718909	0.505	0.166	0	1	CT
260	ENSGALG00000011271	LUM	lumican [Source:NCBI gene;Acc:417891]	0	0,54122746	0.521	0.175	0	1	CT
185	ENSGALG00000008298	LRRC17	leucine rich repeat containing 17 [Source:NCBI gene;Acc:395440]	0	0,526744579	0.618	0.278	0	1	CT
264	ENSGALG00000011274	DCN	decorin [Source:NCBI gene;Acc:417892]	0	0,524120421	0.924	0.669	0	1	CT
69	ENSGALG00000003578	FN1	fibronectin 1 [Source:NCBI gene;Acc:396133]	0	0,517228904	0.777	0.488	0	1	CT
275	ENSGALG00000011687	AHNAK2	AHNAK nucleoprotein 2 [Source:NCBI gene;Acc:100859120]	0	0,509613079	0.463	0.145	0	1	CT
573	ENSGALG00000036240	AKAP12	A-kinase anchoring protein 12 [Source:NCBI gene;Acc:421634]	4.10115367641829e-206	0,481842937	0.704	0.502	9,99E-202	1	CT
455	ENSGALG00000027891	NREP	neuronal regeneration related protein [Source:NCBI gene;Acc:396353]	3.47752384997795e-170	0,468868623	0.706	0.534	8,47E-166	1	CT
195	ENSGALG00000008542	ADD3	adducin 3 (gamma) [Source:NCBI gene;Acc:373892]	3.12891665405024e-275	0,403253604	0.507	0.233	7,62E-271	1	CT
696	ENSGALG00000043690	ECRG4	chromosome 1 open reading frame, human C2orf40 [Source:NCBI gene;Acc:771055]	2.36586944558512e-247	0,3960296	0.614	0.341	5,76E-243	1	CT
337	ENSGALG00000014994	CRHBP	corticotropin releasing hormone binding protein [Source:NCBI gene;Acc:427214]	1.42987974151927e-148	0,388072488	0.168	0.047	3,48E-144	1	CT
589	ENSGALG00000037164	ZFHX4	zinc finger homeobox 4 [Source:NCBI gene;Acc:395904]	5.37961543353908e-283	0,382983472	0.515	0.233	1,31E-278	1	CT
257	ENSGALG00000011270	KERA	keratocan [Source:NCBI gene;Acc:373995]	0	0,358269148	0.409	0.122	0	1	CT
708	ENSGALG00000047320	MFAP5	microfibrillar associated protein 5 [Source:NCBI gene;Acc:418256]	0	0,328249706	0.248	0.032	0	1	CT
20	ENSGALG00000001177	CD34	CD34 molecule [Source:NCBI gene;Acc:419856]	0	0,322427631	0.282	0.049	0	1	CT
460	ENSGALG00000028041	DKK2	dickkopf WNT signaling pathway inhibitor 2 [Source:NCBI gene;Acc:422531]	1.13054107659442e-300	0,316106159	0.285	0.072	2,75E-296	1	CT
334	ENSGALG00000014970	FSTL1	follistatin like 1 [Source:NCBI gene;Acc:395349]	9.01626942747278e-124	0,305339965	0.631	0.461	2,20E-119	1	CT
443	ENSGALG00000026736	OGN	osteoglycin [Source:NCBI gene;Acc:374039]	1.21400892937088e-231	0,291614707	0.378	0.145	2,96E-227	1	CT
339	ENSGALG00000014999	MAP1B	microtubule associated protein 1B [Source:NCBI gene;Acc:396174]	7.01295142739643e-220	0,288840053	0.321	0.113	1,71E-215	1	CT
627	ENSGALG00000039985	CDH11	cadherin 11 [Source:NCBI gene;Acc:415797]	1.43432534398335e-150	0,28712614	0.709	0.456	3,49E-146	1	CT
73	ENSGALG00000003580	MMP2	matrix metallopeptidase 2 [Source:NCBI gene;Acc:386583]	5.47723964296297e-97	0,281943807	0.503	0.332	1,33E-92	1	CT
318	ENSGALG00000014261	UCHL1	ubiquitin C-terminal hydrolase L1 [Source:NCBI gene;Acc:770302]	2.37931566937163e-166	0,280377568	0.326	0.139	5,80E-162	1	CT
425	ENSGALG00000025862	TMEM26	transmembrane protein 26 [Source:NCBI gene;Acc:423663]	0	0,279672591	0.248	0.038	0	1	CT
557	ENSGALG00000035016	ID2	inhibitor of DNA binding 2, HLH protein [Source:NCBI gene;Acc:395852]	7.77427552625433e-125	0,279491649	0.395	0.217	1,89E-120	1	CT
93	ENSGALG00000003939	KLF2	Kruppel like factor 2 [Source:NCBI gene;Acc:420148]	2.93390303763254e-185	0,264629722	0.245	0.079	7,15E-181	1	CT

546	ENSGALG00000033880	CRISPLD1	cysteine rich secretory protein LCCL domain containing 1 [Source:NCBI gene;Acc:395369]	1.66271095955925e-180	0,264012292	0.23	0.072	4,05E-176	1	CT
15	ENSGALG00000000839	WNT11	Wnt family member 11 [Source:NCBI gene;Acc:395562]	4.28099965568366e-254	0,255170565	0.251	0.065	1,04E-249	1	CT
363	ENSGALG00000015720	CHODL	chondrolectin [Source:NCBI gene;Acc:418474]	1.85353718208544e-80	0,253398214	0.244	0.122	4,51E-76	1	CT
701	ENSGALG00000045548	ENSGALG00000045548		5.80519548734147e-210	0,252827489	0.271	0.086	1,41E-205	1	CT
219	ENSGALG00000009612	TGFB2	transforming growth factor beta 2 [Source:NCBI gene;Acc:421352]	3.16544716108435e-93	0,25221265	0.508	0.338	7,71E-89	1	CT
572	ENSGALG00000036240	AKAP12	A-kinase anchoring protein 12 [Source:NCBI gene;Acc:421634]	0	0,847907466	0.862	0.482	0	2	CT
564	ENSGALG00000035584	COL3A1	collagen type III alpha 1 chain [Source:NCBI gene;Acc:396340]	0	0,826082938	0.942	0.471	0	2	CT
262	ENSGALG00000011274	DCN	decorin [Source:NCBI gene;Acc:417892]	0	0,663705001	0.967	0.666	0	2	CT
97	ENSGALG00000004270	ALDH1A2	aldehyde dehydrogenase 1 family member A2 [Source:NCBI gene;Acc:395844]	0	0,662546767	0.518	0.042	0	2	CT
203	ENSGALG00000008883	TCF7L2	transcription factor 7-like 2 (T-cell specific, HMG-box) [Source:NCBI gene;Acc:395508]	0	0,603111698	0.749	0.42	0	2	CT
241	ENSGALG00000010461	EBF3	early B cell factor 3 [Source:NCBI gene;Acc:395175]	0	0,570307148	0.711	0.3	0	2	CT
361	ENSGALG00000015708	FGFR3	fibroblast growth factor receptor 3 [Source:NCBI gene;Acc:396515]	0	0,529666044	0.33	0.09	0	2	CT
220	ENSGALG00000009612	TGFB2	transforming growth factor beta 2 [Source:NCBI gene;Acc:421352]	0	0,517672467	0.666	0.318	0	2	CT
382	ENSGALG00000016584	EGFL6	EGF like domain multiple 6 [Source:HGNC Symbol;Acc:HGNC:3235]	0	0,499531142	0.44	0.051	0	2	CT
147	ENSGALG00000006583	LSP1P1	lymphocyte-specific protein 1 pseudogene 1 [Source:NCBI gene;Acc:374254]	0	0,474272156	0.537	0.179	0	2	CT
199	ENSGALG00000008677	VIM	vimentin [Source:NCBI gene;Acc:420519]	1.7767571439429e-285	0,464183939	0.869	0.699	4,33E-281	2	CT
41	ENSGALG00000002546	COL5A1	collagen type V alpha 1 chain [Source:NCBI gene;Acc:395568]	0	0,461548201	0.953	0.685	0	2	CT
358	ENSGALG00000015624	VCAN	versican [Source:NCBI gene;Acc:395565]	1.63401134000992e-240	0,460397801	0.826	0.585	3,98E-236	2	CT
663	ENSGALG00000042077	HIC1	HIC ZBTB transcriptional repressor 1 [Source:NCBI gene;Acc:396164]	0	0,448636334	0.517	0.161	0	2	CT
536	ENSGALG00000033365	ALDH1A3	aldehyde dehydrogenase 1 family member A3 [Source:NCBI gene;Acc:395389]	0	0,414953684	0.357	0.061	0	2	CT
394	ENSGALG00000017036	LHFPL6	lipoma HMGIC fusion partner-like 1 [Source:NCBI gene;Acc:418889]	0	0,413434604	0.507	0.198	0	2	CT
420	ENSGALG00000024488	CD99	CD99 molecule (Xg blood group) [Source:NCBI gene;Acc:418662]	0	0,397783047	0.499	0.191	0	2	CT
331	ENSGALG00000014873	GJA1	gap junction protein alpha 1 [Source:NCBI gene;Acc:395278]	6.47046480085907e-187	0,366206897	0.585	0.322	1,58E-182	2	CT
90	ENSGALG00000003923	COL6A3	collagen type VI alpha 3 chain [Source:NCBI gene;Acc:396548]	5.32665496237511e-215	0,363448163	0.457	0.204	1,30E-210	2	CT
307	ENSGALG00000012834	AKR1D1	aldo-keto reductase family 1 member D1 [Source:NCBI gene;Acc:418107]	8.68168950947482e-281	0,349761206	0.449	0.166	2,11E-276	2	CT
196	ENSGALG00000008542	ADD3	adducin 3 (gamma) [Source:NCBI gene;Acc:373892]	3.93775215653578e-250	0,341068456	0.513	0.235	9,59E-246	2	CT
389	ENSGALG00000016906	SPRY2	sprouty RTK signaling antagonist 2 [Source:NCBI gene;Acc:395584]	0	0,333379303	0.369	0.117	0	2	CT
6	ENSGALG00000000242	EBF2	early B-cell factor 2 [Source:NCBI gene;Acc:395176]	0	0,331137399	0.373	0.086	0	2	CT
258	ENSGALG00000011270	KERA	keratocan [Source:NCBI gene;Acc:373995]	5.50098101739202e-206	0,330359671	0.354	0.133	1,34E-201	2	CT
652	ENSGALG00000041621	LY6E	lymphocyte antigen 6 family member E [Source:NCBI gene;Acc:395550]	7.15250303561479e-206	0,321313875	0.381	0.161	1,74E-201	2	CT
129	ENSGALG00000006080	GPC4	glypican 4 [Source:NCBI gene;Acc:422234]	9.16577259812587e-255	0,318448495	0.422	0.164	2,23E-250	2	CT
84	ENSGALG00000003733	LIFR	leukemia inhibitory factor receptor alpha [Source:NCBI gene;Acc:395262]	7.90103962753004e-196	0,311118752	0.414	0.189	1,92E-191	2	CT
617	ENSGALG00000039216	COL6A2	collagen type VI alpha 2 chain [Source:NCBI gene;Acc:396292]	6.01599879174399e-172	0,307824952	0.495	0.255	1,47E-167	2	CT
11	ENSGALG00000000690	GDPD4	glycerophosphodiester phosphodiesterase domain containing 4 [Source:NCBI gene;Acc:419084]	0	0,302459972	0.269	0.021	0	2	CT
526	ENSGALG00000032687	PHLDA2	pleckstrin homology like domain family A member 2 [Source:NCBI gene;Acc:423088]	4.26422603894384e-235	0,301031212	0.394	0.147	1,04E-230	2	CT
232	ENSGALG00000010013	EDNRA	endothelin receptor type A [Source:NCBI gene;Acc:373908]	0	0,283266242	0.307	0.075	0	2	CT
45	ENSGALG00000002763	FRZB	frizzled related protein [Source:NCBI gene;Acc:395545]	1.29253396532876e-220	0,276868931	0.329	0.115	3,15E-216	2	CT
353	ENSGALG00000015403	EPHA3	EPH receptor A3 [Source:NCBI gene;Acc:396402]	1.55247303423749e-234	0,26840896	0.248	0.065	3,78E-230	2	CT
236	ENSGALG00000010044	KCNMB4	potassium calcium-activated channel subfamily M regulatory beta subunit 4 [Source:NCBI gene;Acc	0	0,257697981	0.223	0.028	0	2	CT
397	ENSGALG00000017046	POSTN	periostin [Source:NCBI gene;Acc:395429]	0	1,132627114	0.785	0.292	0	3	CT
100	ENSGALG00000004274	TWIST2	twist family bHLH transcription factor 2 [Source:NCBI gene;Acc:395405]	0	0,699888836	0.759	0.158	0	3	CT
654	ENSGALG00000041640	TWIST1	twist family bHLH transcription factor 1 [Source:NCBI gene;Acc:395491]	0	0,682566698	0.767	0.182	0	3	CT
650	ENSGALG00000041555	COL1A1	collagen type I alpha 1 chain [Source:NCBI gene;Acc:395532]	0	0,677492573	0.998	0.775	0	3	CT
508	ENSGALG00000031424	SDC2	syndecan 2 [Source:NCBI gene;Acc:374102]	0	0,557822334	0.745	0.31	0	3	CT
329	ENSGALG00000014873	GJA1	gap junction protein alpha 1 [Source:NCBI gene;Acc:395278]	0	0,525914763	0.751	0.314	0	3	CT
438	ENSGALG00000026383	TMSB4X	thymosin beta 4, X-linked [Source:NCBI gene;Acc:408047]	0	0,506399133	0.996	0.89	0	3	CT
154	ENSGALG00000006821	TNMD	tenomodulin [Source:NCBI gene;Acc:404530]	0	0,495531548	0.571	0.102	0	3	CT
645	ENSGALG00000041140	HTRA1	HtrA serine peptidase 1 [Source:NCBI gene;Acc:100857572]	0	0,484925275	0.519	0.063	0	3	CT
618	ENSGALG00000039216	COL6A2	collagen type VI alpha 2 chain [Source:NCBI gene;Acc:396292]	0	0,475468	0.68	0.244	0	3	CT
551	ENSGALG00000034326	TMEM158	transmembrane protein 158 [Source:NCBI gene;Acc:776186]	0	0,437643915	0.427	0.014	0	3	CT
525	ENSGALG00000032687	PHLDA2	pleckstrin homology like domain family A member 2 [Source:NCBI gene;Acc:423088]	4.23577597026088e-195	0,435166245	0.408	0.153	1,03E-190	3	CT
688	ENSGALG00000043329	PTMA	prothymosin, alpha [Source:NCBI gene;Acc:424931]	3.05678827613447e-229	0,379432607	0.979	0.901	7,45E-225	3	CT
139	ENSGALG00000006346	CXCL14	C-X-C motif chemokine ligand 14 [Source:NCBI gene;Acc:395451]	0	0,335201066	0.446	0.089	0	3	CT
306	ENSGALG00000012821	TUBB2B	tubulin beta 2B class IIb [Source:NCBI gene;Acc:420883]	4.14415790747645e-220	0,331240003	0.621	0.302	1,01E-215	3	CT
660	ENSGALG00000041993	UGDH	UDP-glucose 6-dehydrogenase [Source:NCBI gene;Acc:422792]	4.10679804167505e-155	0,324283864	0.581	0.313	1,00E-150	3	CT

628	ENSGALG00000039985	CDH11	cadherin 11 [Source:NCBI gene;Acc:415797]	4.30391866976874e-196	0,314793863	0.823	0.455	1,05E-191	3	CT
464	ENSGALG00000028354	METRNL	meteorin, glial cell differentiation regulator-like [Source:NCBI gene;Acc:417332]	6.00879778537942e-293	0,311141075	0.522	0.178	1,46E-288	3	CT
734	ENSGALG00000054048	ENSGALG0000005404	transmembrane protein 211-like [Source:NCBI gene;Acc:101749175]	0	0,294612393	0.362	0.049	0	3	CT
603	ENSGALG00000037953	TUBA1A	tubulin alpha 1a [Source:NCBI gene;Acc:429035]	8.4074803318307e-148	0,288493864	0.755	0.483	2,05E-143	3	CT
36	ENSGALG00000002515	OLFM1	olfactomedin 1 [Source:NCBI gene;Acc:395535]	2.278058207992e-241	0,284985717	0.464	0.161	5,55E-237	3	CT
558	ENSGALG00000035184	SDC3	syndecan 3 [Source:NCBI gene;Acc:396343]	0	0,283875157	0.378	0.073	0	3	CT
675	ENSGALG00000042875	HMGB1	high mobility group box 1 [Source:NCBI gene;Acc:395724]	1.8316902460195e-89	0,277240977	0.913	0.783	4,46E-85	3	CT
623	ENSGALG00000039705	GFPT2	glutamine-fructose-6-phosphate transaminase 2 [Source:NCBI gene;Acc:426962]	0	0,273216789	0.319	0.061	0	3	CT
632	ENSGALG00000040220	TWIST3	Twist homlog 3 [Source:NCBI gene;Acc:426886]	0	0,27301075	0.331	0.022	0	3	CT
25	ENSGALG00000001472	JAM3	junctional adhesion molecule 3 [Source:NCBI gene;Acc:419736]	2.00391494514339e-180	0,268744799	0.528	0.24	4,88E-176	3	CT
169	ENSGALG00000007361	SST	somatostatin [Source:NCBI gene;Acc:396279]	0	1,373185251	0.42	0.012	0	4	CT
330	ENSGALG00000014873	GJA1	gap junction protein alpha 1 [Source:NCBI gene;Acc:395278]	0	0,976744291	0.867	0.311	0	4	CT
655	ENSGALG00000041640	TWIST1	twist family bHLH transcription factor 1 [Source:NCBI gene;Acc:395491]	0	0,717631737	0.78	0.19	0	4	CT
99	ENSGALG00000004274	TWIST2	twist family bHLH transcription factor 2 [Source:NCBI gene;Acc:395405]	0	0,592629789	0.686	0.173	0	4	CT
43	ENSGALG00000002606	CLSTN1	calsyntenin 1 [Source:NCBI gene;Acc:395372]	1.59781309929061e-303	0,49179062	0.548	0.196	3,89E-299	4	CT
639	ENSGALG00000040465	ZEB2	zinc finger E-box binding homeobox 2 [Source:NCBI gene;Acc:424306]	0	0,47740424	0.641	0.228	0	4	CT
687	ENSGALG00000043329	PTMA	prothymosin, alpha [Source:NCBI gene;Acc:424931]	1.25226115389366e-267	0,439970365	0.985	0.902	3,05E-263	4	CT
26	ENSGALG00000001640	RNF122	ring finger protein 122 [Source:NCBI gene;Acc:426922]	0	0,384424414	0.47	0.076	0	4	CT
62	ENSGALG00000003324	PRRX1	paired related homeobox 1 [Source:NCBI gene;Acc:373941]	3.52169321556187e-165	0,36207705	0.518	0.233	8,58E-161	4	CT
249	ENSGALG00000010903	RASGEF1B	RasGEF domain family member 1B [Source:NCBI gene;Acc:100859744]	0	0,338207508	0.324	0.036	0	4	CT
518	ENSGALG00000031862	BCL11B	B-cell CLL/lymphoma 11B [Source:NCBI gene;Acc:423444]	0	0,328421845	0.379	0.038	0	4	CT
285	ENSGALG00000012017	DAAM1	dishevelled associated activator of morphogenesis 1 [Source:NCBI gene;Acc:423532]	7.2557999856428e-153	0,291070632	0.467	0.204	1,77E-148	4	CT
360	ENSGALG00000015708	FGFR3	fibroblast growth factor receptor 3 [Source:NCBI gene;Acc:396515]	1.35451934436729e-228	0,288909408	0.363	0.099	3,30E-224	4	CT
174	ENSGALG00000007582	DPYSL3	dihydropyrimidinase like 3 [Source:NCBI gene;Acc:395154]	6.85340923610227e-120	0,286828802	0.429	0.199	1,67E-115	4	CT
643	ENSGALG00000040493	PTN	pleiotrophin [Source:NCBI gene;Acc:418125]	4.05918311681579e-109	0,753929003	0.416	0.206	9,89E-105	5	CT
704	ENSGALG00000046616	CHGB	chromogranin B [Source:NCBI gene;Acc:421312]	0	0,611985778	0.265	0.02	0	5	CT
58	ENSGALG00000003193	CRABP-I	cellular retinoic acid binding protein 1 [Source:NCBI gene;Acc:374211]	4.6025500204062e-101	0,602451265	0.484	0.277	1,12E-96	5	CT
365	ENSGALG00000015908	COL12A1	collagen type XII alpha 1 chain [Source:NCBI gene;Acc:395875]	6.12352849813804e-242	0,52537705	0.633	0.27	1,49E-237	5	CT
38	ENSGALG00000002546	COL5A1	collagen type V alpha 1 chain [Source:NCBI gene;Acc:395568]	6.22289012063526e-213	0,518590549	0.943	0.699	1,52E-208	5	CT
243	ENSGALG00000010461	EBF3	early B cell factor 3 [Source:NCBI gene;Acc:395175]	7.25826012701489e-201	0,485665772	0.637	0.326	1,77E-196	5	CT
74	ENSGALG00000003580	MMP2	matrix metallopeptidase 2 [Source:NCBI gene;Acc:386583]	9.36901230246775e-147	0,483810997	0.588	0.335	2,28E-142	5	CT
383	ENSGALG00000016635	MXRA5	matrix remodeling associated 5 [Source:NCBI gene;Acc:418657]	2.67745348116708e-232	0,440067082	0.456	0.16	6,52E-228	5	CT
415	ENSGALG00000023904	FIBIN	fin bud initiation factor homolog (zebrafish) [Source:NCBI gene;Acc:426933]	8.38323548234377e-190	0,435024471	0.441	0.171	2,04E-185	5	CT
346	ENSGALG00000015193	CCDC80	coiled-coil domain containing 80 [Source:NCBI gene;Acc:395074]	8.47784990865458e-143	0,431677281	0.612	0.348	2,06E-138	5	CT
625	ENSGALG00000039985	CDH11	cadherin 11 [Source:NCBI gene;Acc:415797]	3.509167698872e-166	0,428237232	0.767	0.466	8,55E-162	5	CT
211	ENSGALG00000009241	SFRP2	secreted frizzled related protein 2 [Source:NCBI gene;Acc:395546]	3.16760888380799e-86	0,427186638	0.438	0.247	7,72E-82	5	CT
483	ENSGALG00000029429	TGFBI	transforming growth factor beta induced [Source:NCBI gene;Acc:395897]	4.83729194758756e-176	0,423870259	0.434	0.172	1,18E-171	5	CT
91	ENSGALG00000003923	COL6A3	collagen type VI alpha 3 chain [Source:NCBI gene;Acc:396548]	1.05950127319212e-76	0,410589762	0.391	0.222	2,58E-72	5	CT
381	ENSGALG00000016511	ADGRG2	adhesion G protein-coupled receptor G2 [Source:NCBI gene;Acc:418611]	0	0,388183637	0.338	0.038	0	5	CT
187	ENSGALG00000008367	MDK	midkine (neurite growth-promoting factor 2) [Source:NCBI gene;Acc:423196]	5.53591025371564e-51	0,371812542	0.573	0.467	1,35E-46	5	CT
468	ENSGALG00000028520	CST3	cystatin C [Source:NCBI gene;Acc:396497]	1.75090447104106e-44	0,355911342	0.425	0.302	4,26E-40	5	CT
611	ENSGALG00000038775	SPON1	spondin 1 [Source:NCBI gene;Acc:395657]	9.5485577489386e-152	0,287379617	0.2	0.048	2,33E-147	5	CT
372	ENSGALG00000016289	DST	dystonin [Source:NCBI gene;Acc:421884]	1.39980335936714e-88	0,2852944	0.389	0.205	3,41E-84	5	CT
595	ENSGALG00000037675	COL14A1	collagen type XIV alpha 1 chain [Source:NCBI gene;Acc:396276]	3.40862557264147e-78	0,284584244	0.399	0.211	8,30E-74	5	CT
141	ENSGALG00000006346	CXCL14	C-X-C motif chemokine ligand 14 [Source:NCBI gene;Acc:395451]	3.52478479670265e-47	0,276760785	0.227	0.113	8,58E-43	5	CT
336	ENSGALG00000014970	FSTL1	follistatin like 1 [Source:NCBI gene;Acc:395349]	1.60196706228546e-69	0,256272267	0.629	0.471	3,90E-65	5	CT
110	ENSGALG00000005135	SERPINE2	serpin family E member 2 [Source:NCBI gene;Acc:424805]	1.6801450015142e-75	0,256052059	0.357	0.187	4,09E-71	5	CT
649	ENSGALG00000041555	COL1A1	collagen type I alpha 1 chain [Source:NCBI gene;Acc:395532]	0	1,115559886	0.993	0.782	0	6	CT
641	ENSGALG00000040493	PTN	pleiotrophin [Source:NCBI gene;Acc:418125]	0	1,083167347	0.668	0.192	0	6	CT
367	ENSGALG00000015908	COL12A1	collagen type XII alpha 1 chain [Source:NCBI gene;Acc:395875]	0	0,992898049	0.806	0.264	0	6	CT
626	ENSGALG00000039985	CDH11	cadherin 11 [Source:NCBI gene;Acc:415797]	6.85129286416564e-307	0,738010494	0.852	0.465	1,67E-302	6	CT
82	ENSGALG00000003670	MAFB	MAF bZIP transcription factor B [Source:NCBI gene;Acc:419173]	0	0,644556276	0.549	0.134	0	6	CT
280	ENSGALG00000011708	MYLK	myosin light chain kinase [Source:NCBI gene;Acc:396445]	0	0,619261288	0.429	0.104	0	6	CT
61	ENSGALG00000003324	PRRX1	paired related homeobox 1 [Source:NCBI gene;Acc:373941]	1.00160516986643e-130	0,59028646	0.486	0.24	2,44E-126	6	CT
327	ENSGALG00000014804	THBS4	thrombospondin 4 [Source:NCBI gene;Acc:396306]	0	0,532112466	0.417	0.099	0	6	CT

55	ENSGALG00000003193	CRABP-I	cellular retinoic acid binding protein 1 [Source:NCBI gene;Acc:374211]	7.04225104887303e-87	0,502039454	0.498	0.279	1,72E-82	6	CT
119	ENSGALG00000005551	DKK3	dickkopf WNT signaling pathway inhibitor 3 [Source:NCBI gene;Acc:396023]	3.92836523093573e-271	0,49749743	0.485	0.146	9,57E-267	6	CT
596	ENSGALG00000037675	COL14A1	collagen type XIV alpha 1 chain [Source:NCBI gene;Acc:396276]	1.81243022846308e-137	0,4634011	0.476	0.208	4,41E-133	6	CT
142	ENSGALG00000006346	CXCL14	C-X-C motif chemokine ligand 14 [Source:NCBI gene;Acc:395451]	8.63384987882322e-82	0,45842141	0.273	0.112	2,10E-77	6	CT
225	ENSGALG00000009641	COL1A2	collagen type I alpha 2 chain [Source:NCBI gene;Acc:396243]	1.34672996077034e-152	0,444190674	0.556	0.269	3,28E-148	6	CT
350	ENSGALG00000015253	COL8A1	collagen type VIII alpha 1 chain [Source:NCBI gene;Acc:418378]	0	0,438151796	0.23	0.007	0	6	CT
265	ENSGALG00000011274	DCN	decorin [Source:NCBI gene;Acc:417892]	1.06510657639995e-131	0,437189439	0.906	0.689	2,59E-127	6	CT
39	ENSGALG00000002546	COL5A1	collagen type V alpha 1 chain [Source:NCBI gene;Acc:395568]	4.17817693859761e-141	0,420286895	0.947	0.702	1,02E-136	6	CT
75	ENSGALG00000003580	MMP2	matrix metallopeptidase 2 [Source:NCBI gene;Acc:386583]	5.09593152675058e-134	0,419630143	0.622	0.336	1,24E-129	6	CT
348	ENSGALG00000015193	CCDC80	coiled-coil domain containing 80 [Source:NCBI gene;Acc:395074]	1.55986971594246e-142	0,382212945	0.657	0.349	3,80E-138	6	CT
191	ENSGALG00000008437	NET1	neuroepithelial cell transforming 1 [Source:NCBI gene;Acc:416693]	5.39239965495243e-173	0,381944416	0.378	0.127	1,31E-168	6	CT
509	ENSGALG00000031424	SDC2	syndecan 2 [Source:NCBI gene;Acc:374102]	7.97984911476701e-109	0,378376823	0.596	0.334	1,94E-104	6	CT
482	ENSGALG00000029429	TGFBI	transforming growth factor beta induced [Source:NCBI gene;Acc:395897]	1.45386225636086e-134	0,375826411	0.436	0.176	3,54E-130	6	CT
414	ENSGALG00000023904	FIBIN	fin bud initiation factor homolog (zebrafish) [Source:NCBI gene;Acc:426933]	3.24434902151196e-164	0,374419183	0.459	0.174	7,90E-160	6	CT
95	ENSGALG00000004184	SPARC	secreted protein acidic and cysteine rich [Source:NCBI gene;Acc:386571]	2.89235989762537e-94	0,367619011	0.757	0.552	7,04E-90	6	CT
333	ENSGALG00000014970	FSTL1	follistatin like 1 [Source:NCBI gene;Acc:395349]	2.18427794628431e-103	0,363364452	0.689	0.469	5,32E-99	6	CT
210	ENSGALG00000009241	SFRP2	secreted frizzled related protein 2 [Source:NCBI gene;Acc:395546]	2.24170097267311e-78	0,35838126	0.461	0.249	5,46E-74	6	CT
511	ENSGALG00000031425	PMP22	peripheral myelin protein 22 [Source:NCBI gene;Acc:417327]	9.13022781701343e-130	0,347557034	0.432	0.184	2,22E-125	6	CT
535	ENSGALG00000033351	CTHRC1	collagen triple helix repeat containing 1 [Source:HGNC Symbol;Acc:HGNC:18831]	5.41119708053452e-215	0,33412472	0.401	0.117	1,32E-210	6	CT
384	ENSGALG00000016635	MXRA5	matrix remodeling associated 5 [Source:NCBI gene;Acc:418657]	1.20385558981722e-147	0,332076771	0.431	0.166	2,93E-143	6	CT
111	ENSGALG00000005135	SERPINE2	serpin family E member 2 [Source:NCBI gene;Acc:424805]	9.51487949784657e-147	0,329142496	0.454	0.183	2,32E-142	6	CT
456	ENSGALG00000027891	NREP	neuronal regeneration related protein [Source:NCBI gene;Acc:396353]	1.63738826956588e-56	0,291780547	0.707	0.546	3,99E-52	6	CT
631	ENSGALG00000040010	ENSGALG00000040010		1.74263216228898e-218	0,291753772	0.249	0.047	4,24E-214	6	CT
713	ENSGALG00000049529	ENSGALG00000049529		2.59946037805676e-177	0,290712042	0.251	0.057	6,33E-173	6	CT
52	ENSGALG00000003015	SERPINF1	serpin family F member 1 [Source:NCBI gene;Acc:417561]	1.58452460370653e-99	0,290645063	0.412	0.192	3,86E-95	6	CT
658	ENSGALG00000041826	S100A6	S100 calcium binding protein A6 [Source:NCBI gene;Acc:373951]	6.09045538693086e-69	0,288274637	0.346	0.174	1,48E-64	6	CT
216	ENSGALG00000009495	FGFR2	fibroblast growth factor receptor 2 [Source:NCBI gene;Acc:396259]	3.46443008818394e-209	0,284973466	0.271	0.058	8,44E-205	6	CT
153	ENSGALG00000006821	TNMD	tenomodulin [Source:NCBI gene;Acc:404530]	7.37329822099113e-110	0,284012123	0.342	0.132	1,80E-105	6	CT
65	ENSGALG00000003473	SFRP1	secreted frizzled related protein 1 [Source:NCBI gene;Acc:395237]	2.84247541179317e-99	0,263025793	0.356	0.151	6,92E-95	6	CT
539	ENSGALG00000033471	CALD1	caldesmon 1 [Source:NCBI gene;Acc:373965]	8.62518440317963e-144	0,257373414	0.331	0.106	2,10E-139	6	CT
695	ENSGALG00000043690	ECRG4	chromosome 1 open reading frame, human C2orf40 [Source:NCBI gene;Acc:771055]	2.33482555953037e-62	0,255173681	0.558	0.365	5,69E-58	6	CT
253	ENSGALG00000011200	THBS2	thrombospondin 2 [Source:NCBI gene;Acc:414837]	4.68817997054749e-245	0,255165346	0.25	0.043	1,14E-240	6	CT
291	ENSGALG00000012420	CG-1B	galectin 1 [Source:NCBI gene;Acc:396491]	3.13097693828753e-147	0,250385152	0.21	0.047	7,63E-143	6	CT
135	ENSGALG00000006252	LMO4	LIM domain only 4 [Source:NCBI gene;Acc:373901]	4.38306644887356e-48	0,250240939	0.409	0.258	1,07E-43	6	CT
651	ENSGALG00000041555	COL1A1	collagen type I alpha 1 chain [Source:NCBI gene;Acc:395532]	0	1,568836071	1	0.782	0	7	CT
366	ENSGALG00000015908	COL12A1	collagen type XII alpha 1 chain [Source:NCBI gene;Acc:395875]	0	1,064709412	0.903	0.259	0	7	CT
597	ENSGALG00000037675	COL14A1	collagen type XIV alpha 1 chain [Source:NCBI gene;Acc:396276]	0	0,937759334	0.69	0.196	0	7	CT
395	ENSGALG00000017046	POSTN	periostin [Source:NCBI gene;Acc:395429]	1.05556786496595e-224	0,932647746	0.688	0.315	2,57E-220	7	CT
642	ENSGALG00000040493	PTN	pleiotrophin [Source:NCBI gene;Acc:418125]	0	0,865446114	0.611	0.197	0	7	CT
263	ENSGALG00000011274	DCN	decorin [Source:NCBI gene;Acc:417892]	5.37673930293488e-256	0,672471353	0.958	0.686	1,31E-251	7	CT
224	ENSGALG00000009641	COL1A2	collagen type I alpha 2 chain [Source:NCBI gene;Acc:396243]	0	0,666170105	0.762	0.256	0	7	CT
94	ENSGALG00000004184	SPARC	secreted protein acidic and cysteine rich [Source:NCBI gene;Acc:386571]	7.18101619945927e-258	0,663895881	0.863	0.546	1,75E-253	7	CT
484	ENSGALG00000029429	TGFBI	transforming growth factor beta induced [Source:NCBI gene;Acc:395897]	0	0,647509337	0.667	0.162	0	7	CT
565	ENSGALG00000035584	COL3A1	collagen type III alpha 1 chain [Source:NCBI gene;Acc:396340]	9.79064044250324e-268	0,64554471	0.948	0.501	2,38E-263	7	CT
152	ENSGALG00000006821	TNMD	tenomodulin [Source:NCBI gene;Acc:404530]	0	0,616529928	0.533	0.12	0	7	CT
416	ENSGALG00000023904	FIBIN	fin bud initiation factor homolog (zebrafish) [Source:NCBI gene;Acc:426933]	0	0,611817412	0.656	0.162	0	7	CT
534	ENSGALG00000033351	CTHRC1	collagen triple helix repeat containing 1 [Source:HGNC Symbol;Acc:HGNC:18831]	0	0,49707163	0.566	0.107	0	7	CT
364	ENSGALG00000015720	CHODL	chondrolectin [Source:NCBI gene;Acc:418474]	0	0,495901152	0.555	0.111	0	7	CT
79	ENSGALG00000003655	GAS2	growth arrest specific 2 [Source:NCBI gene;Acc:422970]	0	0,485171094	0.563	0.096	0	7	CT
192	ENSGALG00000008444	TUBAL3	tubulin alpha like 3 [Source:NCBI gene;Acc:416694]	0	0,473546876	0.515	0.128	0	7	CT
37	ENSGALG00000002546	COL5A1	collagen type V alpha 1 chain [Source:NCBI gene;Acc:395568]	5.1450467805827e-173	0,466344481	0.971	0.701	1,25E-168	7	CT
689	ENSGALG00000043433	SCX	scleraxis bHLH transcription factor [Source:NCBI gene;Acc:374101]	0	0,463568429	0.429	0.017	0	7	CT
71	ENSGALG00000003578	FN1	fibronectin 1 [Source:NCBI gene;Acc:396133]	1.08236336683323e-143	0,456460544	0.811	0.507	2,64E-139	7	CT
256	ENSGALG00000011270	KERA	keratocan [Source:NCBI gene;Acc:373995]	1.82344982280844e-293	0,451797938	0.512	0.138	4,44E-289	7	CT
254	ENSGALG00000011214	SERPINH1	serpin family H member 1 [Source:NCBI gene;Acc:396228]	4.73869412661602e-211	0,446540121	0.736	0.366	1,15E-206	7	CT

118	ENSGALG00000005551	DKK3	dickkopf WNT signaling pathway inhibitor 3 [Source:NCBI gene;Acc:396023]	0	0,439779137	0.537	0.144	0	7	CT
180	ENSGALG00000008007	ITGA11	integrin subunit alpha 11 [Source:NCBI gene;Acc:415560]	0	0,434448448	0.355	0.026	0	7	CT
659	ENSGALG00000041918	CPA6	carboxypeptidase A6 [Source:NCBI gene;Acc:420171]	0	0,398651636	0.395	0.029	0	7	CT
676	ENSGALG00000043035	SHISA2	shisa family member 2 [Source:NCBI gene;Acc:395162]	0	0,38419507	0.473	0.109	0	7	CT
694	ENSGALG00000043690	ECRG4	chromosome 1 open reading frame, human C2orf40 [Source:NCBI gene;Acc:771055]	6.52378250605238e-131	0,381153995	0.653	0.359	1,59E-126	7	CT
112	ENSGALG00000005180	ENSGALG00000005180		0	0,312003857	0.347	0.03	0	7	CT
57	ENSGALG00000003193	CRABP-I	cellular retinoic acid binding protein 1 [Source:NCBI gene;Acc:374211]	5.12632085623438e-139	0,307066777	0.602	0.273	1,25E-134	7	CT
463	ENSGALG00000028354	METRNL	meteorin, glial cell differentiation regulator-like [Source:NCBI gene;Acc:417332]	7.84240014585269e-168	0,306627045	0.502	0.191	1,91E-163	7	CT
248	ENSGALG00000010794	MEOX2	mesenchyme homeobox 2 [Source:NCBI gene;Acc:374137]	9.49247787108256e-227	0,301440075	0.421	0.114	2,31E-222	7	CT
656	ENSGALG00000041750	ADAMTS9	ADAM metallopeptidase with thrombospondin type 1 motif 9 [Source:NCBI gene;Acc:416080]	1.95593228300794e-179	0,301038051	0.373	0.109	4,76E-175	7	CT
13	ENSGALG00000000713	ZFHX3	zinc finger homeobox 3 [Source:NCBI gene;Acc:395682]	2.44389859080122e-172	0,297261686	0.456	0.159	5,95E-168	7	CT
604	ENSGALG00000038433	SPON2	spondin 2 [Source:NCBI gene;Acc:422905]	0	0,274762961	0.189	0.017	0	7	CT
292	ENSGALG00000012420	CG-1B	galectin 1 [Source:NCBI gene;Acc:396491]	7.5900339972769e-109	0,267980698	0.191	0.049	1,85E-104	7	CT
630	ENSGALG00000039990	TNC	tenascin C [Source:NCBI gene;Acc:396440]	1.10307912100109e-294	0,267539973	0.303	0.05	2,69E-290	7	CT
274	ENSGALG00000011663	FNDC1	fibronectin type III domain containing 1 [Source:NCBI gene;Acc:421589]	0	0,261378256	0.282	0.026	0	7	CT
629	ENSGALG00000039985	CDH11	cadherin 11 [Source:NCBI gene;Acc:415797]	1.86328903368261e-90	0,256400216	0.785	0.47	4,54E-86	7	CT
53	ENSGALG00000003015	SERPINF1	serpin family F member 1 [Source:NCBI gene;Acc:417561]	2.54653045861519e-122	0,252221666	0.457	0.19	6,20E-118	7	CT
354	ENSGALG00000015419	PENK	proenkephalin [Source:NCBI gene;Acc:421131]	0	1,765617978	0.982	0.189	0	8	M
297	ENSGALG00000012712	RBM24	RNA binding motif protein 24 [Source:NCBI gene;Acc:420846]	0	1,218353057	0.927	0.109	0	8	M
247	ENSGALG00000010745	HMGB2	high mobility group box 2 [Source:NCBI gene;Acc:396482]	0	0,891038153	0.876	0.328	0	8	M
512	ENSGALG00000031593	TMSB15B	thymosin beta 15B [Source:NCBI gene;Acc:100502566]	0	0,773821509	0.931	0.533	0	8	M
132	ENSGALG00000006216	MYOD1	myogenic differentiation 1 [Source:NCBI gene;Acc:374048]	0	0,728877813	0.74	0.095	0	8	M
576	ENSGALG00000036441	WIPF1	WAS/WASL interacting protein family member 1 [Source:NCBI gene;Acc:424143]	0	0,63087663	0.731	0.105	0	8	M
109	ENSGALG00000004956	GPI	glucose-6-phosphate isomerase [Source:NCBI gene;Acc:415783]	0	0,622518767	0.764	0.183	0	8	M
722	ENSGALG00000052192	TUBA1B	tubulin alpha-1A chain-like [Source:NCBI gene;Acc:100859737]	2.44313048462843e-244	0,565121367	0.954	0.678	5,95E-240	8	M
590	ENSGALG00000037167	HIST1H103	histone cluster 1, H1.03 (similar to human histone cluster 1, class H1 genes) [Source:NCBI gene;Acc	2.12766284347786e-159	0,495136766	0.44	0.159	5,18E-155	8	M
471	ENSGALG00000028543	FGFR4	fibroblast growth factor receptor 4 [Source:NCBI gene;Acc:395603]	0	0,486055587	0.629	0.131	0	8	M
544	ENSGALG00000033677	AHCY	adenosylhomocysteinase [Source:NCBI gene;Acc:419146]	5.88368982967659e-285	0,473067684	0.745	0.289	1,43E-280	8	M
115	ENSGALG00000005263	SOX8	SRY-box 8 [Source:NCBI gene;Acc:395483]	0	0,457480503	0.583	0.11	0	8	M
181	ENSGALG00000008094	HSPD1	heat shock protein family D (Hsp60) member 1 [Source:NCBI gene;Acc:424059]	6.36552301249494e-214	0,447670977	0.783	0.41	1,55E-209	8	M
89	ENSGALG00000003922	TOP2A	topoisomerase (DNA) II alpha [Source:NCBI gene;Acc:395570]	4.08082808762874e-247	0,441130708	0.676	0.245	9,94E-243	8	M
427	ENSGALG00000025996	MKI67	marker of proliferation Ki-67 [Source:NCBI gene;Acc:423963]	6.33678533744304e-292	0,428622821	0.55	0.154	1,54E-287	8	M
531	ENSGALG00000033212	HSP90AA1	heat shock protein 90 alpha family class A member 1 [Source:NCBI gene;Acc:423463]	6.70547004641806e-138	0,42772376	0.925	0.726	1,63E-133	8	M
498	ENSGALG00000030587	SPTBB4L	spectrin beta chain, brain 4-like [Source:NCBI gene;Acc:421441]	0	0,420653508	0.486	0.079	0	8	M
271	ENSGALG00000011511	CKB	creatine kinase B [Source:NCBI gene;Acc:396248]	0	0,419620863	0.5	0.112	0	8	M
505	ENSGALG00000031275	TUBB6	tubulin beta 6 class V [Source:NCBI gene;Acc:421037]	0	0,412513929	0.522	0.096	0	8	M
177	ENSGALG00000007839	NCAM1	neural cell adhesion molecule 1 [Source:NCBI gene;Acc:428253]	1.90310477759796e-250	0,400843304	0.706	0.265	4,64E-246	8	M
226	ENSGALG00000009690	CENPF	centromere protein F [Source:NCBI gene;Acc:395357]	1.99094061066576e-192	0,392959535	0.494	0.16	4,85E-188	8	M
54	ENSGALG00000003085	CDK1	cyclin dependent kinase 1 [Source:NCBI gene;Acc:396252]	7.03091571771552e-266	0,391438087	0.563	0.17	1,71E-261	8	M
76	ENSGALG00000003584	KPNA2	karyopherin subunit alpha 2 [Source:NCBI gene;Acc:417420]	5.76467597949103e-143	0,388789452	0.429	0.151	1,40E-138	8	M
674	ENSGALG00000042875	HMGB1	high mobility group box 1 [Source:NCBI gene;Acc:395724]	3.55716882442795e-159	0,381978586	0.966	0.785	8,66E-155	8	M
316	ENSGALG00000014023	H2AFZ	H2A histone family, member Z [Source:NCBI gene;Acc:426361]	4.41826074190174e-156	0,364530347	0.767	0.417	1,08E-151	8	M
520	ENSGALG00000032398	ASPM	abnormal spindle microtubule assembly [Source:NCBI gene;Acc:424354]	7.435380436797e-194	0,364230629	0.506	0.168	1,81E-189	8	M
359	ENSGALG00000015691	SMC2	structural maintenance of chromosomes 2 [Source:NCBI gene;Acc:396156]	1.10214078401432e-162	0,363363169	0.692	0.306	2,68E-158	8	M
12	ENSGALG00000000708	NUCKS1	nuclear casein kinase and cyclin dependent kinase substrate 1 [Source:NCBI gene;Acc:404775]	3.53298346360152e-117	0,333618842	0.823	0.538	8,60E-113	8	M
125	ENSGALG00000005930	PLS3	plastin 3 [Source:NCBI gene;Acc:422222]	2.40068167250546e-196	0,32776663	0.537	0.186	5,85E-192	8	M
10	ENSGALG00000000470	LMNA	lamin A/C [Source:NCBI gene;Acc:396222]	3.68882692423059e-119	0,322169725	0.73	0.413	8,98E-115	8	M
550	ENSGALG00000034271	UHRF1	ubiquitin like with PHD and ring finger domains 1 [Source:NCBI gene;Acc:420154]	2.8737335234799e-187	0,32054849	0.564	0.212	7,00E-183	8	M
24	ENSGALG00000001472	JAM3	junctional adhesion molecule 3 [Source:NCBI gene;Acc:419736]	4.62860994481502e-171	0,315344119	0.613	0.246	1,13E-166	8	M
738	ENSGALG00000054420	TUBB2A	tubulin, beta 2A class IIa [Source:NCBI gene;Acc:768337]	3.61014463792905e-194	0,313271573	0.546	0.194	8,79E-190	8	M
137	ENSGALG00000006267	TPX2	TPX2, microtubule nucleation factor [Source:NCBI gene;Acc:395081]	6.11116061228752e-182	0,301739676	0.472	0.153	1,49E-177	8	M
436	ENSGALG00000026380	UBE2C	ubiquitin conjugating enzyme E2 U (putative) [Source:NCBI gene;Acc:100859413]	2.90618047004894e-222	0,299119801	0.422	0.112	7,08E-218	8	M
209	ENSGALG00000009107	NRXN1	neurexin 1 [Source:NCBI gene;Acc:395398]	4.73146895887956e-211	0,293297351	0.403	0.106	1,15E-206	8	M
18	ENSGALG00000001171	SEMA6B	semaphorin 6B [Source:NCBI gene;Acc:428330]	0	0,286318903	0.367	0.039	0	8	M
430	ENSGALG00000026081	RBM38	RNA binding motif protein 38 [Source:NCBI gene;Acc:768866]	0	0,273191172	0.357	0.046	0	8	M

173	ENSGALG00000007537	INCENP	inner centromere protein [Source:NCBI gene;Acc:396270]	4.33649071928397e-192	0,270929316	0.429	0.127	1,06E-187	8	M
731	ENSGALG00000053871	ENSGALG00000053871		3.46089274582033e-137	0,260033742	0.559	0.237	8,43E-133	8	M
445	ENSGALG00000026940	TTYH2	tweety family member 1 [Source:NCBI gene;Acc:100858879]	1.16302585103986e-204	0,257568678	0.417	0.115	2,83E-200	8	M
96	ENSGALG00000004195	KIF4B	kinesin family member 4B [Source:NCBI gene;Acc:395823]	1.72330419390672e-160	0,253878159	0.438	0.145	4,20E-156	8	M
227	ENSGALG00000009844	ACTC1	actin alpha cardiac muscle 1 [Source:NCBI gene;Acc:423298]	0	2,120333071	0.909	0.093	0	9	M
514	ENSGALG00000031593	TMSB15B	thymosin beta 15B [Source:NCBI gene;Acc:100502566]	0	1,765059618	0.999	0.538	0	9	M
298	ENSGALG00000012712	RBM24	RNA binding motif protein 24 [Source:NCBI gene;Acc:420846]	0	1,651582584	0.982	0.124	0	9	M
355	ENSGALG00000015419	PENK	proenkephalin [Source:NCBI gene;Acc:421131]	0	1,611937561	0.981	0.205	0	9	M
497	ENSGALG00000030587	SPTBB4L	spectrin beta chain, brain 4-like [Source:NCBI gene;Acc:421441]	0	1,329555215	0.921	0.071	0	9	M
126	ENSGALG00000005930	PLS3	plastin 3 [Source:NCBI gene;Acc:422222]	0	1,13950839	0.905	0.18	0	9	M
23	ENSGALG00000001472	JAM3	junctional adhesion molecule 3 [Source:NCBI gene;Acc:419736]	0	1,09340653	0.909	0.243	0	9	M
543	ENSGALG00000033677	AHCY	adenosylhomocysteinase [Source:NCBI gene;Acc:419146]	0	1,043048282	0.93	0.291	0	9	M
269	ENSGALG00000011511	CKB	creatine kinase B [Source:NCBI gene;Acc:396248]	0	0,933937186	0.759	0.111	0	9	M
155	ENSGALG00000006835	TNNC2	troponin C2, fast skeletal type [Source:NCBI gene;Acc:396434]	0	0,777415325	0.523	0.056	0	9	M
575	ENSGALG00000036441	WIPF1	WAS/WASL interacting protein family member 1 [Source:NCBI gene;Acc:424143]	0	0,756398583	0.741	0.118	0	9	M
319	ENSGALG00000014442	GAPDH	glyceraldehyde-3-phosphate dehydrogenase [Source:NCBI gene;Acc:374193]	5.60157678726398e-258	0,735879671	0.995	0.939	1,36E-253	9	M
108	ENSGALG00000004956	GPI	glucose-6-phosphate isomerase [Source:NCBI gene;Acc:415783]	0	0,687929529	0.742	0.195	0	9	M
4	ENSGALG00000000172	MYOG	myogenin [Source:NCBI gene;Acc:374004]	0	0,67278068	0.61	0.022	0	9	M
131	ENSGALG00000006216	MYOD1	myogenic differentiation 1 [Source:NCBI gene;Acc:374048]	0	0,659127474	0.688	0.11	0	9	M
410	ENSGALG00000021520	TMEM8C	transmembrane protein 8C [Source:NCBI gene;Acc:777472]	0	0,539286503	0.496	0.005	0	9	M
102	ENSGALG00000004518	BHMT2	betaine--homocysteine S-methyltransferase 2 [Source:NCBI gene;Acc:416371]	0	0,536979122	0.544	0.037	0	9	M
235	ENSGALG00000010027	CFL2	cofilin 2 [Source:NCBI gene;Acc:423320]	0	0,535378235	0.58	0.126	0	9	M
718	ENSGALG00000051776	ENSGALG00000051776		0	0,528201296	0.437	0.021	0	9	M
412	ENSGALG00000022980	CDKN1B	cyclin dependent kinase inhibitor 1B [Source:NCBI gene;Acc:374106]	0	0,527929667	0.596	0.123	0	9	M
635	ENSGALG00000040316	GPC1	glypican 1 [Source:NCBI gene;Acc:424770]	1.11002820151008e-201	0,516089343	0.724	0.281	2,70E-197	9	M
207	ENSGALG00000009081	ZNF106	zinc finger protein 106 [Source:NCBI gene;Acc:423234]	0	0,515788156	0.508	0.046	0	9	M
730	ENSGALG00000053371	ENSGALG00000053371		2.49802182739243e-250	0,504022561	0.635	0.181	6,08E-246	9	M
19	ENSGALG00000001171	SEMA6B	semaphorin 6B [Source:NCBI gene;Acc:428330]	0	0,487380489	0.518	0.04	0	9	M
726	ENSGALG00000053246	OCM2	oncomodulin 2 [Source:NCBI gene;Acc:396531]	0	0,476154735	0.419	0.063	0	9	M
179	ENSGALG00000007945	CRYAB	crystallin alpha B [Source:NCBI gene;Acc:396089]	0	0,466060169	0.459	0.022	0	9	M
467	ENSGALG00000028451	MT4	metallothionein 4 [Source:NCBI gene;Acc:396212]	0	0,449585383	0.345	0.035	0	9	M
506	ENSGALG00000031275	TUBB6	tubulin beta 6 class V [Source:NCBI gene;Acc:421037]	9.01068218143263e-297	0,436755821	0.521	0.105	2,19E-292	9	M
428	ENSGALG00000026081	RBM38	RNA binding motif protein 38 [Source:NCBI gene;Acc:768866]	0	0,429780674	0.457	0.048	0	9	M
158	ENSGALG00000006904	RNH1	ribonuclease/angiogenin inhibitor 1 [Source:NCBI gene;Acc:423111]	5.77237311478121e-163	0,426194723	0.499	0.156	1,41E-158	9	M
662	ENSGALG00000042020	CG-16	galectin CG-16 [Source:NCBI gene;Acc:404269]	7.41805715528656e-240	0,419409806	0.4	0.076	1,81E-235	9	M
707	ENSGALG00000047289	TNFAIP8L3	TNF alpha induced protein 8 like 3 [Source:NCBI gene;Acc:427499]	0	0,417317197	0.475	0.077	0	9	M
599	ENSGALG00000037784	DAG1	dystroglycan 1 [Source:NCBI gene;Acc:100049058]	2.45877409294238e-202	0,417237288	0.516	0.141	5,99E-198	9	M
295	ENSGALG00000012691	ALDH1L2	aldehyde dehydrogenase 1 family member L2 [Source:NCBI gene;Acc:418078]	9.22892516295599e-241	0,397823512	0.407	0.078	2,25E-236	9	M
190	ENSGALG00000008425	MAP1A	microtubule associated protein 1A [Source:NCBI gene;Acc:415588]	0	0,389531415	0.475	0.079	0	9	M
736	ENSGALG00000054420	TUBB2A	tubulin, beta 2A class IIa [Source:NCBI gene;Acc:768337]	5.12218844458867e-128	0,370994359	0.529	0.201	1,25E-123	9	M
569	ENSGALG00000035994	ENSGALG0000003599	probable acyl coa dehydrogenase 6-like [Source:NCBI gene;Acc:420562]	0	0,36691795	0.396	0.052	0	9	M
580	ENSGALG00000036785	TRIM8	tripartite motif containing 8 [Source:NCBI gene;Acc:423864]	1.85133308245079e-188	0,351924323	0.466	0.12	4,51E-184	9	M
711	ENSGALG00000048053	ENSGALG00000048053		0	0,342783259	0.322	0.003	0	9	M
300	ENSGALG00000012715	CAP2	cyclase associated actin cytoskeleton regulatory protein 2 [Source:NCBI gene;Acc:420847]	0	0,326716635	0.357	0.044	0	9	M
50	ENSGALG00000002907	MYL1	myosin, light chain 1, alkali; skeletal, fast [Source:NCBI gene;Acc:396470]	1.09159542114003e-287	0,322345288	0.262	0.026	2,66E-283	9	M
178	ENSGALG00000007839	NCAM1	neural cell adhesion molecule 1 [Source:NCBI gene;Acc:428253]	1.39941313158042e-90	0,322039439	0.59	0.278	3,41E-86	9	M
98	ENSGALG00000004273	RALGPS2	Ral GEF with PH domain and SH3 binding motif 2 [Source:NCBI gene;Acc:424429]	5.26399217560245e-217	0,316561881	0.401	0.08	1,28E-212	9	M
279	ENSGALG00000011695	HIPK3	homeodomain interacting protein kinase 3 [Source:NCBI gene;Acc:421597]	7.07176344568278e-180	0,316323093	0.412	0.099	1,72E-175	9	M
238	ENSGALG00000010186	CAPN11	calpain 11 [Source:NCBI gene;Acc:396240]	1.93438433844861e-166	0,312365516	0.396	0.097	4,71E-162	9	M
398	ENSGALG00000017179	PDGFD	platelet derived growth factor D [Source:NCBI gene;Acc:418978]	4.4314052595012e-93	0,311971213	0.545	0.236	1,08E-88	9	M
290	ENSGALG00000012370	GINM1	glycoprotein integral membrane 1 [Source:NCBI gene;Acc:395778]	8.15108472671131e-300	0,308100402	0.343	0.044	1,99E-295	9	M
426	ENSGALG00000025977	GADD45A	growth arrest and DNA damage inducible alpha [Source:NCBI gene;Acc:693255]	3.26765444266524e-192	0,299505785	0.381	0.081	7,96E-188	9	M
273	ENSGALG00000011592	MRAS	muscle RAS oncogene homolog [Source:NCBI gene;Acc:395149]	7.16490454185273e-190	0,299368621	0.386	0.082	1,75E-185	9	M
577	ENSGALG00000036471	RFTN2	raftlin family member 2 [Source:NCBI gene;Acc:424062]	1.69990067090055e-176	0,290409133	0.358	0.076	4,14E-172	9	M
533	ENSGALG00000033226	FAM107B	family with sequence similarity 107 member B [Source:NCBI gene;Acc:426376]	1.50139214729581e-178	0,287235533	0.371	0.08	3,66E-174	9	M

499	ENSGALG00000030878	RPLP1	ribosomal protein lateral stalk subunit P1 [Source:NCBI gene;Acc:396262]	2.50856988735207e-105	0,285133882	0.999	0.998	6,11E-101	9	M
677	ENSGALG00000043035	SHISA2	shisa family member 2 [Source:NCBI gene;Acc:395162]	1.52028447822439e-125	0,283118227	0.409	0.12	3,70E-121	9	M
114	ENSGALG00000005263	SOX8	SRY-box 8 [Source:NCBI gene;Acc:395483]	7.15973114691775e-119	0,276330365	0.411	0.126	1,74E-114	9	M
310	ENSGALG00000013239	ENSGALG00000013239		1.53482070348588e-97	0,273403496	0.348	0.111	3,74E-93	9	M
342	ENSGALG00000015132	CDH2	cadherin 2 [Source:NCBI gene;Acc:414745]	9.17156366878651e-61	0,270089218	0.406	0.182	2,23E-56	9	M
494	ENSGALG00000030040	CDH15	acyl-CoA synthetase family member 3 [Source:NCBI gene;Acc:427561]	1.81426682997685e-217	0,267682242	0.322	0.053	4,42E-213	9	M
698	ENSGALG00000045196	LAPTM4B	lysosomal protein transmembrane 4 beta [Source:NCBI gene;Acc:770893]	1.802313694375e-167	0,261220886	0.34	0.071	4,39E-163	9	M
338	ENSGALG00000014994	CRHBP	corticotropin releasing hormone binding protein [Source:NCBI gene;Acc:427214]	3.01736215331845e-209	0,827849845	0.427	0.056	7,35E-205	11	CT
72	ENSGALG00000003578	FN1	fibronectin 1 [Source:NCBI gene;Acc:396133]	1.4739226339029e-110	0,818905671	0.902	0.519	3,59E-106	11	CT
161	ENSGALG00000007000	NR2F2	nuclear receptor subfamily 2 group F member 2 [Source:NCBI gene;Acc:386585]	1.02678179430622e-222	0,794885175	0.719	0.16	2,50E-218	11	CT
469	ENSGALG00000028520	CST3	cystatin C [Source:NCBI gene;Acc:396497]	2.08459460529259e-60	0,649126882	0.621	0.306	5,08E-56	11	CT
648	ENSGALG00000041346	CXCL12	C-X-C motif chemokine ligand 12 [Source:NCBI gene;Acc:395180]	3.85197572257328e-173	0,647208847	0.538	0.105	9,38E-169	11	CT
259	ENSGALG00000011271	LUM	lumican [Source:NCBI gene;Acc:417891]	1.99709231518343e-114	0,640551475	0.653	0.212	4,86E-110	11	CT
396	ENSGALG00000017046	POSTN	periostin [Source:NCBI gene;Acc:395429]	1.99025610228539e-99	0,622574882	0.827	0.328	4,85E-95	11	CT
277	ENSGALG00000011687	AHNAK2	AHNAK nucleoprotein 2 [Source:NCBI gene;Acc:100859120]	4.15753691661495e-76	0,607316165	0.513	0.181	1,01E-71	11	CT
335	ENSGALG00000014970	FSTL1	follistatin like 1 [Source:NCBI gene;Acc:395349]	6.92316758295217e-69	0,60500884	0.786	0.477	1,69E-64	11	CT
231	ENSGALG00000010013	EDNRA	endothelin receptor type A [Source:NCBI gene;Acc:373908]	2.9826597130607e-184	0,538919583	0.53	0.096	7,26E-180	11	CT
402	ENSGALG00000017644	COTL1	coactosin like F-actin binding protein 1 [Source:NCBI gene;Acc:768420]	6.07895530503165e-64	0,53032971	0.726	0.377	1,48E-59	11	CT
421	ENSGALG00000025822	CYP1B1	cytochrome P450 family 1 subfamily B member 1 [Source:NCBI gene;Acc:421466]	2.58116454111695e-162	0,523208575	0.384	0.057	6,29E-158	11	CT
92	ENSGALG00000003923	COL6A3	collagen type VI alpha 3 chain [Source:NCBI gene;Acc:396548]	4.16016986563857e-66	0,494554254	0.568	0.229	1,01E-61	11	CT
81	ENSGALG00000003670	MAFB	MAF bZIP transcription factor B [Source:NCBI gene;Acc:419173]	1.92402702277848e-56	0,469378011	0.432	0.155	4,69E-52	11	CT
602	ENSGALG00000037953	TUBA1A	tubulin alpha 1a [Source:NCBI gene;Acc:429035]	1.41284296815563e-44	0,406265606	0.771	0.504	3,44E-40	11	CT
341	ENSGALG00000015089	GAP43	growth associated protein 43 [Source:NCBI gene;Acc:427955]	1.48536629975356e-117	0,400599978	0.261	0.036	3,62E-113	11	CT
691	ENSGALG00000043610	ENSGALG0000004361(	alpha-tectorin-like [Source:NCBI gene;Acc:100857892]	1.01161830087098e-126	0,395769882	0.369	0.064	2,46E-122	11	CT
148	ENSGALG00000006583	LSP1P1	lymphocyte-specific protein 1 pseudogene 1 [Source:NCBI gene;Acc:374254]	7.35521895521998e-62	0,395129438	0.548	0.216	1,79E-57	11	CT
261	ENSGALG00000011274	DCN	decorin [Source:NCBI gene;Acc:417892]	7.35087102526151e-47	0,393864458	0.952	0.698	1,79E-42	11	CT
332	ENSGALG00000014910	COLEC12	collectin subfamily member 12 [Source:NCBI gene;Acc:421061]	8.57927202252364e-46	0,377908966	0.432	0.174	2,09E-41	11	CT
194	ENSGALG00000008542	ADD3	adducin 3 (gamma) [Source:NCBI gene;Acc:373892]	2.55288835642505e-43	0,357348584	0.543	0.263	6,22E-39	11	CT
555	ENSGALG00000034986	HEYL	hes related family bHLH transcription factor with YRPW motif-like [Source:NCBI gene;Acc:771101]	1.48795705715564e-82	0,34373322	0.344	0.08	3,62E-78	11	CT
700	ENSGALG00000045199	ENSGALG0000004519	dispanin subfamily A member 2b-like [Source:NCBI gene;Acc:107053353]	1.53070527685491e-73	0,342876897	0.369	0.098	3,73E-69	11	CT
242	ENSGALG00000010461	EBF3	early B cell factor 3 [Source:NCBI gene;Acc:395175]	1.10107524021869e-39	0,329446279	0.646	0.344	2,68E-35	11	CT
563	ENSGALG00000035584	COL3A1	collagen type III alpha 1 chain [Source:NCBI gene;Acc:396340]	1.22008588450817e-37	0,317902395	0.872	0.522	2,97E-33	11	CT
213	ENSGALG00000009241	SFRP2	secreted frizzled related protein 2 [Source:NCBI gene;Acc:395546]	1.95793332168675e-14	0,30500255	0.415	0.259	4,77E-10	11	CT
378	ENSGALG00000016473	OSR1	odd-skipped related transciption factor 1 [Source:NCBI gene;Acc:100316920]	2.81882303409912e-82	0,300534987	0.334	0.074	6,87E-78	11	CT
585	ENSGALG00000036847	PLAC9	placenta specific 9 [Source:NCBI gene;Acc:423636]	7.15315492895487e-58	0,299123964	0.407	0.129	1,74E-53	11	CT
447	ENSGALG00000026970	ENSGALG0000002697(	interferon-induced transmembrane protein 1-like [Source:NCBI gene;Acc:770612]	5.80877293285741e-50	0,280858477	0.425	0.155	1,41E-45	11	CT
286	ENSGALG00000012017	DAAM1	dishevelled associated activator of morphogenesis 1 [Source:NCBI gene;Acc:423532]	2.41103942133076e-29	0,258144797	0.44	0.22	5,87E-25	11	CT
458	ENSGALG00000027891	NREP	neuronal regeneration related protein [Source:NCBI gene;Acc:396353]	1.06936015247797e-16	0,252415797	0.704	0.554	2,60E-12	11	CT
228	ENSGALG00000009844	ACTC1	actin alpha cardiac muscle 1 [Source:NCBI gene;Acc:423298]	3.80949369639664e-245	2,42919324	0.952	0.116	9,28E-241	15	M
156	ENSGALG00000006835	TNNC2	troponin C2, fast skeletal type [Source:NCBI gene;Acc:396434]	0	1,931698319	0.903	0.067	0	15	M
8	ENSGALG00000000313	TNNI1	troponin I type 1 (skeletal, slow) [Source:NCBI gene;Acc:421161]	0	1,83129066	0.883	0.035	0	15	M
22	ENSGALG00000001459	TNNC1	troponin C1, slow skeletal and cardiac type [Source:NCBI gene;Acc:396032]	0	1,785221883	0.883	0.037	0	15	M
251	ENSGALG00000011086	ACTA1	actin alpha 1, skeletal muscle [Source:NCBI gene;Acc:421534]	0	1,697813016	0.793	0.038	0	15	M
727	ENSGALG00000053246	OCM2	oncomodulin 2 [Source:NCBI gene;Acc:396531]	9.43919258841803e-244	1,647033481	0.766	0.071	2,30E-239	15	M
49	ENSGALG00000002907	MYL1	myosin, light chain 1, alkali; skeletal, fast [Source:NCBI gene;Acc:396470]	0	1,630655295	0.848	0.029	0	15	M
28	ENSGALG00000001793	MYL10	myosin, light chain 10, regulatory [Source:NCBI gene;Acc:417506]	0	1,604056883	0.717	0.019	0	15	M
480	ENSGALG00000029203	TNNT1	troponin T1, slow skeletal type [Source:NCBI gene;Acc:396009]	0	1,316900942	0.717	0.021	0	15	M
321	ENSGALG00000014463	ACTN2	actinin alpha 2 [Source:NCBI gene;Acc:396263]	0	1,210447991	0.669	0.014	0	15	M
149	ENSGALG00000006591	TNNI2	troponin I2, fast skeletal type [Source:NCBI gene;Acc:396386]	0	1,201341186	0.724	0.016	0	15	M
513	ENSGALG00000031593	TMSB15B	thymosin beta 15B [Source:NCBI gene;Acc:100502566]	3.01860784890416e-51	1,190515508	0.917	0.552	7,35E-47	15	M
103	ENSGALG00000004518	BHMT2	betaine--homocysteine S-methyltransferase 2 [Source:NCBI gene;Acc:416371]	1.04684293127582e-208	1,149633897	0.614	0.051	2,55E-204	15	M
299	ENSGALG00000012712	RBM24	RNA binding motif protein 24 [Source:NCBI gene;Acc:420846]	2.46027451806984e-75	1,137209915	0.69	0.15	5,99E-71	15	M
311	ENSGALG00000013239	ENSGALG00000013239		2.02536129705077e-85	1,10768699	0.6	0.116	4,93E-81	15	M
451	ENSGALG00000027323	MYH1D	myosin, heavy chain 1D, skeletal muscle (similar to human myosin, heavy chain 1, skeletal muscle, a	0	1,062182552	0.572	0.008	0	15	M
145	ENSGALG00000006572	TNNT3	troponin T3, fast skeletal type [Source:NCBI gene;Acc:395761]	0	1,061090593	0.683	0.011	0	15	M

666	ENSGALG00000042257	MYH1F	myosin, heavy chain 1F, skeletal muscle (similar to human myosin, heavy chain 1, skeletal muscle, a	0	0,960275871	0.497	0.007	0	15	M
717	ENSGALG00000051776	ENSGALG00000051776		1.65421820710932e-136	0,896257707	0.407	0.033	4,03E-132	15	M
67	ENSGALG00000003521	TPM1	tropomyosin 1 (alpha) [Source:NCBI gene;Acc:396366]	1.74304133580569e-50	0,877488114	0.766	0.299	4,25E-46	15	M
266	ENSGALG00000011306	DES	desmin [Source:NCBI gene;Acc:395906]	5.88408241360416e-281	0,848927351	0.524	0.026	1,43E-276	15	M
272	ENSGALG00000011511	CKB	creatine kinase B [Source:NCBI gene;Acc:396248]	3.91114307631224e-45	0,780163888	0.503	0.131	9,53E-41	15	M
104	ENSGALG00000004582	MYL2	myosin, light chain 2, regulatory, cardiac, slow [Source:NCBI gene;Acc:416874]	0	0,763616576	0.448	0.006	0	15	M
517	ENSGALG00000031826	MYOT	myotilin [Source:NCBI gene;Acc:416299]	0	0,739916456	0.434	0.005	0	15	M
205	ENSGALG00000008945	NEXN	nexilin F-actin binding protein [Source:NCBI gene;Acc:424552]	4.1572454297076e-100	0,697280537	0.455	0.055	1,01E-95	15	M
474	ENSGALG00000028612	MYH1G	myosin, heavy chain 1G, skeletal muscle (similar to human myosin, heavy chain 1, skeletal muscle, a	0	0,673448048	0.303	0.004	0	15	M
302	ENSGALG00000012783	MYBPC1	myosin binding protein C, slow type [Source:NCBI gene;Acc:418099]	0	0,664235559	0.359	0.003	0	15	M
724	ENSGALG00000053046	ENSGALG00000053046		6.77415479230231e-146	0,657555705	0.434	0.035	1,65E-141	15	M
7	ENSGALG00000000302	TNNT2	troponin T2, cardiac type [Source:NCBI gene;Acc:396433]	0	0,645074332	0.366	0.008	0	15	M
473	ENSGALG00000028567	MYL9	myosin, light chain 9, regulatory [Source:NCBI gene;Acc:396215]	1.4658228779742e-52	0,643158474	0.559	0.142	3,57E-48	15	M
661	ENSGALG00000042020	CG-16	galectin CG-16 [Source:NCBI gene;Acc:404269]	3.67310845931942e-69	0,639895022	0.476	0.084	8,95E-65	15	M
29	ENSGALG00000001926	HSPB1	heat shock protein family B (small) member 1 [Source:NCBI gene;Acc:396227]	2.87099753037848e-130	0,621191722	0.462	0.044	6,99E-126	15	M
193	ENSGALG00000008486	CACNA2D1	calcium voltage-gated channel auxiliary subunit alpha2delta 1 [Source:NCBI gene;Acc:768444]	3.82397685258638e-49	0,618989289	0.324	0.053	9,31E-45	15	M
127	ENSGALG00000006025	XIRP1	xin actin binding repeat containing 1 [Source:NCBI gene;Acc:374266]	0	0,618946646	0.317	0.003	0	15	M
678	ENSGALG00000043035	SHISA2	shisa family member 2 [Source:NCBI gene;Acc:395162]	1.49799600220959e-30	0,588725624	0.428	0.128	3,65E-26	15	M
284	ENSGALG00000011991	MYOZ2	myozenin 2 [Source:NCBI gene;Acc:422682]	0	0,584031457	0.421	0.005	0	15	M
376	ENSGALG00000016342	MYOM2	myomesin 2 [Source:NCBI gene;Acc:396034]	0	0,554677891	0.4	0.01	0	15	M
117	ENSGALG00000005448	MYL3	myosin, light chain 3, alkali; ventricular, skeletal, slow [Source:NCBI gene;Acc:396067]	0	0,548601469	0.248	0.005	0	15	M
542	ENSGALG00000033677	AHCY	adenosylhomocysteinase [Source:NCBI gene;Acc:419146]	2.00311166903665e-28	0,53164784	0.669	0.311	4,88E-24	15	M
325	ENSGALG00000014645	MEF2C	myocyte enhancer factor 2C [Source:NCBI gene;Acc:769007]	7.65559106983121e-83	0,529184976	0.303	0.029	1,86E-78	15	M
208	ENSGALG00000009081	ZNF106	zinc finger protein 106 [Source:NCBI gene;Acc:423234]	2.79273168362389e-72	0,524572032	0.414	0.06	6,80E-68	15	M
737	ENSGALG00000054420	TUBB2A	tubulin, beta 2A class IIa [Source:NCBI gene;Acc:768337]	4.43449408300379e-26	0,513622622	0.531	0.211	1,08E-21	15	M
234	ENSGALG00000010027	CFL2	cofilin 2 [Source:NCBI gene;Acc:423320]	2.75904947018895e-36	0,510244402	0.476	0.14	6,72E-32	15	M
35	ENSGALG00000002479	MAT1A	methionine adenosyltransferase 1A [Source:NCBI gene;Acc:423628]	0	0,502718122	0.331	0.007	0	15	M
60	ENSGALG00000003226	APOBEC2	apolipoprotein B mRNA editing enzyme catalytic subunit 2 [Source:NCBI gene;Acc:419915]	0	0,48460666	0.4	0.012	0	15	M
368	ENSGALG00000015935	SMYD1	SET and MYND domain containing 1 [Source:NCBI gene;Acc:373960]	9.81292275100682e-208	0,476391175	0.338	0.014	2,39E-203	15	M
3	ENSGALG00000000164	MYBPHL	myosin binding protein H like [Source:NCBI gene;Acc:421154]	0	0,45806977	0.359	0.01	0	15	M
328	ENSGALG00000014847	MYOM1	myomesin 1 [Source:NCBI gene;Acc:395805]	0	0,450939571	0.31	0.005	0	15	M
312	ENSGALG00000013414	PDLIM3	PDZ and LIM domain 3 [Source:NCBI gene;Acc:414873]	0	0,44457273	0.31	0.007	0	15	M
715	ENSGALG00000050058	REEP1	receptor accessory protein 1 [Source:NCBI gene;Acc:771776]	3.42381256993657e-130	0,437522371	0.359	0.026	8,34E-126	15	M
352	ENSGALG00000015358	MYH15	myosin, heavy chain 15 [Source:NCBI gene;Acc:395534]	0	0,427036444	0.276	0.004	0	15	M
387	ENSGALG00000016826	ADPRHL1	ADP-ribosylhydrolase like 1 [Source:NCBI gene;Acc:418741]	0	0,417890103	0.283	0.004	0	15	M
672	ENSGALG00000042784	TPPP	tubulin polymerization promoting protein [Source:NCBI gene;Acc:420800]	1.10076641905277e-251	0,417886311	0.248	0.006	2,68E-247	15	M
133	ENSGALG00000006216	MYOD1	myogenic differentiation 1 [Source:NCBI gene;Acc:374048]	4.19426852489388e-27	0,415910454	0.421	0.129	1,02E-22	15	M
301	ENSGALG00000012715	CAP2	cyclase associated actin cytoskeleton regulatory protein 2 [Source:NCBI gene;Acc:420847]	7.5897126330855e-52	0,413327854	0.331	0.053	1,85E-47	15	M
644	ENSGALG00000040995	ENSGALG00000040995		2.04884867283739e-124	0,406501163	0.31	0.021	4,99E-120	15	M
429	ENSGALG00000026081	RBM38	RNA binding motif protein 38 [Source:NCBI gene;Acc:768866]	8.87314833347052e-46	0,404161499	0.338	0.061	2,16E-41	15	M
646	ENSGALG00000041327	TRIM55	tripartite motif containing 55 [Source:NCBI gene;Acc:420166]	1.38688627987635e-233	0,393143122	0.283	0.009	3,38E-229	15	M
116	ENSGALG00000005442	PALMD	palmdelphin [Source:NCBI gene;Acc:424475]	4.03261896048806e-60	0,386610105	0.317	0.043	9,82E-56	15	M
229	ENSGALG00000009856	GNS	glucosamine (N-acetyl)-6-sulfatase [Source:NCBI gene;Acc:417829]	5.22834518993488e-29	0,386603702	0.303	0.071	1,27E-24	15	M
547	ENSGALG00000034100	UACA	uveal autoantigen with coiled-coil domains and ankyrin repeats [Source:NCBI gene;Acc:415569]	1.58784801186148e-09	0,381472909	0.124	0.034	3,87E-05	15	M
313	ENSGALG00000013604	VIP	vasoactive intestinal peptide [Source:NCBI gene;Acc:396323]	1.11999120820918e-173	0,380946769	0.145	0.003	2,73E-169	15	M
669	ENSGALG00000042492	PITX2	paired like homeodomain 2 [Source:NCBI gene;Acc:395862]	3.70234233382837e-23	0,374942202	0.324	0.093	9,02E-19	15	M
320	ENSGALG00000014442	GAPDH	glyceraldehyde-3-phosphate dehydrogenase [Source:NCBI gene;Acc:374193]	6.70541902237905e-10	0,356969486	0.952	0.941	1,63E-05	15	M
386	ENSGALG00000016702	PPP2R3B	protein phosphatase 2 regulatory subunit B''beta [Source:NCBI gene;Acc:418670]	1.33006261045282e-78	0,354334688	0.255	0.022	3,24E-74	15	M
422	ENSGALG00000025842	TPM2	tropomyosin 2 [Source:NCBI gene;Acc:396430]	2.72155698489632e-14	0,351155539	0.421	0.198	6,63E-10	15	M
552	ENSGALG00000034493	DCLK3	doublecortin like kinase 3 [Source:NCBI gene;Acc:776499]	4.27151436030895e-76	0,345792342	0.117	0.005	1,04E-71	15	M
719	ENSGALG00000051939	ENSGALG00000051939		9.64632525444325e-145	0,344545789	0.241	0.011	2,35E-140	15	M
614	ENSGALG00000038936	TMEM182	transmembrane protein 182 [Source:NCBI gene;Acc:418721]	0	0,343727569	0.234	0.002	0	15	M
30	ENSGALG00000002125	TMOD1	tropomodulin 1 [Source:NCBI gene;Acc:395883]	2.08937638307776e-174	0,339810617	0.276	0.011	5,09E-170	15	M
706	ENSGALG00000047289	TNFAIP8L3	TNF alpha induced protein 8 like 3 [Source:NCBI gene;Acc:427499]	5.1302462528289e-18	0,33474293	0.29	0.089	1,25E-13	15	M
413	ENSGALG00000022980	CDKN1B	cyclin dependent kinase inhibitor 1B [Source:NCBI gene;Acc:374106]	9.41921997925956e-23	0,332312541	0.407	0.138	2,29E-18	15	M

712	ENSGALG00000048310	ENSGALG00000048310		3.85210587617385e-84	0,330867906	0.2	0.013	9,38E-80	15	M
124	ENSGALG00000005843	EEF1A2	eukaryotic translation elongation factor 1 alpha 2 [Source:NCBI gene;Acc:419244]	3.6368964311319e-278	0,328644509	0.262	0.006	8,86E-274	15	M
568	ENSGALG00000035994	ENSGALG0000003599[illegible]	probable acyl coa dehydrogenase 6-like [Source:NCBI gene;Acc:420562]	1.814610267039e-37	0,326156507	0.317	0.063	4,42E-33	15	M
278	ENSGALG00000011695	HIPK3	homeodomain interacting protein kinase 3 [Source:NCBI gene;Acc:421597]	1.46959436407649e-13	0,32065283	0.29	0.108	3,58E-09	15	M
503	ENSGALG00000030985	MYH7B	myosin, heavy chain 7B, cardiac muscle, beta [Source:NCBI gene;Acc:395279]	5.81099959252741e-153	0,312447979	0.124	0.002	1,42E-148	15	M
723	ENSGALG00000052907	ENSGALG00000052907		3.16132252658874e-97	0,29964671	0.214	0.012	7,70E-93	15	M
296	ENSGALG00000012691	ALDH1L2	aldehyde dehydrogenase 1 family member L2 [Source:NCBI gene;Acc:418078]	8.23963086112837e-14	0,290868601	0.255	0.088	2,01E-09	15	M
130	ENSGALG00000006190	FHL1	four and a half LIM domains 1 [Source:NCBI gene;Acc:770787]	4.16236295149257e-25	0,288555934	0.193	0.035	1,01E-20	15	M
380	ENSGALG00000016500	FKBP1B	FK506 binding protein 1B [Source:NCBI gene;Acc:395254]	1.83843467408899e-41	0,276825728	0.228	0.031	4,48E-37	15	M
489	ENSGALG00000029834	AIF1L	allograft inflammatory factor 1-like [Source:NCBI gene;Acc:417179]	5.52218613348184e-18	0,269492266	0.283	0.086	1,34E-13	15	M
621	ENSGALG00000039452	GJA5	gap junction protein alpha 5 [Source:NCBI gene;Acc:396502]	3.5661328233109e-138	0,259485515	0.166	0.005	8,69E-134	15	M
587	ENSGALG00000037050	FABP3	fatty acid binding protein 3 [Source:NCBI gene;Acc:419557]	6.78304332373964e-77	0,258620356	0.262	0.023	1,65E-72	15	M
578	ENSGALG00000036471	RFTN2	raftlin family member 2 [Source:NCBI gene;Acc:424062]	4.21758121884091e-15	0,250215229	0.262	0.085	1,03E-10	15	M

Supplementary Table 2
Differentially-expressed genes in the CT/M cells at E4
List of the genes differentially expressed in the CT/M cells compared to the combined CT and muscle populations at E4.
In green, up-regulated genes; in red, down-regulated genes.
In the "Marker type" column, "CT" or "M" means that the gene is respectively identified as a CT or a muscle marker in Supplementary Table 1.

	ensembl_gene_id	external_gene_name	description	p_val	avg_logFC	pct.1	pct.2	p_val_adj	Marker type
41	ENSGALG00000042871	SLIT1	slit guidance ligand 1 [Source:NCBI gene;Acc:395293]	1.60020002558476e-07	0,74283005	0.538	0.167	0,003897447	M
24	ENSGALG00000029401	SIX1	SIX homeobox 1 [Source:NCBI gene;Acc:693262]	1.36203421292892e-07	0,675256759	0.577	0.21	0,003317371	M
22	ENSGALG00000026276	TCF15	transcription factor 15 (basic helix-loop-helix) [Source:NCBI gene;Acc:395788]	8.8150005923494e-23	0,661174402	0.5	0.057	2,15E-18	M
42	ENSGALG00000043204	PAX7	paired box 7 [Source:NCBI gene;Acc:395942]	1.24409726254136e-211	0,582528709	0.577	0.006	3,03E-207	M
9	ENSGALG00000009612	TGFB2	transforming growth factor beta 2 [Source:NCBI gene;Acc:421352]	4.23447895865566e-12	0,51572834	0.577	0.124	1,03E-07	M + CT
33	ENSGALG00000038515	LFNG	LFNG O-fucosylpeptide 3-beta-N-acetylglucosaminyltransferase [Source:NCBI gene;Acc:395790]	1.48198775512662e-18	0,432838248	0.385	0.04	3,61E-14	M
8	ENSGALG00000008072	CHRDL1	chordin like 1 [Source:NCBI gene;Acc:373985]	2.45364077691003e-17	0,387527316	0.385	0.043	5,98E-13	M + CT
34	ENSGALG00000040021	HOXA11	homeobox A11 [Source:NCBI gene;Acc:395327]	1.10561303418951e-06	0,372425467	0.346	0.086	0,026928311	M + CT
4	ENSGALG00000003642	PDGFA	platelet derived growth factor subunit A [Source:NCBI gene;Acc:374196]	1.71771342572974e-21	0,312061626	0.346	0.028	4,18E-17	M
36	ENSGALG00000041121	SLIT2	slit guidance ligand 2 [Source:NCBI gene;Acc:373967]	7.97736553371562e-08	0,263756387	0.346	0.073	0,001942967	M

Supplementary Table 2

Differentially-expressed genes in the CT/M cells at E6

List of the genes differentially expressed in the CT/M cells compared to the combined CT and muscle populations at E6.

In green, up-regulated genes; in red, down-regulated genes.

In the “Marker type” column, “CT” or “M” means that the gene is respectively identified as a CT or a muscle marker in Supplementary Table 1.

	ensembl_gene_id	external_gene_name	description	p_val	avg_logFC	pct.1	pct.2	p_val_adj	Marker type
53	ENSGALG00000042492	PITX2	paired like homeodomain 2 [Source:NCBI gene;Acc:395862]	4.72811729184315e-125	0,968209639	0.759	0.088	1,15E-120	M
33	ENSGALG00000015419	PENK	proenkephalin [Source:NCBI gene;Acc:421131]	2.15342013612674e-48	0,943825102	0.398	0.061	5,24E-44	M
25	ENSGALG00000012712	RBM24	RNA binding motif protein 24 [Source:NCBI gene;Acc:420846]	7.02306908179211e-96	0,807582096	0.564	0.06	1,71E-91	M
9	ENSGALG00000006216	MYOD1	myogenic differentiation 1 [Source:NCBI gene;Acc:374048]	8.47814884812583e-132	0,731034261	0.556	0.039	2,06E-127	M
40	ENSGALG00000028543	FGFR4	fibroblast growth factor receptor 4 [Source:NCBI gene;Acc:395603]	1.87521933312838e-122	0,667416501	0.737	0.082	4,57E-118	M
24	ENSGALG00000011511	CKB	creatine kinase B [Source:NCBI gene;Acc:396248]	5.74039007142911e-45	0,653753638	0.534	0.119	1,40E-40	M
6	ENSGALG00000003642	PDGFA	platelet derived growth factor subunit A [Source:NCBI gene;Acc:374196]	7.08017486764191e-78	0,58136215	0.586	0.083	1,72E-73	M
39	ENSGALG00000027887	C1QTNF3	C1q and tumor necrosis factor related protein 3 [Source:NCBI gene;Acc:427430]	1.59974702624866e-76	0,544839258	0.451	0.047	3,90E-72	M
41	ENSGALG00000029401	SIX1	SIX homeobox 1 [Source:NCBI gene;Acc:693262]	5.3442043481086e-36	0,499661023	0.714	0.224	1,30E-31	M + CT
54	ENSGALG00000043204	PAX7	paired box 7 [Source:NCBI gene;Acc:395942]	3.07744461286103e-137	0,415713003	0.526	0.032	7,50E-133	M
17	ENSGALG00000009612	TGFB2	transforming growth factor beta 2 [Source:NCBI gene;Acc:421352]	5.39511247107488e-24	0,395684552	0.737	0.286	1,31E-19	M + CT
42	ENSGALG00000031593	TMSB15B	thymosin beta 15B [Source:NCBI gene;Acc:100502566]	5.71054681275557e-10	0,375086566	0.917	0.804	1,39E-05	M
50	ENSGALG00000040465	ZEB2	zinc finger E-box binding homeobox 2 [Source:NCBI gene;Acc:424306]	3.4597119543024e-50	0,335128122	0.444	0.071	8,43E-46	M
12	ENSGALG00000008094	HSPD1	heat shock protein family D (Hsp60) member 1 [Source:NCBI gene;Acc:424059]	1.1747745268059e-10	0,32552865	0.865	0.625	2,86E-06	M
10	ENSGALG00000006835	TNNC2	troponin C2, fast skeletal type [Source:NCBI gene;Acc:396434]	8.83786248736828e-52	0,323379667	0.451	0.07	2,15E-47	M
43	ENSGALG00000032329	NPM3	nucleophosmin/nucleoplasmin 3 [Source:NCBI gene;Acc:770430]	1.34883628793817e-24	0,322624238	0.451	0.13	3,29E-20	M
18	ENSGALG00000010175	HSP90AB1	heat shock protein 90 alpha family class B member 1 [Source:NCBI gene;Acc:396188]	3.29797003971847e-13	0,308055966	0.977	0.815	8,03E-09	M
35	ENSGALG00000017179	PDGFD	platelet derived growth factor D [Source:NCBI gene;Acc:418978]	1.64263449477098e-28	0,288090927	0.481	0.126	4,00E-24	M
36	ENSGALG00000017644	COTL1	coactosin like F-actin binding protein 1 [Source:NCBI gene;Acc:768420]	1.06784609628289e-10	0,278735806	0.647	0.384	2,60E-06	M
37	ENSGALG00000025958	U3	Small nucleolar RNA U3 [Source:RFAM;Acc:RF00012]	3.84088868596491e-16	0,266706303	0.511	0.206	9,35E-12	M
38	ENSGALG00000026383	TMSB4X	thymosin beta 4, X-linked [Source:NCBI gene;Acc:408047]	5.12373896201496e-08	0,262025064	0.992	0.977	0,001247938	M + CT
45	ENSGALG00000032930	NCL	nucleolin [Source:NCBI gene;Acc:396201]	1.37707522219677e-10	0,259646791	0.97	0.922	3,35E-06	M
20	ENSGALG00000010745	HMGB2	high mobility group box 2 [Source:NCBI gene;Acc:396482]	9.19841408695929e-08	0,259541694	0.774	0.549	0,002240366	M + CT
27	ENSGALG00000013239			7.24247772335581e-17	0,25729827	0.571	0.231	1,76E-12	M
52	ENSGALG00000041555	COL1A1	collagen type I alpha 1 chain [Source:NCBI gene;Acc:395532]	1.12030079969768e-06	-0,294750404	0.91	0.924	0,027286046	CT
21	ENSGALG00000010818	PLPP3	phospholipid phosphatase 3 [Source:NCBI gene;Acc:424666]	1.0101761464672e-07	-0,307271539	0.12	0.33	0,002460385	CT
28	ENSGALG00000014233	FBLN1	fibulin 1 [Source:NCBI gene;Acc:373979]	1.38495302808083e-06	-0,317247671	0.271	0.426	0,033731916	CT
14	ENSGALG00000008298	LRRC17	leucine rich repeat containing 17 [Source:NCBI gene;Acc:395440]	1.03392730242788e-08	-0,357108192	0.135	0.361	0,000251823	CT
11	ENSGALG00000007000	NR2F2	nuclear receptor subfamily 2 group F member 2 [Source:NCBI gene;Acc:386585]	2.16061889005824e-07	-0,416096643	0.256	0.463	0,005262403	CT
2	ENSGALG00000002546	COL5A1	collagen type V alpha 1 chain [Source:NCBI gene;Acc:395568]	2.67346479235358e-10	-0,423052863	0.579	0.74	6,51E-06	CT
13	ENSGALG00000008253	TBX5	T-box 5 [Source:NCBI gene;Acc:373988]	1.66907085390869e-13	-0,429851158	0.271	0.582	4,07E-09	CT
16	ENSGALG00000009274	HOXD12	homeobox D12 [Source:NCBI gene;Acc:396178]	4.00545715533294e-07	-0,430506869	0.241	0.436	0,009755691	CT
32	ENSGALG00000015193	CCDC80	coiled-coil domain containing 80 [Source:NCBI gene;Acc:395074]	1.58259687955259e-10	-0,464952383	0.301	0.54	3,85E-06	CT
23	ENSGALG00000011274	DCN	decorin [Source:NCBI gene;Acc:417892]	5.49231928586092e-13	-0,561425767	0.361	0.634	1,34E-08	CT
26	ENSGALG00000012834	AKR1D1	aldo-keto reductase family 1 member D1 [Source:NCBI gene;Acc:418107]	1.66206126306059e-07	-0,591302226	0.504	0.633	0,004048116	CT
48	ENSGALG00000039985	CDH11	cadherin 11 [Source:NCBI gene;Acc:415797]	2.59278172018454e-20	-0,634295253	0.398	0.725	6,31E-16	CT
3	ENSGALG00000003193	CRABP-I	cellular retinoic acid binding protein 1 [Source:NCBI gene;Acc:374211]	7.06132984900811e-14	-0,901123182	0.346	0.617	1,72E-09	CT

Supplementary Table 2

Differentially-expressed genes in the CT/M cells at E10

List of the genes differentially expressed in the CT/M cells compared to the combined CT and muscle populations at E10.

In green, up-regulated genes; in red, down-regulated genes.

In the “Marker type” column, “CT” or “M” means that the gene is respectively identified as a CT or a muscle marker in Supplementary Table 1.

	ensembl_gene_id	external_gene_name	description	p_val	avg_logFC	pct.1	pct.2	p_val_adj	Marker type
6	ENSGALG00000027887	C1QTNF3	C1q and tumor necrosis factor related protein 3 [Source:NCBI gene;Acc:427430]	1.05233077990412e-74	0,35272088	0.534	0.231	2,56E-70	M
3	ENSGALG00000006216	MYOD1	myogenic differentiation 1 [Source:NCBI gene;Acc:374048]	1.17975537153951e-239	0,307981904	0.574	0.123	2,87E-235	M
10	ENSGALG00000043204	PAX7	paired box 7 [Source:NCBI gene;Acc:395942]	1.43426716373098e-206	0,272881541	0.422	0.083	3,49E-202	M
8	ENSGALG00000039985	CDH11	cadherin 11 [Source:NCBI gene;Acc:415797]	3.76082136792317e-21	-0,268870628	0.362	0.519	9,16E-17	CT
5	ENSGALG00000015908	COL12A1	collagen type XII alpha 1 chain [Source:NCBI gene;Acc:395875]	1.35360515126287e-10	-0,280083667	0.215	0.314	3,30E-06	CT
2	ENSGALG00000003193	CRABP-I	cellular retinoic acid binding protein 1 [Source:NCBI gene;Acc:374211]	3.06215651991207e-14	-0,290385609	0.194	0.314	7,46E-10	CT
4	ENSGALG00000011274	DCN	decorin [Source:NCBI gene;Acc:417892]	7.50759284025687e-28	-0,41112646	0.653	0.726	1,83E-23	CT
1	ENSGALG00000002546	COL5A1	collagen type V alpha 1 chain [Source:NCBI gene;Acc:395568]	5.78626863490225e-36	-0,441903009	0.625	0.737	1,41E-31	CT
7	ENSGALG00000035584	COL3A1	collagen type III alpha 1 chain [Source:NCBI gene;Acc:396340]	3.47592519408835e-29	-0,452300505	0.378	0.556	8,47E-25	CT
9	ENSGALG00000041555	COL1A1	collagen type I alpha 1 chain [Source:NCBI gene;Acc:395532]	3.80457290749328e-27	-0,462427214	0.752	0.819	9,27E-23	CT

Supplementary Table 3

List of reagents and resources

REAGENT TYPE OR RESOURCE	DESIGNATION	SOURCE	Identifiers	Lot number	Additional information
Primary antibody	Mouse monoclonal IgG2b anti-MyHC (MF20)	DSHB	Cat. # MF 20<br>RRID:AB_2147781	Produced in the lab	IF (undiluted)
Primary antibody	Mouse monoclonal IgG1 anti-Myod	BD Biosciences	Cat. # 554130<br>RRID:AB_395255	9011506	IF (1:500)
Primary antibody	Mouse monoclonal IgG1 anti-Myog	DSHB	Cat. # F5D<br>RRID:AB_2146602	Produced in the lab	IF (1:30)
Primary antibody	Mouse monoclonal IgG1 anti-Pax7	DSHB	Cat. # AB_PAX7<br>RRID:AB_528428	20ea1/24/19	IF (1:200)
Primary antibody	Mouse monoclonal IgG1 anti-quail nuclei (QCPN)	DSHB	Cat. # QCPN<br>RRID:AB_531886	Produced in the lab	IF (undiluted)
Primary antibody	Rabbit polyclonal anti-β-gal	MP Biomedicals	Cat. # MP 55976<br>RRID:AB_2687418	6825	IF (1:1500)
Primary antibody	Rabbit polyclonal anti-Collagen XII	Clone #522 from Manuel Koch	Koch et al., 1992	N/A	IF (1:100)
Primary antibody	Rabbit polyclonal anti-Myog	From Christophe Marcelle	Marceau et al., 2008	HL1510	IF (Undiluted)
Primary antibody	Rabbit polyclonal anti-PSMAD1/5/9	Cell Signaling	Cat. # 9516<br>RRID:AB_491015	9	IF (1:100)
Primary antibody	Rabbit polyclonal anti-TCF4	Cell Signaling	Cat. # 2569<br>RRID:AB_2199816	4	IF (1:100)
Primary antibody	Rabbit polyclonal anti-Tomato	TAKARA	Cat. # 632496<br>RRID:AB_10013483	1612022	IF (1:400)
Primary antibody	Chicken polyclonal anti-GFP	Abcam	Cat. # ab13970<br>RRID:AB_300798	GR3190550-33	IF (1:1000)
Secondary antibody	Alexa Fluor® 555 Goat Anti-Mouse IgG	Invitrogen	Cat. # A21422<br>RRID:AB_141822	514956	IF (1:200)
Secondary antibody	Alexa Fluor®488 AffiniPure Goat Anti-Mouse IgG1(γ1)	Jackson ImmunoResearch Labs	Cat. # 115-545-205<br>RRID:AB_2338854	101249	IF (1:500)
Seconday antibody	Alexa Fluor® 633 Goat Anti-Mouse IgG1(γ1)	Thermo Fisher Scientific	Cat. # A-21126<br>RRID:AB_2535768	1977327	IF (1:500)
Secondary antibody	Alexa Fluor® 647 Goat Anti-Mouse IgG1(γ1)	Thermo Fisher Scientific	Cat. # Z 25008<br>RRID:AB_2535768	1915925	IF (1:500)
Secondary antibody	Cy3-AffiniPure Goat Anti-Mouse IgG1(γ1)	Jackson ImmunoResearch Labs	Cat. # 115-165-205<br>RRID:AB_2338694	135078	IF (1:500)
Secondary antibody	Alexa Fluor® 488 Goat Anti-Mouse IgG2b(γ2)	Invitrogen	Cat. # A21141<br>RRID:AB_141626	2228625 / 1723667	IF (1:200)
Seconday antibody	Alexa Fluor® 633 Goat Anti-Mouse IgG2b (γ2b)	Thermo Fisher Scientific	Cat. # A-21146<br>RRID:AB_2535782	1786287	IF (1:500)
Secondary antibody	Alexa Fluor® 647 Goat Anti-Mouse IgG2b (γ2b)	Thermo Fisher Scientific	Cat. # A-21242<br>RRID:AB_2535782	2155295	IF (1:500)
Secondary antibody	Alexa Fluor® 488 F(ab')2 Fragment of Goat Anti-Rabbit IgG	Thermo Fisher Scientific	Cat. # A-11070<br>RRID:AB_2534114	1907301	IF (1:500)
Secondary antibody	Alexa Fluor® 555 F(ab')2 Fragment of Goat Anti-Rabbit IgG	Thermo Fisher Scientific	Cat. # A-21430<br>RRID:AB_2535851	2184320	IF (1:500)
Secondary antibody	Alexa Fluor® 633 F(ab')2 Fragment of Goat Anti-Rabbit IgG	Thermo Fisher Scientific	Cat. # A-21072<br>RRID:AB_2535733	1839600	IF (1:500)
Secondary antibody	Alexa Fluor® 488 Goat Anti-Chicken IgG (H+L)	Thermo Fisher Scientific	Cat. # A-11039<br>RRID:AB_2534096	16911381 / 1812246	IF (1:500)
DNA stain	DAPI	Sigma-Aldrich	Cat. # D9542	0097M4485V	IF (1:1000)
Chemical	EdU	Invitrogen	Cat. # A10044		5 mg/ml

Detection kit	Click-iT Plus EdU Cell Proliferation Kit for Imaging	Invitrogen	Cat. # C10639		N/A
Transfection kit	Calcium phosphate Transfection kit	Invitrogen	Cat. # 440052	Multiple lots	N/A
PCR kit	SYBR Green PCR Master mix	Life Technology	Cat. # 4385614	Multiple lots	N/A
Chemical	Tamoxifen	Sigma-Aldrich	Cat. # T5648	WXBC7537V	5 mg/mice
genetic reagent (*M. musculus*)	$Osr1^{GCE}$ ($Osr1^{eGFPCreERt2}$)	PMID: 18485340	MGI:3827104		Mugford et al. 2008
genetic reagent (*M. musculus*)	*Pax3-Cre*	PMID: 15882581	MGI:3573783		Engleka et al., 2005
genetic reagent (*M. musculus*)	*Tg:Scx-Cre*	PMID: 20059955	MGI:5317938		Blitz et al. 2009
genetic reagent (*M. musculus*)	*Tg:Wnt1Cre*	PMID:9843687	MGI:2386570		Danielian et al., 1998
genetic reagent (*M. musculus*)	$Pax7^{GPL}$	PMID: 23623977	MGI:3850147		Sambasivan et al. 2013
genetic reagent (*M. musculus*)	$Rosa26^{Tom}$ (*Ai9*)	PMID: 20023653	MGI:3809523		Madisen et al., 2010
genetic reagent (*M. musculus*)	$Rosa26^{Fucci2aR}$	PMID: 25486356	MGI:5645798		Mort et al. 2014
genetic reagent (*M. musculus*)	$Rosa26^{loxP\text{-}stop\text{-}loxHTB}$	PMID: 23671081	MGI:5518925		Li et al. 2013
genetic reagent (*M. musculus*)	*Tg:Scx-GFP*	PMID:17497702	MGI:3717419		Pryce et al., 2007

Supplementary Table 4

Primers used for Real-time quantitative PCR.

Genes	Forward primers	Reverse primers	Accession No.
COL3A1	5’-GCGTCCTGTTGTGCCAAAA-3’	5’-GTTCATTCTTGCCGTGTTTCAA-3’	NM_205380.2
COL6A1	5’-GCATGCCTAAACAAGCGATGT-3’	5’-GGAACAACCCAAACCCAGATC-3’	NM_205107
GAPDH	5’-GCTGGAGGAACCCATGAAT-3’	5’-TCCCGCAATTTGGAGTAGCA-3’	NM_204305.1
ID1	5’-CCGGAGGGTCTCTAAAGTGG-3’	5’-GCAGGTCCCAGATGTAGTCG-3’	NM_204590.2
ID2	5’-GAAGAACGGCCTTTCGGAG-3’	5’-TCATGTTGTACAGCAGGCTCA-3’	NM_205002.1
ID3	5’-GCTGGAGGAACCCATGAATCT-3’	5’-TCCCGCAATTTGGAGTAGCA-3’	NM_204589.1
MYF5	5’-ACCAGAGACTCCCCAAAGTG-3’	5’-TCGATGTACCTGATGGCGTT-3’	NM_001030363.1
MYHC	5’-TGACAACTCCTCACGCTTTG-3’	5’- CTCTGGCTTCTTGTTGGA-3’	NM_204228
MYOD1	5’-CGACAGCAGCTACTACACGGAAT-3’	5’-CTCTTCCCATGCTTTGGGTC-3’	NM_204214.2
MYOG	5’-AGGCTGAAGAAGGTGAACGAAG-3’	5’-CAGAGTGCTGCGTTTCAGAGC-3’	NM_204184.1
OSR1	5’-GTGCTGAATCTCCGACTTCTATGA-3’	5’-GTGTAAAATCTGAAGGGCAGGAA-3’	NM_001168709
PDGFRA	5’-GCTAGTGCTTGGTCGAATCC-3’	5’-TGTCCCTTCCACCACTTTTC-3’	NM_204749.2
RPS17	5’-ACCTGGTTCCTGCACAGGGCTT-3’	5’-TGCCTGAGCGGAGGAGCAAACA-3’	NM_204217.1
SCX	5’-CACCAACAGCGTCAACACC-3’	5’-CGTCTCGATCTTGGACAGC-3’	NM_204253.1
TCF4	5’-TCCGATTACAGACCTGAGCG-3’	5’-TGTTGATCAAGGCCAAAGCG-3’	NM_001206510.4
