# Supplementary material for: Unexpected contribution of fibroblasts to muscle lineage as a mechanism for limb muscle patterning
Source: Nat Commun. 2021 Jun 22;12:3851. doi: 10.1038/s41467-021-24157-x (PMC8219714; doi:10.1038/s41467-021-24157-x)
Supplement: Supplementary file 3 — Reporting Summary [file 41467_2021_24157_MOESM3_ESM.pdf]

nature research

Corresponding author(s): DUPREZ Delphine

Last updated by author(s): May 5, 2021

Reporting Summary

Nature Research wishes to improve the reproducibility of the work that we publish. This form provides structure for consistency and transparency in reporting. For further information on Nature Research policies, see our Editorial Policies and the Editorial Policy Checklist.

Statistics

For all statistical analyses, confirm that the following items are present in the figure legend, table legend, main text, or Methods section.

n/a	Confirmed	
☐	☒	The exact sample size (*n*) for each experimental group/condition, given as a discrete number and unit of measurement
☐	☒	A statement on whether measurements were taken from distinct samples or whether the same sample was measured repeatedly
☐	☒	The statistical test(s) used AND whether they are one- or two-sided<br>*Only common tests should be described solely by name; describe more complex techniques in the Methods section.*
☒	☐	A description of all covariates tested
☒	☐	A description of any assumptions or corrections, such as tests of normality and adjustment for multiple comparisons
☐	☒	A full description of the statistical parameters including central tendency (e.g. means) or other basic estimates (e.g. regression coefficient) AND variation (e.g. standard deviation) or associated estimates of uncertainty (e.g. confidence intervals)
☐	☒	For null hypothesis testing, the test statistic (e.g. *F*, *t*, *r*) with confidence intervals, effect sizes, degrees of freedom and *P* value noted<br>*Give P values as exact values whenever suitable.*
☒	☐	For Bayesian analysis, information on the choice of priors and Markov chain Monte Carlo settings
☒	☐	For hierarchical and complex designs, identification of the appropriate level for tests and full reporting of outcomes
☒	☐	Estimates of effect sizes (e.g. Cohen's *d*, Pearson's *r*), indicating how they were calculated

Our web collection on statistics for biologists contains articles on many of the points above.

Software and code

Policy information about availability of computer code

Data collection	Zen 2.0 (blue edition) / Zeiss ApoTome.2 microscope<br>LAS AF version 2.7.3.9723 / Leica SP5 AOBS confocal<br>Zen version 14.0.0.0 / Zeiss LSM700 confocal<br>StepOneSoftware / Applied Biosystems StepOnePlus Real-Time PCR system
Data analysis	Cell Ranger Single Cell Software Suite 3.0.2 by 10X Genomics<br>STAR21<br>Seurat version 3.0 (standard pipeline, code available upon request)<br>R version 3.6.1<br>Scrublet version 0.2.1<br>FIJI version 2.1.0<br>GraphPad Prism version 6

For manuscripts utilizing custom algorithms or software that are central to the research but not yet described in published literature, software must be made available to editors and reviewers. We strongly encourage code deposition in a community repository (e.g. GitHub). See the Nature Research guidelines for submitting code & software for further information.

Data

Policy information about availability of data

All manuscripts must include a data availability statement. This statement should provide the following information, where applicable:

- Accession codes, unique identifiers, or web links for publicly available datasets
- A list of figures that have associated raw data
- A description of any restrictions on data availability

Raw sequencing data has been deposited in the NCBI Gene Expression Omnibus database (https://www.ncbi.nlm.nih.gov/geo/) with the GEO data accession number GSE166981.

Field-specific reporting

Please select the one below that is the best fit for your research. If you are not sure, read the appropriate sections before making your selection.

☒ Life sciences ☐ Behavioural & social sciences ☐ Ecological, evolutionary & environmental sciences

For a reference copy of the document with all sections, see nature.com/documents/nr-reporting-summary-flat.pdf

Life sciences study design

All studies must disclose on these points even when the disclosure is negative.

Sample size	No statistical method was used to predetermine sample size. We used a sample size of at least 3 samples per experiment. Subsequent statistical analysis confirmed that our sample size allowed for statistically significant results.
Data exclusions	No data exclusion
Replication	scRNA-seq was performed in monoplicate. Embryo surgical manipulations and cell cultures were performed in at least triplicates. Cell and embryo samples were analysed (in situ hybridisation, immunostainings, RT-qPCR) at least in biological triplicates. All attempts at replications were successfull.
Randomization	Randomization is not relevant in our study since the groups (cell cultures or embryos) were compared between wild-type and gain-of-function conditions or since experiments were cell lineage tracings.
Blinding	Investigators were not blinded for data collection when samples needed to be allocated to wild-type or gain-of-function conditions. Investigators were blinded for subsequent data analysis. Blinding is not relevant for cell lineage tracing analysis.

Reporting for specific materials, systems and methods

We require information from authors about some types of materials, experimental systems and methods used in many studies. Here, indicate whether each material, system or method listed is relevant to your study. If you are not sure if a list item applies to your research, read the appropriate section before selecting a response.

Materials & experimental systems

n/a	Involved in the study	
☐	☒	Antibodies
☒	☐	Eukaryotic cell lines
☒	☐	Palaeontology and archaeology
☐	☒	Animals and other organisms
☒	☐	Human research participants
☒	☐	Clinical data
☒	☐	Dual use research of concern

Methods

n/a	Involved in the study	
☒	☐	ChIP-seq
☒	☐	Flow cytometry
☒	☐	MRI-based neuroimaging

Antibodies

Antibodies used	Mouse monoclonal IgG2b anti-MyHC (MF20) DSHB, Cat. # MF 20, RRID:AB_2147781, Produced in the lab<br>Mouse monoclonal IgG1 anti-Myod BD Biosciences, Cat. # 554130, RRID:AB_395255, lot # 9011506<br>Mouse monoclonal IgG1 anti-Myog DSHB, Cat. # F5D, RRID:AB_2146602, Produced in the lab<br>Mouse monoclonal IgG1 anti-Pax7 DSHB, Cat. # AB_PAX7, RRID:AB_528428, lot # 20ea1/24/19<br>Mouse monoclonal IgG1 anti-quail nuclei (QCPN) DSHB, Cat. # QCPN, RRID:AB_531886, Produced in the lab<br>Rabbit polyclonal anti-β-gal MP Biomedicals, Cat. # MP 55976, RRID:AB_2687418, lot # 6825

	Rabbit polyclonal anti-Collagen XII, Clone #522 from Manuel Koch, Koch et al., 1992<br>Rabbit polyclonal anti-Myog from Christophe Marcelle, Marceau et al., 2008, lot # HL1510<br>Rabbit polyclonal anti-PSMAD1/5/9 Cell Signaling, Cat. # 9516, RRID:AB_491015, lot # 9<br>Rabbit polyclonal anti-TCF4 Cell Signaling, Cat. # 2569, RRID:AB_2199816, lot # 4<br>Rabbit polyclonal anti-Tomato TAKARA, Cat. # 632496, RRID:AB_10013483, lot # 1612022<br>Chicken polyclonal anti-GFP Abcam, Cat. # ab13970, RRID:AB_300798, lot # GR3190550-33<br>Alexa Fluor® 555 Goat Anti-Mouse IgG Invitrogen, Cat. # A21422, RRID:AB_141822, lot # 514956<br>Alexa Fluor®488 AffiniPure Goat Anti-Mouse IgG1(γ1) Jackson ImmunoResearch Labs, Cat. # 115-545-205, RRID:AB_2338854, lot # 101249<br>Alexa Fluor® 633 Goat Anti-Mouse IgG1(γ1) Thermo Fisher Scientific, Cat. # A-21126, RRID:AB_2535768, lot # 1977327<br>Alexa Fluor® 647 Goat Anti-Mouse IgG1(γ1) Thermo Fisher Scientific, Cat. # Z 25008, RRID:AB_2535768, lot # 1915925<br>Cy3-AffiniPure Goat Anti-Mouse IgG1(γ1) Jackson ImmunoResearch Labs, Cat. # 115-165-205, RRID:AB_2338694, lot # 135078<br>Alexa Fluor® 488 Goat Anti-Mouse IgG2b(γ2) Invitrogen, Cat. # A21141, RRID:AB_141626, lot # 2228625 / 1723667<br>Alexa Fluor® 633 Goat Anti-Mouse IgG2b (γ2b) Thermo Fisher Scientific, Cat. # A-21146, RRID:AB_2535782, lots # 1786287<br>Alexa Fluor® 647 Goat Anti-Mouse IgG2b (γ2b) Thermo Fisher Scientific, Cat. # A-21242, RRID:AB_2535782, lot # 2155295<br>Alexa Fluor® 488 F(ab')2 Fragment of Goat Anti-Rabbit IgG Thermo Fisher Scientific, Cat. # A-11070, RRID:AB_2534114, lot # 1907301<br>Alexa Fluor® 555 F(ab')2 Fragment of Goat Anti-Rabbit IgG Thermo Fisher Scientific, Cat. # A-21430, RRID:AB_2535851, lot # 2184320<br>Alexa Fluor® 633 F(ab')2 Fragment of Goat Anti-Rabbit IgG Thermo Fisher Scientific, Cat. # A-21072, RRID:AB_2535733, lot # 1839600<br>Alexa Fluor® 488 Goat Anti-Chicken IgG (H+L) Thermo Fisher Scientific, Cat. # A-11039, RRID:AB_2534096, lot # 16911381 / 1812246
Validation	The primary antibodies used were stated to be "suitable", "validated" or "pusblished" by the manufacturers (see list below) or validated by the referenced publications for the species (quail, chicken and mouse) and application (IF).<br>Mouse monoclonal IgG2b anti-MyHC (MF20): IF, chick, quail, mouse: https://dshb.biology.uiowa.edu/MF-20<br>Mouse monoclonal IgG1 anti-Myod: IF, ouse: https://www.bdbiosciences.com/us/applications/research/stem-cell-research/mesoderm-markers/human/purified-mouse-anti-myod-58a/p/554130<br>Mouse monoclonal IgG1 anti-Myog: IF, mouse: https://dshb.biology.uiowa.edu/F5D<br>Mouse monoclonal IgG1 anti-Pax7: IF, chick, quail, mouse: https://dshb.biology.uiowa.edu/PAX7<br>Mouse monoclonal IgG1 anti-quail nuclei (QCPN): IF, quail: https://dshb.biology.uiowa.edu/QCPN<br>Rabbit polyclonal anti-β-gal: IF, E.coli: https://www.mpbio.com/eu/rabbit-igg-fraction-to-beta-galactosidase<br>Rabbit polyclonal anti-PSMAD1/5/9: IF, chick, quail: https://www.cellsignal.com/products/primary-antibodies/phospho-smad1-5-ser463-465-41d10-rabbit-mab/9516 and Wang H, et al. Dev Cell. 2010. PMID: 20412778<br>Rabbit polyclonal anti-TCF4: IF, chick, quail: https://www.cellsignal.com/products/primary-antibodies/tcf4-tcf7l2-c48h11-rabbit-mab/2569<br>Rabbit polyclonal anti-Tomato: IF, recombinant fragment.: https://www.takarabio.com/products/antibodies-and-elisa/fluorescent-protein-antibodies/red-fluorescent-protein-antibodies<br>Chicken polyclonal anti-GFP: IF, recombinant fragment: https://www.abcam.com/gfp-antibody-ab13970.html

Animals and other organisms

Policy information about studies involving animals; ARRIVE guidelines recommended for reporting animal research

Laboratory animals	Male and female adult mice (Mus musculus, 6 to 12 weeks of age). Mice were kept on mixed the genetic backgrounds C57BL/6JRj or DBA/2JRj (B6D2F1, Janvier).
Wild animals	None
Field-collected samples	None
Ethics oversight	Animals were handled as per European Community guidelines. Protocols were validated by the ethics committee of the French Ministry, under the reference numbers APAFIS#6354-20160809l2028839.v4, APAFIS#13695-2018021408521124.v2 and APAFIS#24357-2020041613396163.v3. and by the Institut Pasteur ethics committee (CETEA, reference 2015–0008).

Note that full information on the approval of the study protocol must also be provided in the manuscript.
